# Revealing fine-scale spatiotemporal differences in SARS-CoV-2 introduction and spread

**DOI:** 10.1038/s41467-020-19346-z

**Published:** 2020-11-03

**Authors:** Gage K. Moreno, Katarina M. Braun, Kasen K. Riemersma, Michael A. Martin, Peter J. Halfmann, Chelsea M. Crooks, Trent Prall, David Baker, John J. Baczenas, Anna S. Heffron, Mitchell Ramuta, Manjeet Khubbar, Andrea M. Weiler, Molly A. Accola, William M. Rehrauer, Shelby L. O’Connor, Nasia Safdar, Caitlin S. Pepperell, Trivikram Dasu, Sanjib Bhattacharyya, Yoshihiro Kawaoka, Katia Koelle, David H. O’Connor, Thomas C. Friedrich

**Affiliations:** 1 Department of Pathology and Laboratory Medicine, University of Wisconsin-Madison, Madison, WI USA; 2 Department of Pathobiological Sciences, University of Wisconsin-Madison, Madison, WI USA; 3 Population Biology, Ecology, and Evolution Graduate Program, Laney Graduate School, Emory University, Atlanta, GA USA; 4 Department of Biology, Emory University, Atlanta, GA USA; 5 Influenza Research Institute, School of Veterinary Sciences, University of Wisconsin-Madison, Madison, WI USA; 6 Wisconsin National Primate Research Center, University of Wisconsin-Madison, Madison, WI USA; 7 City of Milwaukee Health Department Laboratory, Milwaukee, WI USA; 8 University of Wisconsin School of Medicine and Public Health, Madison, WI USA; 9 The William S. Middleton Memorial Veterans Hospital, Madison, WI USA; 10 Department of Medicine, Division of Infectious Diseases, University of Wisconsin School of Medicine and Public Health, Madison, WI USA; 11 Department of Medical Microbiology and Immunology, University of Wisconsin-Madison, Madison, WI USA

**Keywords:** Phylogenetics, SARS-CoV-2, Viral infection, Epidemiology

## Abstract

Evidence-based public health approaches that minimize the introduction and spread of new SARS-CoV-2 transmission clusters are urgently needed in the United States and other countries struggling with expanding epidemics. Here we analyze 247 full-genome SARS-CoV-2 sequences from two nearby communities in Wisconsin, USA, and find surprisingly distinct patterns of viral spread. Dane County had the 12^th^ known introduction of SARS-CoV-2 in the United States, but this did not lead to descendant community spread. Instead, the Dane County outbreak was seeded by multiple later introductions, followed by limited community spread. In contrast, relatively few introductions in Milwaukee County led to extensive community spread. We present evidence for reduced viral spread in both counties following the statewide “Safer at Home” order, which went into effect 25 March 2020. Our results suggest patterns of SARS-CoV-2 transmission may vary substantially even in nearby communities. Understanding these local patterns will enable better targeting of public health interventions.

## Abstract (web summary)

In this study, the authors present an analysis of 247 full-genome SARS-CoV-2 sequences obtained from two communities in Wisconsin, USA, and report distinct patterns of viral spread. Their results suggest that patterns of SARS-CoV-2 transmission and spread may vary substantially, even between neighbouring communities.

## Introduction

The earliest outbreaks of severe acute respiratory syndrome coronavirus 2 (SARS-CoV-2) in the United States were seeded by travelers who became infected abroad and initiated chains of community transmission. Several months later, SARS-CoV-2 is now ubiquitous. More than 96% of the 3144 United States administrative subdivisions (i.e., counties, boroughs, and parishes) have reported at least one SARS-CoV-2 case by 23 June 2020^1^. Movement between administrative subdivisions and states, rather than introduction from abroad, now poses the greatest risk for seeding new clusters of community transmission. However, trends in SARS-CoV-2 caseload and spread are often reported on large geographic scales, such as US states, which obscures the degree to which trends may differ on smaller geographic scales. Finescale spatiotemporal patterns of SARS-CoV-2 spread, particularly below the level of a state or territory, remain poorly defined.

Case counts from diagnostic SARS-CoV-2 testing are used to understand community transmission, but community-level testing may not be widely available and passive surveillance is unlikely to detect asymptomatic or presymptomatic infections. Viral genome sequencing has emerged as a critical tool to overcome these limitations and provides a complementary means of understanding viral transmission dynamics. The value of this approach was demonstrated during the West African Ebolavirus outbreak in 2014–2016 and again during the emergence of Zika virus in the Americas in 2015–2016^2,3^.

The collective global effort to sequence SARS-CoV-2 dwarfs these earlier efforts. As of 28 June 2020, more than 55,000 SARS-CoV-2 sequences collected from over 82 countries have been sequenced and shared publicly on repositories like the Global Initiative on Sharing All Influenza Data (GISAID), enabling real-time phylogenetic analyses encompassing global SARS-CoV-2 diversity^4–6^. Patterns of viral sequence variation can also be used to estimate epidemiological parameters, including the total number of infections in a given population and epidemic doubling time, independent of case counts^4,7–16^. Here we apply these methods to gain a nuanced view of SARS-CoV-2 transmission within and between regions of the American Upper Midwest.

Dane and Milwaukee counties are the two most populous counties in the US state of Wisconsin. They are separated by approximately 100 km of rural and suburban communities in Jefferson and Waukesha counties. An interstate highway that typically carries ~40,000 vehicles a day connects all four of these counties^17^. Madison and Milwaukee are the largest cities in Wisconsin as well as in Dane and Milwaukee counties, respectively, and are demographically dissimilar^18,19^. On 25 March 2020, the Wisconsin Department of Health Services ordered most individuals to stay at home, closed non-essential businesses, and prohibited most gatherings, an order termed “Safer at Home”^20–22^. While there were some policies enacted to reduce the viral spread prior to this order^23^, the “Safer at Home” order represented the most significant restriction on individuals and businesses. This Executive Order remained in effect until 13 May 2020, when it was struck down by the Wisconsin Supreme Court. From the start of the Executive Order through 21 April 2020, Dane and Milwaukee counties had the highest documented number of SARS-CoV-2 cases in Wisconsin. Therefore, these two counties provide a “natural experiment” to understand the impact of the “Safer at Home” Executive Order on within- and between-county SARS-CoV-2 transmission in two US counties with distinguishing demographic features.

Here we use deeply sampled SARS-CoV-2 sequence data to characterize spread in southeastern Wisconsin and, more importantly, illustrate distinct patterns of spatiotemporal SARS-CoV-2 spread in two very nearby communities. We note that this study was not designed prospectively. Moreover, we find that the virus’s basic reproductive number decreased in both counties evaluated during the time in which the “Safer at Home” order was in place, consistent with adoption of physical distancing, use of face coverings, and other related practices^24^.

## Results

### SARS-CoV-2 epidemics and community demographics in Dane and Milwaukee counties

**Fig. 1:**
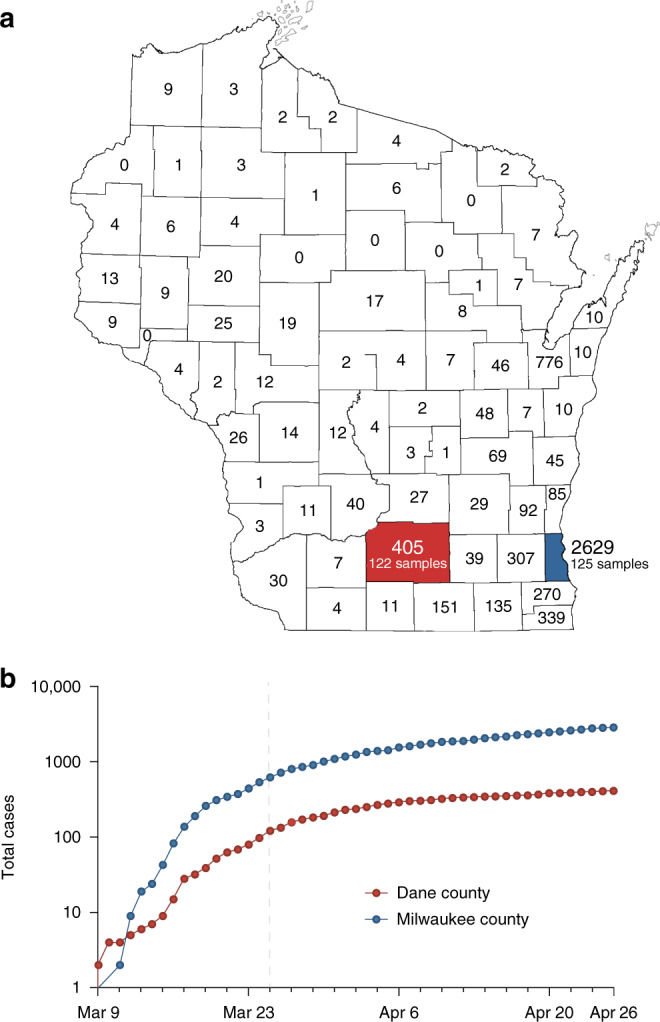
Demography and epidemiology of SARS-CoV-2 in southern Wisconsin. **a** A map of Wisconsin highlighting Dane county (red) and Milwaukee county (blue). Cumulative case counts through 26 April 2020 are reported within each county border. The map of Wisconsin’s county borders was obtained with copyright permissions from the Wisconsin State Cartographer’s Office—https://www.sco.wisc.edu/maps/. **b** Cumulative SARS-CoV-2 cases in Dane county (red) and Milwaukee county (blue) from 9 March through 26 April. The vertical dashed line indicates the start date of Wisconsin’s “Safer at Home” order, which went into effect from 25 March 2020 ^24^. Source data to replicate this figure can be found in the Source Data file.

Dane county is home to the 12th reported SARS-CoV-2 case in the United States, detected on 30 January 2020. Subsequent cases were not reported until 9 March 2020. By 26 April, Dane county had 405 confirmed SARS-CoV-2 cases and 19 deaths^25^. Milwaukee county reported its first case on 11 March 2020. By 26 April, Milwaukee county had reported 2629 confirmed SARS-CoV-2 infections and 126 deaths^26^ (Fig. 1b).

Dane county and Milwaukee county are both located in Southern Wisconsin. Milwaukee county is 127 km east of Dane county, measured from center to center. As of 2015, Dane county had a population of 516,850 at a density of 166 people per km^2^ compared to 952,150 at 1522 per km^2^ for Milwaukee county (Fig. 1a)^18,19^.

The majority of individuals living in Dane county are White (81.5%). The next largest group identifies as Hispanic or Latinx (6.3%), followed by Asian (6.0%), Black (5.9%), and American Indian (0.3%)^19^. Milwaukee county is less predominantly White (53.3%) with much larger Black (27.2%) and Hispanic or Latinx (14.5%) populations, followed by Asian (4.3%) and American Indian (0.7%)^18^. The percent of individuals ≥65 years old is similar in Dane county (13.7%) and Milwaukee county (13.6%), while the percent of individuals under 18 years is slightly lower in Dane county (20.4%) than Milwaukee county (24%). In addition, median income and access to healthcare resources is lower in Milwaukee county than in Dane county^27^. The median individual in Milwaukee county is also more likely to experience poverty and to live with comorbidities such as type II diabetes, hypertension, and obesity (Table 1)^27^.

**Table 1:** County-level demographics for Dane and Milwaukee county.

County-level demographic data	Dane	Milwaukee
Population size (2015)	516,850	952,150
Population per square mile (2015)	430	3942
Average number of persons per dwelling (2014–2018)	2.35	2.44
Age (2014–2018):		
% of population under 5	5.6	6.9
% of population under 18	20.4	24
% of population over 65	13.7	13.6
Race/ethnicity (2015):		
White	81.5%	53.3%
African American	5.9%	27.2%
American Indian	0.3%	0.7%
Hispanic	6.3%	14.5%
Asian	6.0%	4.3%
Median income (2015)	$65,416	$45,905
% of population that is uninsured, under 65 (2014–2018)	4.9%	8.2%
Poverty estimate, all ages (2015)	11.2%	20.3%
% of population reported overweight or obese (2012–2016)	54.3–58.5%	64.7–69%
% of adults reporting diagnosed diabetes (2012–2016)	4.2–6.8%	8.6–9.8%

### Dane and Milwaukee county viruses are genetically distinct

If an outbreak is fueled by community spread following a single introduction, one would expect viral genomes to be close phylogenetic relatives, in which case genetic distances measured in any pairwise comparisons of sequences would be low. To examine this, we generated SARS-CoV-2 consensus sequences using the ARTIC Network protocol^28,29^ and defined the population of consensus single-nucleotide variants (SNVs) relative to the initial SARS-CoV-2 Wuhan reference (Genbank: MN908947.3).

**Fig. 2:**
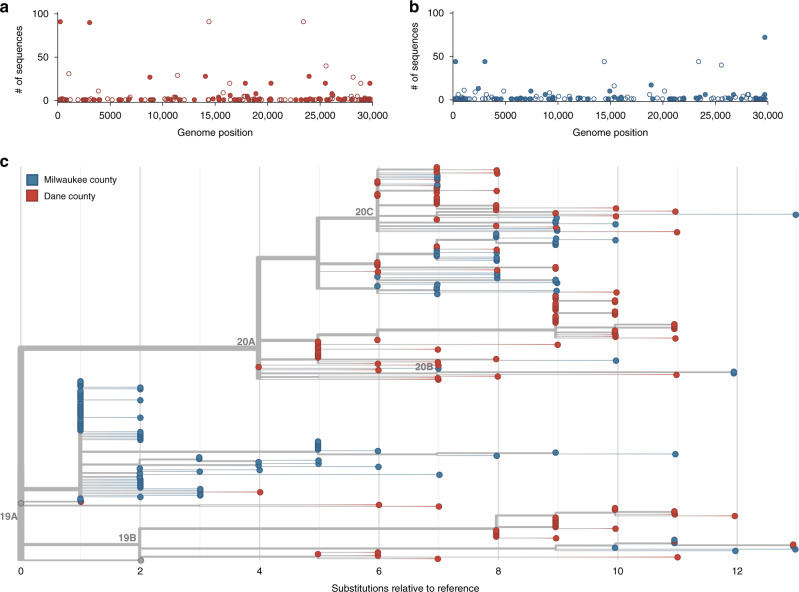
Characterizing consensus-level variants and sequence divergence among Dane and Milwaukee county sequences. Single-nucleotide variants (SNVs) are annotated relative to the initial Wuhan SARS-CoV-2 reference (Genbank: MN908947.3). **a** Frequency of consensus SNVs among the Dane county sequences (red). **b** Frequency of consensus SNVs among the Milwaukee county sequences (blue). Open symbols denote synonymous or intergenic SNVs and closed symbols denote nonsynonymous SNVs. **c** A divergence-based phylogenetic tree built using Nextstrain tools for the 122 Dane county (red) and 125 Milwaukee county (blue) sequences. Wisconsin samples are rooted against Wuhan-Hu-1/2019 and Wuhan/WH01/2019. Source data to replicate this figure can be found in the Source Data file.

In Dane county, we identified 155 distinct SNVs across 122 samples evaluated. These SNVs are evenly distributed throughout the genome, and 92.9% (144/155) are located in open reading frames (ORFs). In Dane county, 52.9% (82/155) of consensus SNVs result in an amino acid change (nonsynonymous) and 40% (62/155) do not (synonymous) (Fig. 2a).

In Milwaukee county, we identified 148 distinct SNVs across 125 samples evaluated. Among the observed consensus SNVs in Milwaukee county, 63.5% (94/148) are nonsynonymous and 31.8% (47/148) are synonymous (Fig. 2b).

Mean inter-sequence pairwise SNV distance was 7.65 (std 1.83) and 5.02 (std 3.63) among Dane county and Milwaukee county sequences, respectively (Fig. 2c). Likewise, we detected an average of 4.4 new SNVs per day (sampling period of 35 days) in Dane county and 3.6 new SNVs per day (sampling period of 41 days) in Milwaukee county. Previous reports suggested SARS-CoV-2 is expected to acquire approximately one fixed SNV every 15 days following a single introduction^30^. Compared to this benchmark, both Dane county and Milwaukee county have “excess” diversity which can be most parsimoniously explained by multiple introductions of divergent viruses. These patterns are consistent with a greater number of introductions of distinct viruses into Dane county compared to Milwaukee county.

**Fig. 3:**
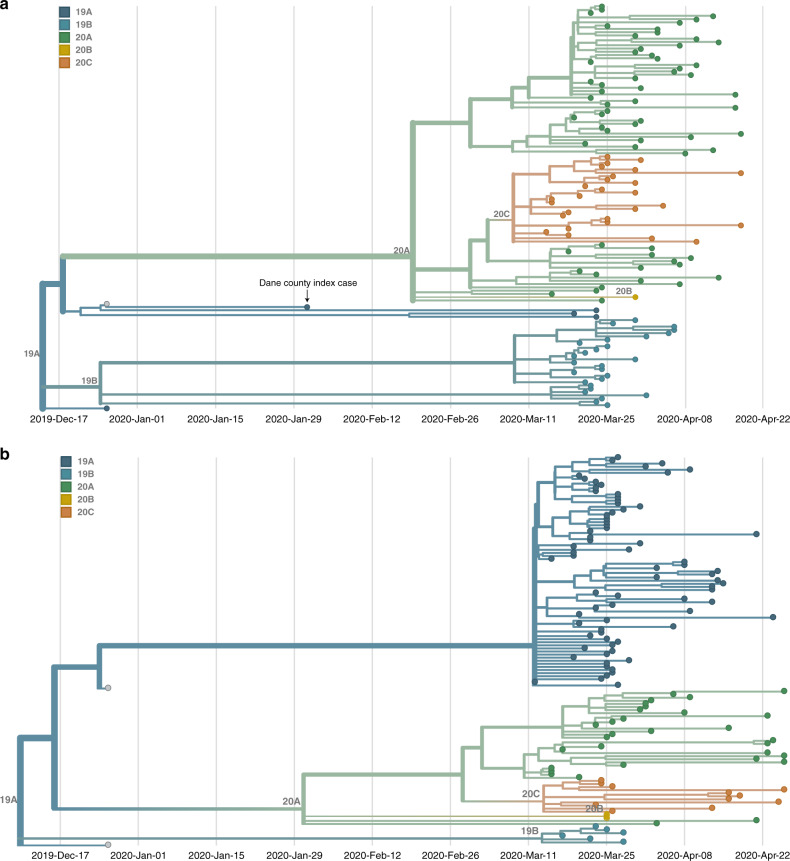
Dane and Milwaukee county outbreaks are defined by genetically distinct viruses. **a** A time-resolved phylogenetic tree built using Nextstrain tools for 122 samples collected in Dane county. **b** A time-resolved phylogenetic tree for 125 samples collected in Milwaukee county. Clade is denoted by color where dark blue denotes 19A, aqua denotes 19B, green denotes 20A, gold denotes 20B, and orange denotes 20C. Both phylogenies include Wuhan sequences (Wuhan-Hu-1/2019 and Wuhan/WH01/2019, shown in gray) to more effectively time-align each tree. Source data to replicate this figure can be found in the Source Data file.

To further analyze the genetic differences among viruses in the two locations, we assigned clades using the Nextstrain nomenclature. For example, clade 19B is defined by two mutations at nucleotides 8782 (ORF1ab S2839S) and 28,144 (Spike L84S) relative to a reference SARS-CoV-2 isolate from Wuhan, China (Genbank: MN908947.3). The majority of Dane county sequences (*n* = 63 sequences; 51.6%) cluster in the 20A clade (Fig. 3a). This clade is defined by four variants, at nucleotide positions 241 (upstream of the first ORF), 3037 (ORF1a F924F), 14,408 (ORF1b P314L), and 23,403 (S D614G). A minority (*n* = 31 sequences; 24.8%) of Milwaukee county sequences also cluster in this clade. In contrast, the 19A clade designation is most common (*n* = 75 sequences; 60.0%) in sequences from Milwaukee county. This clade is distinguished by a U-to-C variant at nucleotide position 29,711 (downstream of ORF10) (Fig. 3b).

### No onward spread from Dane county index case

The first known SARS-CoV-2 case in Wisconsin was a person who was likely infected during travel to Wuhan, Hubei province, China, where they were exposed to family members with confirmed SARS-CoV-2 infections. The patient reported a sore throat shortly before departing China and returning to the US on 30 January 2020. The person wore a mask during the return flight. Upon arrival in the US, the person immediately presented to an emergency department while still wearing a mask. The person was afebrile and had no respiratory or gastrointestinal signs or symptoms, but began to develop mild respiratory symptoms shortly thereafter. The person’s condition remained stable and never required hospitalization or advanced care, with symptoms resolving 5 days later. The person self-quarantined in an isolated room in a home with a dedicated bathroom for 30 days following symptom onset. During this time, nasopharynx samples repeatedly tested positive for SARS-CoV-2 viral RNA.

Documentation of asymptomatic infections of SARS-CoV-2 has led to concerns about the role of cryptic community transmission in the United States^9,31,32^. However, sequencing in other locations in the United States has revealed early introduction events did not always go on to seed downstream community spread^33^. To determine whether SARS-CoV-2 cases detected in Dane county in March might have been due to undetected spread from the first Wisconsin introduction, we compared the sequence of this early case with local and global SARS-CoV-2 sequence diversity. The first Dane county patient’s virus contains an in-frame deletion at nucleotide positions 20,298–20,300, in a region that codes for the poly(U)-specific endoribonuclease; the impact of this mutation on viral fitness is unknown^34^ (Supplementary Fig. 1). Notably, this deletion was not detected in any other Dane county sequence, nor in any other sample(s) submitted to GISAID as of 18 April 2020. Moreover, there are no branches originating from the index Dane county case on either the global (Wisconsin sequences plus a subsampled set of global sequences) or local phylogenies (Wisconsin sequences only, maximum likelihood) (Figs. 2c and 3a). Thus, this early case appears to be an example of successful infection control practices.

### SARS-CoV-2 outbreak dynamics differ between Milwaukee and Dane counties

**Fig. 4:**
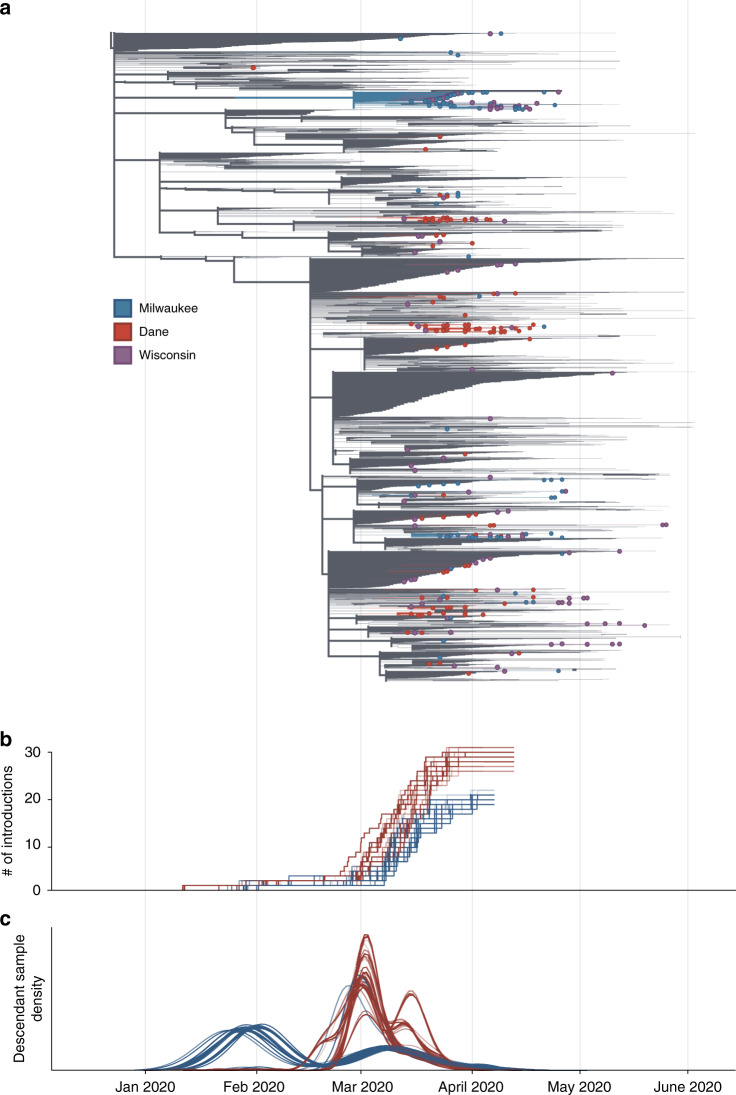
Estimate of the number of introduction events into Milwaukee and Dane county and their relative contribution to downstream epidemic dynamics. **a** Maximum likelihood (ML) time-resolved tree, supported by 1000 bootstrap replicates, with subsampled global sequences and closest phylogenetic neighbors’ relatives included (gray branches). Sequences from Dane and Milwaukee counties are highlighted in red and blue, respectively. Sequences with geolocation information available to the state level, or that are located outside of Dane and Milwaukee counties (i.e. La Crosse) are shown in purple. **b** Estimated cumulative number of introduction events into each county (derived using 100 bootstrap replicate trees). **c** Gaussian Kernel Density Estimate plots showing the estimated timing of each introduction event (three curves per replicate: mean and 90% confidence intervals) into Dane county (red) or Milwaukee county (blue). The relative number of samples from each region attributable to an introduction event is represented on the *y* axis. Curves are normalized to a cumulative density of one; therefore, *y* axis scale is not shown. Source data to replicate this figure can be found in the Source Data file.

The independent local phylogenies in Dane and Milwaukee county suggested that these two nearby locations had largely distinct SARS-CoV-2 epidemics through April 2020. To better understand the number of introductions and continued transmission dynamics, we generated a time-resolved subsampled global phylogeny incorporating Dane county (red tips) and Milwaukee county (blue tips) sequences alongside representative global SARS-CoV-2 sequences, including all other available Wisconsin sequences (purple tips) (Fig. 4a). Dane county viruses are distributed throughout the tree, consistent with multiple unique introductions. In contrast, Milwaukee county viruses cluster more closely together, consistent with fewer introductions leading to subsequent community transmission.

To estimate the number of introductions into the state and subsequently each county, we used an ancestral state reconstruction of internal nodes. We performed 100 bootstrap replicates to account for uncertainty in the phylogenetic inference. This yielded an estimate of 59 [59, 63] (median [95% highest density interval (HDI)]) independent introductions of SARS-CoV-2 into the state of Wisconsin. Of these, 29 [28, 31] led to introductions into Dane county whereas only 21 [19, 21] led to introductions into Milwaukee county (Fig. 4b). Surprisingly, only 9 [6, 10] of the introductions into Wisconsin were associated with sequences from both counties. Furthermore, these shared introductions accounted for only 20–30% of the samples from Dane and Milwaukee county present in our dataset. Together, our analyses suggest that transmission between Dane and Milwaukee counties has not been a principal component of viral spread within either region. We find that local transmission in Milwaukee county began earlier, with an introduction event in late January/early February leading to a large number of the Milwaukee county sequences (Fig. 4c). In comparison, most samples collected from Dane county are associated with multiple introductions in late February/early March (Fig. 4c). Despite the fact that there were more introductions into Dane county, the reported number of cases was considerably less than in Milwaukee county. This indicates that each introduction into Dane county contributed less to onward viral transmission than in Milwaukee county.

To account for sampling bias on our estimates, we randomly sampled sequences from our set of Dane and Milwaukee county samples (*N* = 20–240, increments of 20) and pruned all other Dane and Milwaukee samples from the maximum likelihood tree. This was repeated ten times for each N, creating a set of 120 trees. We repeated the ancestral state reconstruction on each of these trees and re-estimated the number of introductions (Supplementary Fig. 2). The number of estimated introductions into Dane county continued to increase with the number of sampled sequences, indicating that these data may be undersampling the true number of circulating viral lineages. In contrast, the number of estimated introductions into Milwaukee county decreases more slowly than Dane county, consistent with a small number of introductions. However, we cannot entirely rule out the possibility that the small number of introductions in Milwaukee county may be an artifact of biased sampling, where the available sequences may only represent a portion of the transmission chains and not a true estimation of the total circulating viral population. Because of this, the true number of introductions is likely to change as more sequences become available in each county. Taken together, these results suggest that patterns of SARS-CoV-2 introduction and spread can differ dramatically in two small administrative regions (here, Dane and Milwaukee counties), despite their close geographic, economic, and political connections.

### Spread of SARS-CoV-2 was reduced following Wisconsin’s “Safer at Home” order

We next used viral sequence data to assess the impact of Wisconsin’s “Safer at Home” order on SARS-CoV-2 transmission by estimating the basic reproduction number (*R*_0_). Transmission heterogeneity, or superspreading, is thought to play an important role in SARS-CoV-2 epidemics^11,35,36^. We therefore modeled *R*_0_ before and after the “Safer at Home” order in scenarios in which the level of transmission heterogeneity was low, medium, or high. In both counties, under all three scenarios, *R*_0_ fell by at least 40% after 25 March, indicating that the sequencing data support the observed decline in reported cases. In Dane county, estimated median *R*_0_ was reduced by 40% [4, 74], 49% [13, 79], and 60% [30, 83] under low, medium, and high transmission heterogeneity, respectively. Similarly, in Milwaukee county, estimated median *R*_0_ was reduced by 68% [50, 83], 71% [56, 86], and 72% [60, 84] under low, medium, and high transmission heterogeneity, respectively.

**Fig. 5:**
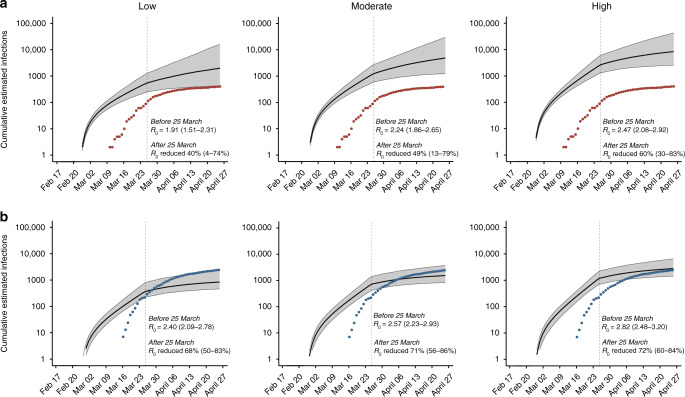
Phylodynamic modeling of regional outbreaks informs regional outbreak dynamics before and after government interventions. Bayesian phylodynamic modeling of cumulative incidence up to 26 April for outbreaks in **a** Dane county and **b** Milwaukee county under low (left), medium (center), and high (right) transmission heterogeneity conditions. Model parameters for low, medium, and high transmission heterogeneity were fixed such that 20%, 10%, and 5% of superspreading events contribute 80% of cumulative infections, respectively. Median cumulative incidence (solid black line) is bound by the 95% confidence intervals (CI; gray ribbon). Dots represent reported cumulative positive tests in Dane county (red) and Milwaukee county (blue). Estimated median reproductive numbers (*R*_0_) with 95% HDI are listed for the period before the Wisconsin “Safer at Home” order was issued on 25 March 2020. Percent reduction in *R*_0_ with 95% HDI is provided for the period after 25 March 2020. Each analysis presented here was run in duplicate for at least 3 million states in BEAST2 (see “Methods” for more details). Source data to replicate this figure can be found in the Source Data file.

In Dane county, estimated cumulative incidence was best predicted with the medium transmission heterogeneity model, based on alignment with reported incidence (Fig. 5a), whereas Milwaukee county’s cumulative incidence was best predicted with the model using high transmission heterogeneity (Fig. 5b). A greater role for superspreading events in Milwaukee versus Dane county could be explained by higher population density, higher poverty rates, and/or worse healthcare access (Table 1), all of which may increase contact rates and impede physical distancing efforts^36–40^. Assuming moderate transmission heterogeneity in Dane county, estimated *R*_0_ prior to 25 March was 2.24 [1.86, 2.65] and the median estimated cumulative incidence at the end of the study period (26 April) was 4546 infections [1187, 23,709] compared to 405 positive tests. In contrast, assuming high transmission heterogeneity in Milwaukee county, estimated *R*_0_ prior to 25 March was 2.82 [2.48, 3.20] and the median cumulative incidence on 26 April was only 3008 infections [1483, 7508] compared to 2629 positive tests.

With passive SARS-CoV-2 surveillance efforts in both counties likely missing subclinical and asymptomatic SARS-CoV-2 infections, we expect the true cumulative incidence to be considerably greater than the reported incidence, as has been suggested by others^41^. Indeed, estimated cases were ~10× higher than reported cases in Dane county. Given that there were no substantial differences in the surveillance efforts between counties, we expected more than the 1.1-fold difference in estimated and reported cases in Milwaukee county. Nearly equivalent estimated and reported cumulative incidence in Milwaukee county could be explained by better detection rates, inaccurate model parameters, and/or biased sampling. However, we likely have representative sampling across Milwaukee county, just on a smaller scale in comparison to Dane county. In an effort generate representative sequence data from Milwaukee county, samples were collected from over 35 zip codes and included samples from known outbreaks, community centers, healthcare facilities, congregate settings (long-term care facilities, jails, correction facilities), meat processing/packing plants as well as households in hotspots where SARS-CoV-2 transmission was detected within Milwaukee county (Supplementary Fig. 4). With better detection rates, a greater proportion of actual infections would be reported, but given the similar surveillance efforts between counties we expect detection rates to be comparable. Another possible explanation we cannot rule out is that different model parameters are required to more accurately model Milwaukee county’s epidemic. Our testing of three superspreading scenarios demonstrated that the superspreading parameters, at least, may be county-specific. In the case of biased sampling, where the available sequences only represent a portion of transmission chains in the county, our model would only estimate the caseload resulting from a subset of transmission chains in Milwaukee county and would underestimate the county-wide caseload. In support of representative county-wide sampling in Dane, but not Milwaukee county, sequences from 26.4% (107/405) of test-positive cases in Dane county, but only 3.9% (117/3008) of test-positive cases in Milwaukee county were available for phylodynamic modeling^25,26^.

## Discussion

A clear understanding of SARS-CoV-2 transmission patterns in a given location may permit and promote more effective targeting of public health messaging and infection mitigation efforts. Several studies have described how SARS-CoV-2 entered and began circulating within broad geographic regions, like entire countries (England, Brazil, Austria, Australia) or large and populous US states (Bay Area, NYC)^9–12,42–45^. But few studies to date have explored how such patterns may differ on finer geographic and temporal scales, even though many interventions will necessarily be highly localized in scope. Here, we examined differences in SARS-CoV-2 introduction and spread in two nearby counties—Dane county and Milwaukee county—as an example of how such patterns may differ even on small geographic scales. Dane county, Wisconsin had one of the earliest detected cases of SARS-CoV-2 infection in the United States, but this infection did not spark community spread. This is probably due to a combination of good infection control practices by healthcare providers, the patient, and sheer luck. Since March 2020 we find evidence for extensive introductions of SARS-CoV-2 into Dane county, none of which led to large-scale transmission clusters by the end of April 2020. As of 18 August 2020, Dane county has had a cumulative prevalence of 124.9 cases per 100,000 residents. In contrast, Milwaukee county, a larger and more densely populated region ~100 km away, has had 2627 cases per 100,000 residents as of 18 August 2020^46^. Our findings suggest that Milwaukee county’s higher caseload stems from greater levels of community spread descendant from fewer introduction points than in Dane county. Strikingly, we see little evidence for mixing of virus populations between these two closely linked communities in the same US state.

We used patterns of SARS-CoV-2 diversification in a phylodynamic model to estimate the initial reproductive rate of infections in each county before official physical distancing policies were enacted. In this initial phase of the outbreak, the median estimated *R*_0_ trended lower in Dane county than in Milwaukee county (2.24 vs 2.82). Higher overall population density and a higher average number of individuals residing in one dwelling in Milwaukee county could have contributed to a higher reproductive rate and greater community spread. A potential additional explanation for greater community spread is that the average individual in Milwaukee county, compared to Dane county, has access to fewer financial and healthcare resources and is more likely to experience poverty and to live with comorbid conditions, many of which are also risk factors for testing positive for SARS-CoV-2, the latter of which are also risk factors for severe COVID-19^18,19,47,48^. Additionally, Milwaukee county is home to a higher proportion of Black and Hispanic or Latinx individuals compared to Dane county. Because of race-based discrimination, people belonging to these groups experience worse health outcomes than White individuals, on average, despite being treated in the same healthcare systems^18,19,49,50^. The social vulnerability index (SVI) is a metric ranging designed to determine how resilient a community is when confronted with external stressors like natural disasters or a pandemic^51^. A higher SVI indicates a community is vulnerable to experiencing worsened outcomes secondary to an external stressor (range 0−1). All of the factors mentioned above contribute to a higher SVI in Milwaukee county (0.8268) compared to Dane county (0.1974)^51^. While the association between SVI and SARS-CoV-2 incidence is not significant, according to a recent study, the SVI components of socioeconomic and minority status are both predictors of higher SARS-CoV-2 incidence and case fatality rates^52^. These sub-components are likely to be among the main drivers in the outbreak dynamics between Dane and Milwaukee county.

Like most US states, in late March 2020 Wisconsin enacted a set of physical distancing policies aimed at reducing the spread of SARS-CoV-2. Wisconsin’s order, termed “Safer at Home,” was enacted on 25 March 2020. After this timepoint, the estimated *R*_0_ was reduced by 40% or more in both counties. The sequencing data are consistent with the observed reduction in positive tests, as clusters expanded more slowly and new clusters arose more slowly. Throughout this time, we find that the Dane county and Milwaukee county outbreaks were largely independent of one another. Our data reveal only limited mixing of SARS-CoV-2 genotypes between these geographically linked communities, supporting the notion that public health policies emphasizing physical distancing effectively reduce transmission between communities. Notably, “Safer at Home” ended abruptly on 13 May 2020, when it was overturned by the Wisconsin Supreme Court. Additional sequencing and epidemiological data will be necessary to understand whether virus intermingling between these counties increased after the cessation of the Executive Order.

Viral determinants could also affect differential transmission patterns within and between Dane and Milwaukee counties. If variants with greater transmission potential exist, then early introductions of such a variant into a community could contribute to greater spread there. Recent reports have suggested that a point mutation in the SARS-CoV-2 spike protein-encoding an aspartate-to-glycine substitution at amino acid residue 614 (D164G) may enhance transmissibility^53–55^. This mutation confers increased infectivity of pseudotyped murine retroviruses in ACE2-expressing HEK293T cells^55^ and has been proposed to be increasing in global prevalence, perhaps under natural selection^56^. Importantly, however, the rise in D614G frequency could also be due to founder effects, as viruses bearing the glycine allele may have been the first to establish local transmission in Europe. D614G is one of the mutations defining the 20A clade; these viruses remain dominant in Europe^33^, so introductions from Europe into the United States, including into Dane county, predominantly carry D614G. In comparison, in Milwaukee county, the vast majority of viruses have an aspartic acid residue at this site despite much higher levels of community transmission early in the pandemic. This observation does not necessarily indicate that D614G does not impact viral transmissibility; its role may be muted by other determinants of transmission, including demographic and socioeconomic factors. Viruses encoding D614G may displace 614D variants over time in regions like Milwaukee county, where 614D viruses have sustained community spread.

There are some important caveats to this study. Of the total reported positives in each county during the study period, high-quality sequences were available for 30% of test-positive cases in Dane county, but only 5% of test-positive cases in Milwaukee county^25,26^. Despite the deep sampling of SARS-CoV-2 sequences in Wisconsin relative to other regions in the US, even greater targeted sequencing efforts may be required to fully capture the sequence heterogeneity conferred by multiple introduction events and variable superspreading dynamics. It is possible additional sequencing in Milwaukee county would uncover additional viral lineages, or that the 5% of cases we sequenced do not fully represent the diversity of viruses found throughout the county, skewing our observations. However, in analyzing sample metadata, we find no evidence that particular locations within Milwaukee county were dramatically over- or under-sampled relative to their known SARS-CoV-2 prevalence (Supplementary Fig. 4). Another potential explanation is that Milwaukee county was under-testing relative to Dane county. Throughout the period analyzed here, the percentage of SARS-CoV-2 tests returning positive in Milwaukee county was ~20%, compared to only ~5% in Dane county^25,26^, indicating that a higher proportion of infections might have been missed by testing in Milwaukee county relative to Dane county. As we are only able to sequence test-positive samples, it is possible that under-testing in Milwaukee county limited our ability to capture a complete representation of their epidemic. However, we have no reason to suspect Milwaukee testing regimes were biased toward or against subsets of the overall population. During this time, there were three free community testing sites (supported by the Wisconsin National Guard) and several additional community testing and shelter sites located throughout the city. COVID-19 testing criteria for Milwaukee public health laboratories targeted all sectors of the population per Wisconsin Department of Health Services guidelines^57^. In sum, we have taken steps to minimize systematic sampling bias in Milwaukee county in this study, but we cannot entirely exclude the possibility that the samples available to us for sequencing did not fully capture the diversity of SARS-CoV-2 circulating in Milwaukee county during the study period.

It is also possible that other sequences from these counties relevant to our analyses were collected by other groups. As of 21 June 2020, there were 477 Wisconsin sequences available, but only 351 of these had geolocation information resolved to the county level. Some of the remaining 126 sequences likely originated from Dane county or Milwaukee county, but we cannot include these sequences in our analysis given their geolocation data resolved only to the state level. Currently there is no clearly stated national-level guidance for metadata to be associated with pathogen sequences. Dates and geographic locations with greater than state-level resolution are required to track the emergence and spread of novel pathogens like SARS-CoV-2. Explicit regulatory guidance from federal authorities enabling the disclosure of sequencing data with county-level geolocation data and sampling dates would enable other institutions to harmonize reporting of viral sequences and improve subsequent studies comparing viral sequences from different locations, as described previously^58^. Such reporting may be especially important for identifying disparities in viral transmission due to socioeconomic vulnerabilities in specific counties that would otherwise be masked using state-level data reporting.

Few previous studies have carefully evaluated patterns of SARS-CoV-2 introduction and spread below the level of US regions or states. Yet, with little US federal guidance, the majority burden of organizing and implementing anti-SARS-CoV-2 public health campaigns has fallen to US cities and counties. Tailoring public health messaging and intervention strategies to specific communities and locations can enhance their efficacy and durability. Our study exemplifies how viral sequence dynamics can enhance our understanding of the finescale patterns of virus introduction and spread, revealing differences in transmission patterns between even nearby communities that could inform the design of targeted interventions. For example, our data suggest Dane county, which had a large number of introductions but relatively little sustained community spread during the study period, might have benefited most from travel restrictions and/or quarantine for people entering the community. In contrast, our data suggest that community spread was established early in the study period in Milwaukee county, so interventions targeted at interrupting transmission clusters might have had the most impact. These could include limiting indoor community gatherings, targeting messaging or social marketing campaigns promoting mask-wearing and other physical distancing measures, and improving access to economic and healthcare resources—not only direct access to care, but also paid leave and other support systems for workers who are ill. To this end, continued efforts to sequence SARS-CoV-2 viruses across multiple spatiotemporal scales remain critical for tracking viral transmission dynamics within and between communities and for guiding “precision medicine” public health interventions to suppress future SARS-CoV-2 outbreaks.

## Methods

### Sample approvals and sample selection criteria

Sequences for this study were derived from 247 nasopharyngeal (NP) swab samples collected from Dane county between 14 March 2020 through 18 April 2020, and Milwaukee county from 12 March 2020 though 26 April 2020, Wisconsin. Most samples originated from the University of Wisconsin Hospital and Clinics and the Milwaukee Health Department Laboratories. Available sample metadata, including GISAID accession identifiers, are available in Supplementary Table 3.

We worked with residual diagnostic specimens in a biosafety level-3 containment laboratory at the AIDS Vaccine Research Laboratory at the University of Wisconsin-Madison. We obtained a waiver of HIPAA Authorization and were approved to obtain the clinical samples along with a Limited Data Set by the Western Institutional Review Board (WIRB #1-1290953-1). This limited dataset comprised sample collection data and county of collection. Additional sample metadata, e.g. race/ethnicity and income, were not shared.

Sample inclusion criteria were retrospectively applied and were threefold: (1) sample had a high-quality consensus sequence (passing GISAID quality control filters), (2) county of origin was Dane county or Milwaukee county, and (3) collection date was on or before our defined endpoint, 18 April 2020.

### vRNA isolation for the first confirmed SARS-CoV-2 case in Dane county

The first confirmed case of SARS-CoV-2 in Dane county occurred on 30 January 2020. This early sample was processed using an early iteration of our SARS-CoV-2 sequencing protocol, as outlined here. All other samples included in this study were processed using a modified-version of the ARTIC-sequencing protocol, as outlined below. Approximately 140 µL of VTM was passed through a 0.22 µm filter (Dot Scientific, Burton, MI, USA). Total nucleic acid was extracted using the Qiagen QIAamp Viral RNA Mini Kit (Qiagen, Hilden, Germany), substituting carrier RNA with linear polyacrylamide (Invitrogen, Carlsbad, CA, USA) and eluting in 30 µL of nuclease free H_2_O. The sample was treated with TURBO DNase (Thermo Fisher Scientific, Waltham, MA, USA) at 37 °C for 30 min and concentrated to 8 µL using the RNA Clean & Concentrator-5 kit (Zymo Research, Irvine, CA, USA). The full protocol for nucleic acid extraction and subsequent cDNA generation is available at https://www.protocols.io/view/sequence-independent-single-primer-amplification-o-bckxiuxn.

### Complementary DNA (cDNA) generation for first confirmed SARS-CoV-2 case in Dane county

Complementary DNA (cDNA) was synthesized using a modified Sequence Independent Single Primer Amplification (SISPA) approach described by Kafetzopoulou et al.^59,60^. RNA was reverse-transcribed with SuperScript IV Reverse Transcriptase (Invitrogen, Carlsbad, CA, USA) using Primer A (5′-GTT TCC CAC TGG AGG ATA-(N_9_)-3′). Reaction conditions were as follows: 1 µL of primer A was added to 4 µL of sample RNA, heated to 65 °C for 5 min, then cooled to 4 °C for 5 min. Then 5 µL of a master mix (2 μL 5× RT buffer, 1 μL 10 mM dNTP, 1 μL nuclease free H_2_O, 0.5 μL 0.1 M DTT (dithiothreitol), and 0.5 μL SSIV RT) was added and incubated at 42 °C for 10 min. For generation of second strand cDNA, 5 µL of Sequenase reaction mix (1 μL 5× Sequenase reaction buffer, 3.85 μL nuclease free H_2_O, 0.15 μL Sequenase enzyme) was added to the reaction mix and incubated at 37 °C for 8 min. This was followed by the addition of a secondary Sequenase reaction mix (0.45 μL Sequenase Dilution Buffer, 0.15 μL Sequenase Enzyme), and another incubation at 37 °C for 8 min. Following incubation, 1 µL of RNase H (New England BioLabs, Ipswich, MA, USA) was added to the reaction and incubated at 37 °C for 20 min. Conditions for amplifying Primer-A labeled cDNA were as follows: 5 µL of primer-A labeled cDNA was added to 45 µL of AccuTaq master mix per sample (5 µL AccuTaq LA 10× Buffer, 2.5 µL dNTP mix, 1 µL DMSO (dimethyl sulfoxide), 0.5 µL AccuTaq LA DNA Polymerase, 35 µL nuclease free water, and 1 µL Primer B (5′-GTT TCC CAC TGG AGG ATA-3′). Reaction conditions for the PCR were: 98 °C for 30 s, 30 cycles of 94 °C for 15 s, 50 °C for 20 s, and 68 °C for 2 min, followed by 68 °C for 10 min.

### vRNA isolation

As SARS-CoV-2 cases began to increase in Dane and Milwaukee counties, we adjusted our sequencing protocol. All samples from 10 March onward were isolated using a Maxwell isolation instrument and subsequently processed using a modified ARTIC tiled amplicon approach^28,29^. Nasopharyngeal swabs received in transport medium (VTM) were briefly centrifuged at 21,130 × *g* for 30 s at room temperature to ensure all residual sample sediments at the bottom of the tube. Viral RNA (vRNA) was extracted from 100 μL of VTM using the Viral Total Nucleic Acid Purification kit (Promega, Madison, WI, USA) on a Maxwell RSC 48 instrument and was eluted in 50 μL of nuclease free H_2_O.

### Complementary DNA (cDNA) generation

Complementary DNA (cDNA) was synthesized using a modified ARTIC Network approach^28,29^. Briefly, vRNA was reverse-transcribed with SuperScript IV Reverse Transcriptase (Invitrogen, Carlsbad, CA, USA) using random hexamers and dNTPs. Reaction conditions were as follows: 1 μL of random hexamers and 1 µL of dNTPs were added to 11 μL of sample RNA, heated to 65 °C for 5 min, then cooled to 4 °C for 1 min. Then 7 μL of a master mix (4 μL 5× RT buffer, 1 μL 0.1 M DTT, 1 µL RNaseOUT RNase Inhibitor, and 1 μL SSIV RT) was added and incubated at 42 °C for 10 min, 70 °C for 10 min, and then 4 °C for 1 min.

### Multiplex PCR to generate SARS-CoV-2 genomes

A SARS-CoV-2-specific multiplex PCR for Nanopore sequencing was performed, similar to amplicon-based approaches as previously described^28,29^. In short, primers for 96 overlapping amplicons spanning the entire genome with amplicon lengths of 500 bp and overlapping by 75−100 bp between the different amplicons were used to generate cDNA. Primers used in this manuscript were designed by the ARTIC Network and can be found in Supplementary Table 4. cDNA (2.5 μL) was amplified in two multiplexed PCR reactions with Q5 Hot-Start DNA High-fidelity Polymerase (New England Biolabs, Ipswich, MA, USA) using the the following cycling conditions: 98 °C for 30 s, followed by 25 cycles of 98 °C for 15 s and 65 °C for 5 min, followed by an indefinite hold at 4 °C ^28,29^. Following amplification, samples were pooled together for ONT library prep.

### Library preparation and sequencing

Amplified PCR product was purified using a 1:1 concentration of AMPure XP beads (Beckman Coulter, Brea, CA, USA) and eluted in 30 μL of water. PCR products were quantified using Qubit dsDNA high-sensitivity kit (Invitrogen, USA) and were diluted to a final concentration of 1 ng/μL. A total of 5 ng for each sample was then made compatible for deep sequencing using the one-pot native ligation protocol with Oxford Nanopore kit SQK-LSK109 and its Native Barcodes (EXP-NBD104 and EXP-NBD114)^29^. Specifically, samples were end-repaired using the NEBNext Ultra II End Repair/dA-Tailing Module (New England Biolabs, Ipswich, MA, USA). Samples were then barcoded using 2.5 µL of ONT Native Barcodes and the Ultra II End Repair Module. After barcoding, samples were pooled directly into a 1:1 concentration of AMPure XP beads (Beckman Coulter, Brea, CA, USA) and eluted in 30 µL of water. Samples were then tagged with ONT sequencing adaptors according to the modified one-pot ligation protocol^29^. Up to 24 samples were pooled prior to being run on the appropriate flow cell (FLO-MIN106) using the 72 h run script.

### Processing raw ONT data

Data were base-called in real time using the Oxford Nanopore software package Guppy 3.2.6. Sequencing data were then processed using the ARTIC bioinformatics pipeline (https://github.com/artic-network/artic-ncov2019), with a few modifications. Briefly, we have modified the ARTIC pipeline so that it demultiplexes raw fastq files using qcat as each fastq file is generated by the GridION (https://github.com/nanoporetech/qcat). Once a barcode reaches 100,000 reads, it will trigger the rest of the ARTIC bioinformatics workflow which will map to the severe acute respiratory syndrome coronavirus 2 isolate Wuhan-Hu-1 reference (Genbank: MN908947.3) using minimap2. This alignment will then be used to generate consensus sequences and variant calls using medaka (https://github.com/nanoporetech/medaka). The entire ONT analysis pipeline is available at the GitHub repository accompanying this manuscript^61^.

### Phylogenetic analysis

All 247 available full-length sequences from Dane and Milwaukee county through 26 April 2020 were used for phylogenetic analysis using the tools implemented in Nextstrain custom builds (https://github.com/nextstrain/ncov)^4,62^. Time-resolved and divergence phylogenetic trees were built using the standard Nextstrain tools and scripts^4,62^. We used custom python scripts to filter and clean metadata.

An additional subsampled global phylogeny using all available sequences in GISAID as of 21 June 2020 were input into the Nextstrain pipeline. A custom “Wisconsin” profile was made to create a Wisconsin-centric subsampled build to include representative sequences. To reduce combat bias, we defined representative sequences as 20 sequences from each US state, and 30 sequences from each country, per month per year. This subsampled global build includes 5377 sequences or roughly 11% of the total sequences in GISAID as of 21 June 2020. All available Wisconsin sequences available on GISAID by 21 June 2020 were incorporated into the subsampled global tree. All of the Wisconsin sequences included in this study are listed in the include.txt file to ensure they were represented in the global phylogeny. The scripts and output are available at the GitHub repository accompanying this manuscript^61^.

### Estimating the number of introductions

To estimate the number of unique introductions into Dane and Milwaukee county, we first identified the closest phylogenetic neighbor of each Dane and Milwaukee county sequence in the global (as of 14 June 2020) SARS-CoV-2 phylogenetic tree generated by Dr. Robert Lanfear at the Australian National University. These trees are generated using MAFFT^63^ and FastTree^64^ and are available at https://github.com/roblanf/sarscov2phylo/. To identify the closest phylogenetic neighbors we first pruned all tips from this tree with ambiguous collection dates (e.g. those given only by month and year as opposed to day, month, and year) and all tips which were excluded from our global alignment using the Nextstrain exclusion criteria (minimum length of 27,000 nucleotides, sequences listed in the “exclude” configuration file, sequences with admin division listed as “USA”) using BioPython. Next, we identified the parent node of each Dane and Milwaukee county tip and then identified the closest phylogenetic neighbor as the other descendant from this node. Aligned neighbor sequences, if not already present, were added to the downsampled alignment described above, resulting in an alignment of 5417 sequences. We inferred a maximum likelihood phylogeny of this alignment using IQ-TREE^65^ with 1000 Ultrafast bootstrap replicates^66^ using the flags -nt 4 -ninit 10 -me 0.05 -bb 1000 -wbtl -czb. The tree was rooted at Wuhan/WH01/2019 and TreeTime^62^ was used to prune tips from the maximum likelihood tree which did not follow a molecular clock (n_iqd = 4), create a time aligned tree (infer_gtr=True max_iter=2 branch_length_mode = ’auto’ resolve_polytomies=False time_marginal = ’assign’ vary_rate=0.0004 fixed_clock_rate=0.0008^67^), and infer the geographical locations (Dane county, Milwaukee county, U.S. States, county) of internal nodes (sampling_bias_correction=2.5 to account for undersampling).

To estimate the number of introductions into Dane county and Milwaukee county, this procedure was repeated on 100 of the bootstrap replicate trees. Using each of the 100 bootstrap replicate trees, we identified the earliest node in the path between the root of the tree and each Wisconsin (Dane county, Milwaukee county, and other Wisconsin) tip which was assigned to Wisconsin using the ancestral state reconstruction. Introduction into Wisconsin was assumed to occur mid-way between the earliest Wisconsin node and its parent. The time of introduction was evaluated using the mean estimate as well as the lower and upper limits of the timing for each node. Thus, each bootstrap replicate contributes three lines to the plots shown in Fig. 3b, c. As we do not know whether Wisconsin samples included in the tree from other studies are from Dane or Milwaukee county (or elsewhere in Wisconsin), our estimates for the timing of introduction into each county represent the timing of introduction of that lineage into Wisconsin generally. We conservatively attribute any Dane or Milwaukee county tips or lineages directly descending from a polytomic internal node to a single importation event.

To account for biased sampling within Dane and Milwaukee county, we conducted a rarefaction analysis. This was done using the time aligned maximum likelihood tree described above. *N* (20−240, in increments of 20) sequences were randomly sampled from the set of Dane and Milwaukee county sequences and all nonsampled Dane and Milwaukee county sequences were pruned from the tree prior to ancestral state reconstruction and estimation of the number of introductions as described above. Ten replicates for each *N* were conducted.

Code to replicate this analysis is available at the GitHub accompanying this manuscript^61^. Results were visualized using Matplotlib^68^, Seaborn (https://github.com/mwaskom/seaborn), and Baltic (https://github.com/evogytis/baltic).

### Phylodynamic analysis

Bayesian phylogenetic inference and dynamic modeling were performed with BEAST2 software (v2.6.2)^69^ and the PhyDyn package (v1.3.6)^16^. The phylodynamic analysis infers SARS-CoV-2 phylogenies of sequences within a region of interest and exogenous sequences representing the global phylogeny, and uses tree topology to inform an SEIJR compartmental model. For the Bayesian phylogenetic analysis, an HKY substitution model (gamma count = 4; *Κ* lognormal prior (*μ* = 1, *S* = 1.25)) and a strict molecular clock (uniform prior 0.0005−0.005 substitution/site/year) were used. To select the exogenous sequences, a maximum-likelihood global phylogeny was generated with IQTree and randomly downsampled in a time-stratified manner by collection week. Closest cophenetic neighbors for each of the Wisconsin sequences were additionally included, if not present already. Only sequences with coverage of the entire coding region and less than 1% of *N* base calls were used. For the Dane county analyses, 107 local and 107 exogenous SARS-CoV-2 sequences were used. For the Milwaukee county analyses, 117 local and 129 exogenous SARS-CoV-2 sequences were used.

The SEIJR model dynamics are defined by the following ordinary differential equations:

1 $${\mathrm{d}}S/{\mathrm{d}}t = - (\beta {{I}}(t) + \beta \tau {{J}}(t))\frac{{S(t)}}{{S(t) + E(t) + I(t) + J(t) + R(t)}},$$

2 $${\mathrm{d}}E/{\mathrm{d}}t = (\beta I(t) + \beta \tau J(t))\frac{{S(t)}}{{S(t) + E(t) + I(t) + J(t) + R(t)}} - \gamma _0E(t),$$

3 $${\mathrm{d}}I/{\mathrm{d}}t = \gamma _0(1 - p_h)E(t) - \gamma _1J(t),$$

4 $${\mathrm{d}}J/{\mathrm{d}}t = \gamma _0p_hE(t) - \gamma _1J(t),$$

5 $${\mathrm{d}}R/{\mathrm{d}}t = \gamma _1(E(t) + J(t)),$$

The dynamics of the exogenous compartment is defined by:

6 $${\mathrm{d}}Y/{\mathrm{d}}t = (\beta _{{\mathrm{{exog}}}} - \gamma _{{\mathrm{{exog}}}})Y(t).$$

During phylodynamic model fitting, *β*, *β*_exog_, and *α* are estimated. Estimated *R*_0_ was derived from *β* as follows.

7 $$R_0 = (\beta (1 - p_h) + \beta (\tau p_h))/\gamma _1.$$

The epidemic growth rate of the phylodynamic model is governed by the system of differential equations, and can thus be informed by SARS-CoV-2-specific transmission parameters. The SEIJR model includes a “high transmission” compartment (*J*) that accounts for heterogeneous transmission due to superspreading, an important component of SARS-CoV-2 epidemiology^11,70–72^. Published empirical estimates informed parameterization of superspreading and other epidemiological parameters. The mean duration of latent (1/*γ*_0_) and infectious periods (1/*γ*_1_) was 3 and 5.5 days, respectively^73^. Likewise, the mean duration of infection for the exogenous compartment (1/*γ*_exog_) was fixed at 8.5 days. To model low, medium, and high transmission heterogeneity, the proportion of infectious individuals in the J compartment (*p*_*h*_) and their transmission rate multiplier (*τ*) were set to 0.2 and 16, 0.1 and 36, or 0.05 and 76, respectively. These *p*_*h*_ and *τ* settings result in 20, 10, or 5% of individuals contributing 80% of total infections. The initial size of the S compartment was fixed at 5 × 105 for Dane county and 9.5 × 105 for Milwaukee county. To account for changes in epidemic dynamics after the Executive Orders, a 25% reduction in importation/exportation of sequences was applied at a 25 March breakpoint, per observed reductions in Google mobility indices for individuals in Wisconsin^74^. Additionally, the estimated *R*_0_ after 25 March was allowed to vary from the pre-intervention *R*_0_ proportionally by a modifier variable, *α*.

Each analysis was run in duplicate for at least 3 million states in BEAST2. Parameter traces were visually inspected for adequate mixing and convergence in Tracer (v1.7.1). Log files from duplicate runs were merged with LogCombiner and 10% burn-in applied. Similarly, trajectory files from duplicate runs were merged with an in-house R script and 10% burn-in applied. BEAST2 XML files and scripts for exogenous sequence selection and phylodynamic data analysis/visualization are provided in the GitHub repository listed below.

### Reporting summary

Further information on research design is available in the Nature Research Reporting Summary linked to this article.

## Supplementary information

Supplementary Information

Reporting Summary

## Data Availability

Source data have been deposited in the Sequence Read Archive (SRA) under bioproject PRJNA614504. The consensus genome sequences for national and international genomes are available from GISAID (www.gisaid.org; see Supplementary Table 3). Source data, derived data, analysis pipelines, and figures have been made available for replication of these results at a publicly accessible GitHub repository^61^. For the county-level case data and demographic data presented in Fig. 1, we obtained a county-level map of Wisconsin from the State Cartographer’s Office (https://www.sco.wisc.edu/maps/wisconsin-outline/). We obtained Wisconsin county-level COVID-19 cumulative case data from the Wisconsin Department of Health Services COVID-19 dashboard (https://data.dhsgis.wi.gov/datasets/covid-19-historical-data-table/, https://cityofmadison.maps.arcgis.com/apps/opsdashboard/index.html#/e22f5ba4f1f94e0bb0b9529dc82db6a3, and https://county.milwaukee.gov/EN/COVID-19). All Dane and Milwaukee county demographic data came from the Wisconsin Department of Health Services Data & Statistics (https://www.dhs.wisconsin.gov/stats) or the U.S. Census Bureau QuickFacts table (https://www.census.gov/quickfacts/fact/table/). Source data are provided with this paper.

## Source data

Source Data

## Code availability

Code to replicate these analyses are available at https://github.com/gagekmoreno/SARS-CoV-2-in-Southern-Wisconsin. Code to process sequencing data was made available by ARTIC bioinformatics pipeline (https://github.com/artic-network/artic-ncov2019) and uses Minimap2 v2.17 (https://github.com/lh3/minimap2) and medaka v1.03 (https://github.com/nanoporetech/medaka). Phylogenetic trees were built using Nextstrain tools and clade nomenclature (https://github.com/nextstrain/ncov). The global subsampled trees were generated using MAFFT v7.464 (https://mafft.cbrc.jp/alignment/software/), FastTree v2.1.10^64^, and IQ-TREE v1.5.5 (http://www.iqtree.org), and are available at http://github.com/roblanf/sarscov2phylo/. Results were visualized using Matplotlib 3.3.2 (https://matplotlib.org), Seaborn v0.10.0 (https://github.com/mwaskom/seaborn), and Baltic v0.1.0 (https://github.com/evogytis/baltic). Bayesian phylogenetic inference and dynamic modeling were performed with BEAST2 software v2.6.2^69^ and the PhyDyn package v1.3.6^16^. Parameter traces were visualized in Tracer v1.7.1 (http://tree.bio.ed.ac.uk/software/tracer/).
