# Supplementary material for: Revealing fine-scale spatiotemporal differences in SARS-CoV-2 introduction and spread
Source: Nat Commun. 2020 Nov 3;11:5558. doi: 10.1038/s41467-020-19346-z (PMC7609670; doi:10.1038/s41467-020-19346-z)
Supplement: Supplementary file 1 — Supplementary Information [file 41467_2020_19346_MOESM1_ESM.pdf]

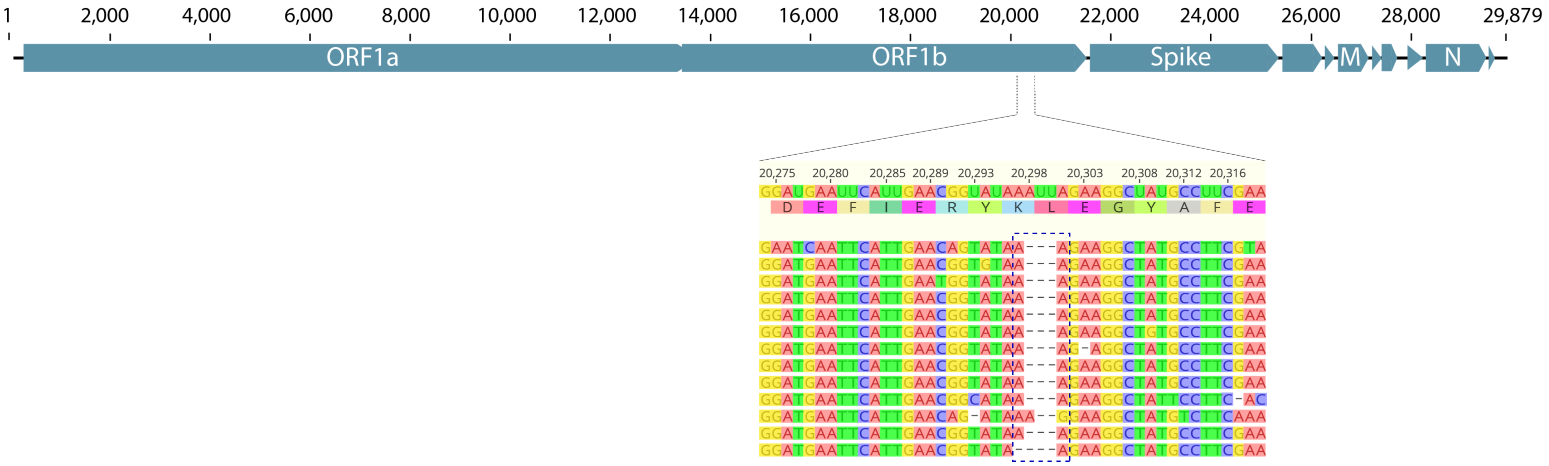

Supplementary Figure 1. Diagnostic deletion in the index Dane County sample
Consensus-level deletion identified in the Dane County index sample. Zoomed in panel shows nucleotide and amino acid identities of the in-frame deletion.

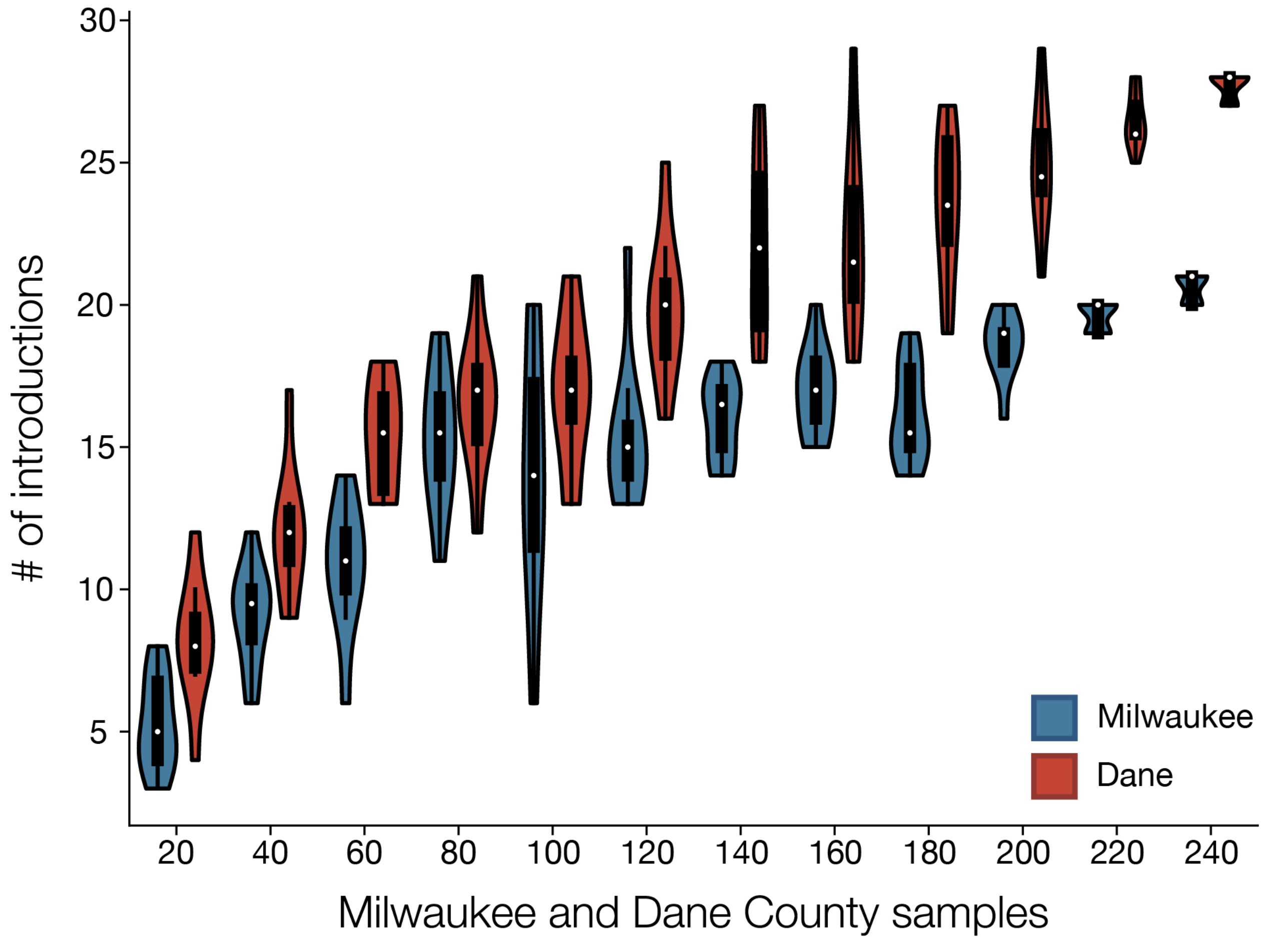

Supplementary Figure 2. Sampling sensitivity of estimates for the number of introductions into Dane and Milwaukee Counties. Estimates of the number of introductions into Dane and Milwaukee Counties using a time aligned maximum likelihood phylogeny. N sequences (x-axis) were randomly sampled from the available Dane and Milwaukee County samples and the remaining were pruned from the tree. Ten replicates of each N were conducted and the number of introductions (y-axis) was estimated for each. For each number of Milwaukee and Dane County samples, the kernel density estimate of the underlying distribution of the number of introductions is shown. The bandwidth is calculated using Scott's rule and the distribution is only shown over the range of observed values 76 Internal boxes (black) represent the interquartile range (IQR, 25th to 75th percentile) of the distribution and the median is represented by a white dot. Whiskers extend to the range of data that are not more than 1.5 times the IQR above the upper or below the lower quartile. Source data to replicate this figure can be found in the Source Data file.

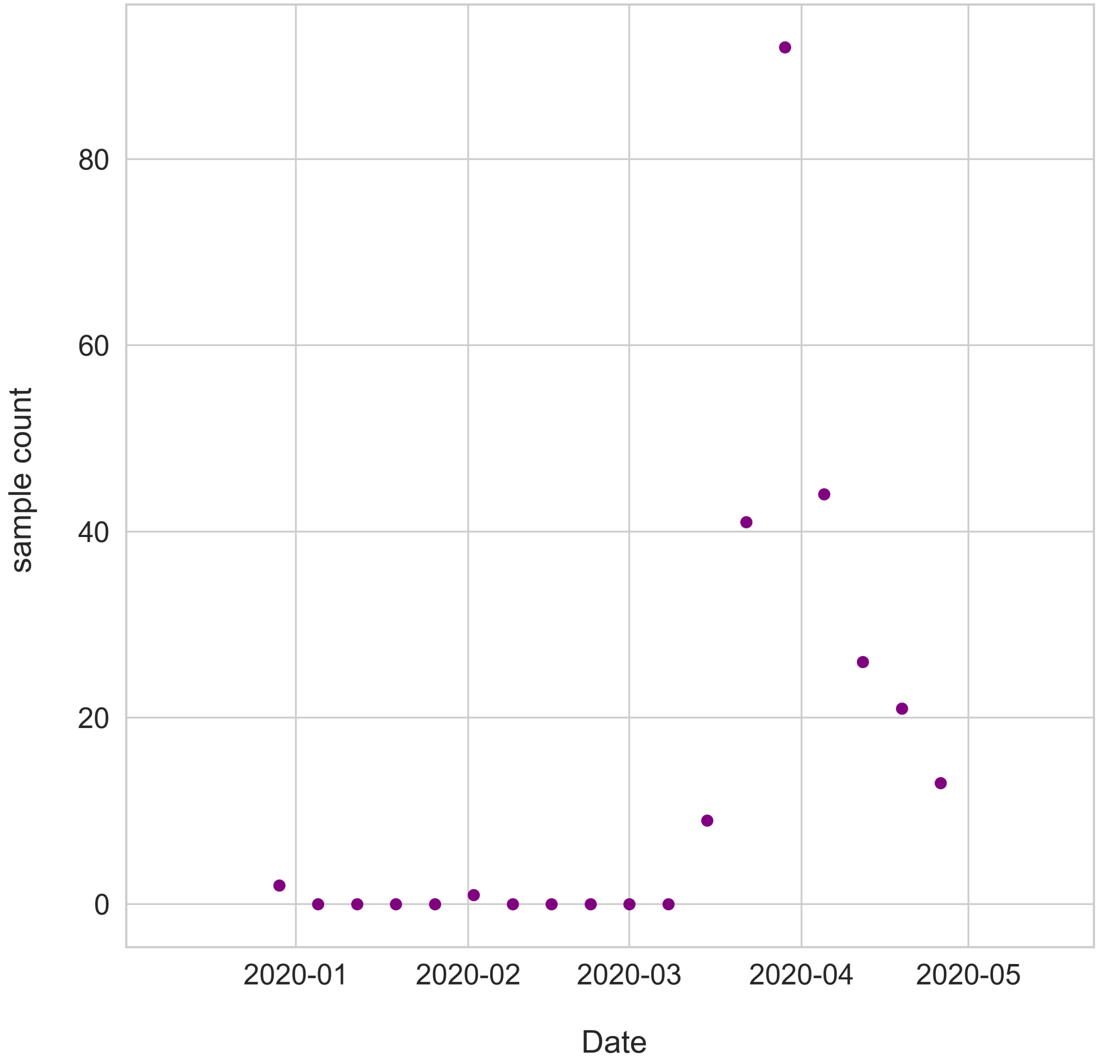

Supplementary Figure 3. Temporal distribution of SARS-CoV-2 samples. The total count of samples collected (y-axis) during 1-week intervals (x-axis) from the first documented Wisconsin case through 18 April, 2020. Weeks with no confirmed cases within the defined study period are also shown here as indicated by data points at y=0. Source data to replicate this figure can be found in the Source Data file. Code to recreate this figure can be found in the GitHub repository – `data_raw/supplementary_figure_3.ipynb`.

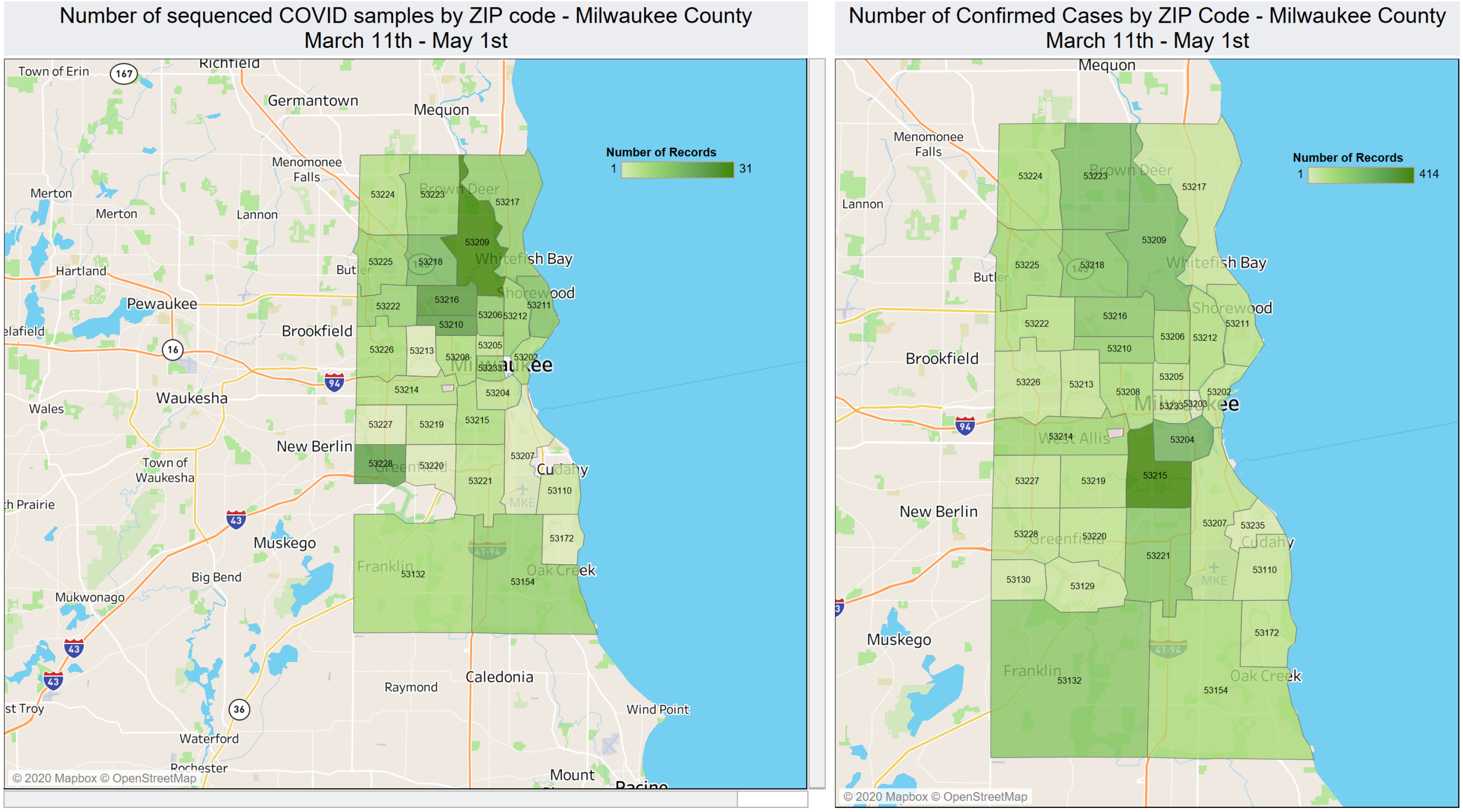

Supplementary Figure 4. Geographic distribution of Milwaukee County sequences (left) compared to test positive cases (right) by zip code. Case count data were collated using the Wisconsin Electronic Disease Surveillance System (WEDSS) and data were sorted and plotted using Tableau (https://www.tableau.com/) to create counts and color gradients by ZIP code.

Division	Number of sequences	Number of positive SARS-CoV-2 cases	Percent sequenced (%)
England	23212	265,849	8.73
Wales	5425	17389	31.2
Scotland	5121	18847	27.17
Washington	3957	55600	7.12
California	2189	500600	0.44
Victoria	1522	13469	11.3
New York	1505	411600	0.37
Michigan	1069	86200	1.24
Wisconsin	963	52900	1.82
Louisiana	786	116300	0.68
Singapore	770	54555	1.41
Denmark	735	14306	5.14
Northern Ireland	717	6049	11.85
Netherlands	692	56381	1.23
Utah	688	32400	2.12
New South Wales	656	3832	17.12
Portugal	642	52061	1.23
British Columbia	604	3834	15.75
Reykjavik	601	1932	31.11
Florida	560	469800	0.12
Basque Country	559	15634	3.58
Minnesota	545	54300	1
Virginia	522	89900	0.58
Gujarat	447	66777	0.67
Massachusetts	418	117200	0.36
Dane County - April 26th	122	405	30.12
Milwaukee County - April 26th	125	2629	4.75

Supplemental Table 1. SARS-CoV-2 sequencing depth per admin division. Admin division is defined as the state or country. Percent sequenced (%) is calculated as the number of consensus sequences over the number of documented positive SARS-CoV-2 cases across a variety of geographic locations as of July 31, 2020. The number of sequences per geographic location were obtained on GISAID by downloading the NextMeta file and filtering on the 'Admin Division' search field. We compared the sequencing depth to the sequencing depth obtained by our study (indicated by '- April 26th') to highlight that we are one of the top 25 deepest sequenced divisions.

Location	Number of sequences	Number of positive SARS-CoV-2 cases	Percent sequenced (%)
Yakima County	1704	9971	17.09
San Diego	702	29577	2.37
Sydney	543	3809	14.26
East Baton Rouge Parish	424	11263	3.76
Manhattan	419	222522	0.19
Brooklyn	373	61948	0.6
Valencia	343	12999	2.64
Dane County	334	4145	8.06
King County	322	15946	2.02
Greater Houston Area	320	80914	0.4
Santa Clara County	313	11128	2.81
Donostia-San Sebastian & Vitoria-Gasteiz	535	15634	3.42
Wuhan	257	68138	0.38
Nassau County	202	43482	0.46
Snohomish County	187	6033	3.1
Orange County	184	38131	0.48
Munich	181	51068	0.35
Queens	175	67598	0.26
South Yorkshire	171	9610	1.78
San Francisco	163	7231	2.25
Pierce County	159	5851	2.72
Milwaukee County	153	19332	0.79
Los Angeles County	146	198355	0.07
Hyderabad	144	73050	0.2
Dane County - April 26th	122	405	30.12
Milwaukee County - April 26th	125	2629	4.75

Supplemental Table 2. SARS-CoV-2 sequencing depth per location. Percent sequenced (%) is calculated as the number of consensus sequences over the number of documented positive SARS-CoV-2 cases across a variety of geographic locations. The number of sequences per geographic location were obtained on GISAID by downloading the NextMeta file and filtering on the 'Location' search field. We compared the sequencing depth to the sequencing depth obtained by our study (indicated by '- April 26th') to highlight that we are one of the top 25 deepest sequenced locations.

strain	gisaid_epi_isl	genbank_accession	sra_acession	srr_run_number	biosample_id	bioproject	date	region	country	division	location	region_exposure	country_exposure	division_exposure	segment	length	title	paper_url	date_submitted	N1_Ct value	N2_Ct value
USA/WI1/2020	EPI_ISL_408670	MT039887	SRX7777165	SRR11140745	SAMN14154204	PRJNA607948	2020-1-31	North America	USA	Wisconsin	Dane county	Asia	China	Hubei	genome	29879	First 12 patients with coronavirus disease 2019 (COVID-19) in the United States	https://dx.doi.org/10.1101/2020.03.09.20032896	2020-2-10	-	-
USA/WI-UW-02/2020	EPI_ISL_416489	-	SRX7972381	SRR11393278	SAMN14428237	PRJNA614504	2020-3-15	North America	USA	Wisconsin	Dane county	North America	USA	Wisconsin	genome	29782	?	?	2020-3-22	14.75	13.53
USA/WI-UW-04/2020	EPI_ISL_416491	-	SRX7972383	SRR11393276	SAMN14428239	PRJNA614504	2020-3-15	North America	USA	Wisconsin	Dane county	North America	USA	Wisconsin	genome	29843	?	?	2020-3-22	24.93	25.4
USA/WI-UW-05/2020	EPI_ISL_416492	-	SRX7972384	SRR11393275	SAMN14428240	PRJNA614504	2020-3-15	North America	USA	Wisconsin	Dane county	North America	USA	Wisconsin	genome	29855	?	?	2020-3-22	24.71	25.03
USA/WI-UW-03/2020	EPI_ISL_416523	-	SRX7972382	SRR11393277	SAMN14428238	PRJNA614504	2020-3-14	North America	USA	Wisconsin	Dane county	North America	USA	Wisconsin	genome	29870	?	?	2020-3-22	15.66	15
USA/WI-UW-06/2020	EPI_ISL_417200	-	SRX7988797	SRR11410125	SAMN14443954	PRJNA614504	2020-3-21	North America	USA	Wisconsin	Dane county	North America	USA	Wisconsin	genome	29844	?	?	2020-3-24	26.53	27.29
USA/WI-UW-07/2020	EPI_ISL_417201	-	SRX7988798	SRR11410124	SAMN14443955	PRJNA614504	2020-3-21	North America	USA	Wisconsin	Dane county	North America	USA	Wisconsin	genome	29865	?	?	2020-3-24	16.28	16.49
USA/WI-UW-08/2020	EPI_ISL_417202	-	SRX7988799	SRR11410123	SAMN14443956	PRJNA614504	2020-3-21	North America	USA	Wisconsin	Dane county	North America	USA	Wisconsin	genome	29843	?	?	2020-3-24	17.35	18.03
USA/WI-UW-09/2020	EPI_ISL_417203	-	SRX7988800	SRR11410122	SAMN14443957	PRJNA614504	2020-3-21	North America	USA	Wisconsin	Dane county	North America	USA	Wisconsin	genome	29843	?	?	2020-3-24	22.46	23.15
USA/WI-UW-10/2020	EPI_ISL_417204	-	SRX7988801	SRR11410121	SAMN14443958	PRJNA614504	2020-3-21	North America	USA	Wisconsin	Dane county	North America	USA	Wisconsin	genome	29841	?	?	2020-3-24	24.17	24.51
USA/WI-UW-25/2020	EPI_ISL_421283	-	SRX8079441	SRR11507378	SAMN14555931	PRJNA614504	2020-3-23	North America	USA	Wisconsin	Dane county	North America	USA	Wisconsin	genome	29782	?	?	2020-4-2	14.18	14.34
USA/WI-UW-26/2020	EPI_ISL_421284	-	SRX8079442	SRR11507377	SAMN14555932	PRJNA614504	2020-3-24	North America	USA	Wisconsin	Dane county	North America	USA	Wisconsin	genome	29611	?	?	2020-4-2	27.55	29.14
USA/WI-UW-27/2020	EPI_ISL_421285	-	SRX8079453	SRR11507366	SAMN14555933	PRJNA614504	2020-3-25	North America	USA	Wisconsin	Dane county	North America	USA	Wisconsin	genome	29782	?	?	2020-4-2	30.36	31.89
USA/WI-UW-28/2020	EPI_ISL_421286	-	SRX8079464	SRR11507355	SAMN14555934	PRJNA614504	2020-3-25	North America	USA	Wisconsin	Dane county	North America	USA	Wisconsin	genome	29782	?	?	2020-4-2	27.7	29.21
USA/WI-UW-29/2020	EPI_ISL_421287	-	SRX8079475	SRR11507344	SAMN14555935	PRJNA614504	2020-3-24	North America	USA	Wisconsin	Dane county	North America	USA	Wisconsin	genome	29782	?	?	2020-4-2	16.14	16.05
USA/WI-UW-31/2020	EPI_ISL_421289	-	SRX8079497	SRR11507322	SAMN14555937	PRJNA614504	2020-3-25	North America	USA	Wisconsin	Dane county	North America	USA	Wisconsin	genome	29782	?	?	2020-4-2	15.46	15.69
USA/WI-UW-32/2020	EPI_ISL_421290	-	SRX8079498	SRR11507321	SAMN14555938	PRJNA614504	2020-3-24	North America	USA	Wisconsin	Dane county	North America	USA	Wisconsin	genome	29782	?	?	2020-4-2	24.27	24.83
USA/WI-UW-33/2020	EPI_ISL_421291	-	SRX8079499	SRR11507320	SAMN14555939	PRJNA614504	2020-3-23	North America	USA	Wisconsin	Dane county	North America	USA	Wisconsin	genome	29782	?	?	2020-4-2	23.18	23.94
USA/WI-UW-34/2020	EPI_ISL_421292	-	SRX8079500	SRR11507319	SAMN14555940	PRJNA614504	2020-3-26	North America	USA	Wisconsin	Dane county	North America	USA	Wisconsin	genome	29782	?	?	2020-4-2	27.81	29.4
USA/WI-UW-35/2020	EPI_ISL_421293	-	SRX8079443	SRR11507376	SAMN14555941	PRJNA614504	2020-3-24	North America	USA	Wisconsin	Dane county	North America	USA	Wisconsin	genome	29782	?	?	2020-4-2	20.93	21.61
USA/WI-UW-37/2020	EPI_ISL_421295	-	SRX8079445	SRR11507374	SAMN14555943	PRJNA614504	2020-3-22	North America	USA	Wisconsin	Dane county	North America	USA	Wisconsin	genome	29782	?	?	2020-4-2	23.69	24.79

USA/WI-UW-38/2020	EPI_ISL_421296	-	SRX8079446	SRR11507373	SAMN14555944	PRJNA614504	2020-3-25	North America	USA	Wisconsin	Dane county	North America	USA	Wisconsin	genome	29782	?	?	2020-4-2	27.98	29.23
USA/WI-UW-39/2020	EPI_ISL_421297	-	SRX8079447	SRR11507372	SAMN14555945	PRJNA614504	2020-3-22	North America	USA	Wisconsin	Dane county	North America	USA	Wisconsin	genome	29782	?	?	2020-4-2	18.2	20.06
USA/WI-UW-40/2020	EPI_ISL_421298	-	SRX8079448	SRR11507371	SAMN14555946	PRJNA614504	2020-3-24	North America	USA	Wisconsin	Dane county	North America	USA	Wisconsin	genome	29782	?	?	2020-4-2	23.52	24.46
USA/WI-UW-41/2020	EPI_ISL_421299	-	SRX8079449	SRR11507370	SAMN14555947	PRJNA614504	2020-3-25	North America	USA	Wisconsin	Dane county	North America	USA	Wisconsin	genome	29782	?	?	2020-4-2	24.32	25.31
USA/WI-UW-42/2020	EPI_ISL_421300	-	SRX8079450	SRR11507369	SAMN14555948	PRJNA614504	2020-3-18	North America	USA	Wisconsin	Dane county	North America	USA	Wisconsin	genome	29782	?	?	2020-4-2	16.29	16.82
USA/WI-UW-43/2020	EPI_ISL_421301	-	SRX8079451	SRR11507368	SAMN14555949	PRJNA614504	2020-3-19	North America	USA	Wisconsin	Dane county	North America	USA	Wisconsin	genome	29782	?	?	2020-4-2	14.76	15.72
USA/WI-UW-44/2020	EPI_ISL_421302	-	SRX8079452	SRR11507367	SAMN14555950	PRJNA614504	2020-3-17	North America	USA	Wisconsin	Dane county	North America	USA	Wisconsin	genome	29782	?	?	2020-4-2	17.79	18.3
USA/WI-UW-45/2020	EPI_ISL_421303	-	SRX8079454	SRR11507365	SAMN14555951	PRJNA614504	2020-3-22	North America	USA	Wisconsin	Dane county	North America	USA	Wisconsin	genome	29782	?	?	2020-4-2	20.22	20.7
USA/WI-UW-46/2020	EPI_ISL_421304	-	SRX8079455	SRR11507364	SAMN14555952	PRJNA614504	2020-3-22	North America	USA	Wisconsin	Dane county	North America	USA	Wisconsin	genome	29782	?	?	2020-4-2	27.13	27.94
USA/WI-UW-48/2020	EPI_ISL_421306	-	SRX8079457	SRR11507362	SAMN14555954	PRJNA614504	2020-3-25	North America	USA	Wisconsin	Dane county	North America	USA	Wisconsin	genome	29782	?	?	2020-4-2	23.02	23.79
USA/WI-UW-49/2020	EPI_ISL_421307	-	SRX8079458	SRR11507361	SAMN14555955	PRJNA614504	2020-3-25	North America	USA	Wisconsin	Dane county	North America	USA	Wisconsin	genome	29782	?	?	2020-4-2	25.7	26.9
USA/WI-UW-50/2020	EPI_ISL_421308	-	SRX8079459	SRR11507360	SAMN14555956	PRJNA614504	2020-3-25	North America	USA	Wisconsin	Green County	North America	USA	Wisconsin	genome	29782	?	?	2020-4-2	19.13	19.78
USA/WI-UW-51/2020	EPI_ISL_421309	-	SRX8079460	SRR11507359	SAMN14555957	PRJNA614504	2020-3-20	North America	USA	Wisconsin	Dane county	North America	USA	Wisconsin	genome	29782	?	?	2020-4-2	17.11	17.3
USA/WI-UW-52/2020	EPI_ISL_421310	-	SRX8079461	SRR11507358	SAMN14555958	PRJNA614504	2020-3-18	North America	USA	Wisconsin	Dane county	North America	USA	Wisconsin	genome	29782	?	?	2020-4-2	15.98	16.57
USA/WI-UW-53/2020	EPI_ISL_421311	-	SRX8079462	SRR11507357	SAMN14555959	PRJNA614504	2020-3-18	North America	USA	Wisconsin	Dane county	North America	USA	Wisconsin	genome	29782	?	?	2020-4-2	14.46	14.85
USA/WI-UW-54/2020	EPI_ISL_421312	-	SRX8079463	SRR11507356	SAMN14555960	PRJNA614504	2020-3-20	North America	USA	Wisconsin	Dane county	North America	USA	Wisconsin	genome	29782	?	?	2020-4-2	16.12	16.18
USA/WI-UW-55/2020	EPI_ISL_421313	-	SRX8079465	SRR11507354	SAMN14555961	PRJNA614504	2020-3-23	North America	USA	Wisconsin	Dane county	North America	USA	Wisconsin	genome	29782	?	?	2020-4-2	16.85	17.81
USA/WI-UW-56/2020	EPI_ISL_421314	-	SRX8079466	SRR11507353	SAMN14555962	PRJNA614504	2020-3-18	North America	USA	Wisconsin	Dane county	North America	USA	Wisconsin	genome	29782	?	?	2020-4-2	24.33	24.95
USA/WI-UW-58/2020	EPI_ISL_421316	-	SRX8079468	SRR11507351	SAMN14555964	PRJNA614504	2020-3-23	North America	USA	Wisconsin	Dane county	North America	USA	Wisconsin	genome	29782	?	?	2020-4-2	16.63	16.64
USA/WI-UW-59/2020	EPI_ISL_421317	-	SRX8079469	SRR11507350	SAMN14555965	PRJNA614504	2020-3-24	North America	USA	Wisconsin	Dane county	North America	USA	Wisconsin	genome	29782	?	?	2020-4-2	23.83	24.54
USA/WI-UW-60/2020	EPI_ISL_421318	-	SRX8079470	SRR11507349	SAMN14555966	PRJNA614504	2020-3-19	North America	USA	Wisconsin	Dane county	North America	USA	Wisconsin	genome	29782	?	?	2020-4-2	16.48	16.48
USA/WI-UW-61/2020	EPI_ISL_421319	-	SRX8079471	SRR11507348	SAMN14555967	PRJNA614504	2020-3-23	North America	USA	Wisconsin	Dane county	North America	USA	Wisconsin	genome	29782	?	?	2020-4-2	23.28	24.11
USA/WI-UW-62/2020	EPI_ISL_421320	-	SRX8079472	SRR11507347	SAMN14555968	PRJNA614504	2020-3-25	North America	USA	Wisconsin	Dane county	North America	USA	Wisconsin	genome	29782	?	?	2020-4-2	26.29	27.46
USA/WI-UW-63/2020	EPI_ISL_421321	-	SRX8079473	SRR11507346	SAMN14555969	PRJNA614504	2020-3-24	North America	USA	Wisconsin	Dane county	North America	USA	Wisconsin	genome	29782	?	?	2020-4-2	24.54	25.21
USA/WI-UW-64/2020	EPI_ISL_421322	-	SRX8079474	SRR11507345	SAMN14555970	PRJNA614504	2020-3-24	North America	USA	Wisconsin	Dane county	North America	USA	Wisconsin	genome	29782	?	?	2020-4-2	17.96	17.57

USA/WI-UW-65/2020	EPI_ISL_421323	-	SRX8079476	SRR11507343	SAMN14555971	PRJNA614504	2020-3-22	North America	USA	Wisconsin	Dane county	North America	USA	Wisconsin	genome	29782	?	?	2020-4-2	15.54	15.33
USA/WI-UW-66/2020	EPI_ISL_421324	-	SRX8079477	SRR11507342	SAMN14555972	PRJNA614504	2020-3-24	North America	USA	Wisconsin	Dane county	North America	USA	Wisconsin	genome	29782	?	?	2020-4-2	26.52	27.61
USA/WI-UW-67/2020	EPI_ISL_421325	-	SRX8079478	SRR11507341	SAMN14555973	PRJNA614504	2020-3-25	North America	USA	Wisconsin	Dane county	North America	USA	Wisconsin	genome	29782	?	?	2020-4-2	25.62	27.01
USA/WI-UW-68/2020	EPI_ISL_421326	-	SRX8079479	SRR11507340	SAMN14555974	PRJNA614504	2020-3-24	North America	USA	Wisconsin	Dane county	North America	USA	Wisconsin	genome	29782	?	?	2020-4-2	15.96	16.13
USA/WI-UW-69/2020	EPI_ISL_421327	-	SRX8079480	SRR11507339	SAMN14555975	PRJNA614504	2020-3-19	North America	USA	Wisconsin	Dane county	North America	USA	Wisconsin	genome	29782	?	?	2020-4-2	15.83	16.07
USA/WI-UW-70/2020	EPI_ISL_421328	-	SRX8079481	SRR11507338	SAMN14555976	PRJNA614504	2020-3-19	North America	USA	Wisconsin	Dane county	North America	USA	Wisconsin	genome	29782	?	?	2020-4-2	20.12	20.77
USA/WI-UW-71/2020	EPI_ISL_421329	-	SRX8079482	SRR11507337	SAMN14555977	PRJNA614504	2020-3-24	North America	USA	Wisconsin	Dane county	North America	USA	Wisconsin	genome	29782	?	?	2020-4-2	18.93	18.64
USA/WI-UW-72/2020	EPI_ISL_421330	-	SRX8079483	SRR11507336	SAMN14555978	PRJNA614504	2020-3-25	North America	USA	Wisconsin	Dane county	North America	USA	Wisconsin	genome	29782	?	?	2020-4-2	20.95	21.64
USA/WI-UW-73/2020	EPI_ISL_421331	-	SRX8079484	SRR11507335	SAMN14555979	PRJNA614504	2020-3-24	North America	USA	Wisconsin	Dane county	North America	USA	Wisconsin	genome	29782	?	?	2020-4-2	23.69	24.93
USA/WI-UW-74/2020	EPI_ISL_421332	-	SRX8079485	SRR11507334	SAMN14555980	PRJNA614504	2020-3-20	North America	USA	Wisconsin	Dane county	North America	USA	Wisconsin	genome	29782	?	?	2020-4-2	14.19	14.36
USA/WI-UW-75/2020	EPI_ISL_421333	-	SRX8079487	SRR11507332	SAMN14555981	PRJNA614504	2020-3-26	North America	USA	Wisconsin	Dane county	North America	USA	Wisconsin	genome	29782	?	?	2020-4-2	29.8	31.35
USA/WI-UW-76/2020	EPI_ISL_421334	-	SRX8079488	SRR11507331	SAMN14555982	PRJNA614504	2020-3-22	North America	USA	Wisconsin	Dane county	North America	USA	Wisconsin	genome	29782	?	?	2020-4-2	17.49	17.59
USA/WI-UW-77/2020	EPI_ISL_421335	-	SRX8079489	SRR11507330	SAMN14555983	PRJNA614504	2020-3-19	North America	USA	Wisconsin	Dane county	North America	USA	Wisconsin	genome	29782	?	?	2020-4-2	20.19	20.65
USA/WI-UW-78/2020	EPI_ISL_421336	-	SRX8079490	SRR11507329	SAMN14555984	PRJNA614504	2020-3-24	North America	USA	Wisconsin	Dane county	North America	USA	Wisconsin	genome	29782	?	?	2020-4-2	25.99	26.68
USA/WI-UW-79/2020	EPI_ISL_421338	-	SRX8079491	SRR11507328	SAMN14555985	PRJNA614504	2020-3-23	North America	USA	Wisconsin	Dane county	North America	USA	Wisconsin	genome	29782	?	?	2020-4-2	25.04	25.84
USA/WI-UW-80/2020	EPI_ISL_421339	-	SRX8079492	SRR11507327	SAMN14555986	PRJNA614504	2020-3-24	North America	USA	Wisconsin	Dane county	North America	USA	Wisconsin	genome	29782	?	?	2020-4-2	21.56	22.09
USA/WI-UW-81/2020	EPI_ISL_421340	-	SRX8079493	SRR11507326	SAMN14555987	PRJNA614504	2020-3-25	North America	USA	Wisconsin	Dane county	North America	USA	Wisconsin	genome	29782	?	?	2020-4-2	21.88	23.08
USA/WI-UW-82/2020	EPI_ISL_421341	-	SRX8079494	SRR11507325	SAMN14555988	PRJNA614504	2020-3-25	North America	USA	Wisconsin	Dane county	North America	USA	Wisconsin	genome	29782	?	?	2020-4-2	28.13	29.11
USA/WI-UW-84/2020	EPI_ISL_421343	-	SRX8079496	SRR11507323	SAMN14555990	PRJNA614504	2020-3-24	North America	USA	Wisconsin	Dane county	North America	USA	Wisconsin	genome	29782	?	?	2020-4-2	23.12	23.82
USA/WI-UW-85/2020	EPI_ISL_425142	-	SRX8114902	SRR11544850	SAMN14596861	PRJNA614504	2020-4-2	North America	USA	Wisconsin	Dane county	North America	USA	Wisconsin	genome	29782	?	?	2020-4-12	24.4	-
USA/WI-UW-86/2020	EPI_ISL_425143	-	SRX8114903	SRR11544849	SAMN14596862	PRJNA614504	2020-4-2	North America	USA	Wisconsin	Dane county	North America	USA	Wisconsin	genome	29782	?	?	2020-4-12	22.1	-
USA/WI-UW-87/2020	EPI_ISL_425144	-	SRX8114914	SRR11544838	SAMN14596863	PRJNA614504	2020-4-2	North America	USA	Wisconsin	Dane county	North America	USA	Wisconsin	genome	29782	?	?	2020-4-12	22.1	-
USA/WI-UW-88/2020	EPI_ISL_425145	-	SRX8114925	SRR11544827	SAMN14596864	PRJNA614504	2020-4-5	North America	USA	Wisconsin	Dane county	North America	USA	Wisconsin	genome	29782	?	?	2020-4-12	25.29	25.93
USA/WI-UW-89/2020	EPI_ISL_425146	-	SRX8114931	SRR11544821	SAMN14596865	PRJNA614504	2020-3-31	North America	USA	Wisconsin	Dane county	North America	USA	Wisconsin	genome	29782	?	?	2020-4-12	21.63	22.3
USA/WI-UW-90/2020	EPI_ISL_425147	-	SRX8114932	SRR11544820	SAMN14596866	PRJNA614504	2020-4-3	North America	USA	Wisconsin	Dane county	North America	USA	Wisconsin	genome	29782	?	?	2020-4-12	30.7	-

USA/WI-UW-91/2020	EPI_ISL_425148	-	SRX8114933	SRR11544819	SAMN14596867	PRJNA614504	2020-4-1	North America	USA	Wisconsin	Dane county	North America	USA	Wisconsin	genome	29611	?	?	2020-4-12	24.6	-
USA/WI-UW-92/2020	EPI_ISL_425149	-	SRX8114934	SRR11544818	SAMN14596868	PRJNA614504	2020-3-30	North America	USA	Wisconsin	Dane county	North America	USA	Wisconsin	genome	29782	?	?	2020-4-12	18.8	19.7
USA/WI-UW-93/2020	EPI_ISL_425150	-	SRX8114935	SRR11544817	SAMN14596869	PRJNA614504	2020-4-4	North America	USA	Wisconsin	Dane county	North America	USA	Wisconsin	genome	29782	?	?	2020-4-12	25.7	-
USA/WI-UW-94/2020	EPI_ISL_425151	-	SRX8114936	SRR11544816	SAMN14596870	PRJNA614504	2020-3-30	North America	USA	Wisconsin	Dane county	North America	USA	Wisconsin	genome	29782	?	?	2020-4-12	28.8	-
USA/WI-UW-95/2020	EPI_ISL_425152	-	SRX8114904	SRR11544848	SAMN14596871	PRJNA614504	2020-3-30	North America	USA	Wisconsin	Dane county	North America	USA	Wisconsin	genome	29782	?	?	2020-4-12	21.2	-
USA/WI-UW-96/2020	EPI_ISL_425153	-	SRX8114905	SRR11544847	SAMN14596872	PRJNA614504	2020-4-1	North America	USA	Wisconsin	Dane county	North America	USA	Wisconsin	genome	29782	?	?	2020-4-12	17.33	18.05
USA/WI-UW-97/2020	EPI_ISL_425154	-	SRX8114906	SRR11544846	SAMN14596873	PRJNA614504	2020-3-30	North America	USA	Wisconsin	Dane county	North America	USA	Wisconsin	genome	29611	?	?	2020-4-12	27.3	-
USA/WI-UW-98/2020	EPI_ISL_425155	-	SRX8114907	SRR11544845	SAMN14596874	PRJNA614504	2020-3-31	North America	USA	Wisconsin	Dane county	North America	USA	Wisconsin	genome	29782	?	?	2020-4-12	24.7	-
USA/WI-UW-99/2020	EPI_ISL_425156	-	SRX8114908	SRR11544844	SAMN14596875	PRJNA614504	2020-4-2	North America	USA	Wisconsin	Dane county	North America	USA	Wisconsin	genome	29782	?	?	2020-4-12	18.8	-
USA/WI-UW-100/2020	EPI_ISL_425157	-	SRX8114909	SRR11544843	SAMN14596876	PRJNA614504	2020-4-3	North America	USA	Wisconsin	Dane county	North America	USA	Wisconsin	genome	29782	?	?	2020-4-12	26.5	-
USA/WI-UW-101/2020	EPI_ISL_425158	-	SRX8114910	SRR11544842	SAMN14596877	PRJNA614504	2020-3-31	North America	USA	Wisconsin	Dane county	North America	USA	Wisconsin	genome	29782	?	?	2020-4-12	16.9	-
USA/WI-UW-102/2020	EPI_ISL_425159	-	SRX8114911	SRR11544841	SAMN14596878	PRJNA614504	2020-3-30	North America	USA	Wisconsin	Dane county	North America	USA	Wisconsin	genome	29782	?	?	2020-4-12	22.21	22.99
USA/WI-UW-103/2020	EPI_ISL_425160	-	SRX8114912	SRR11544840	SAMN14596879	PRJNA614504	2020-4-3	North America	USA	Wisconsin	Dane county	North America	USA	Wisconsin	genome	29782	?	?	2020-4-12	23.6	-
USA/WI-UW-105/2020	EPI_ISL_425162	-	SRX8114915	SRR11544837	SAMN14596881	PRJNA614504	2020-3-30	North America	USA	Wisconsin	Dane county	North America	USA	Wisconsin	genome	29782	?	?	2020-4-12	25.21	26.49
USA/WI-UW-106/2020	EPI_ISL_425163	-	SRX8114916	SRR11544836	SAMN14596882	PRJNA614504	2020-3-30	North America	USA	Wisconsin	Dane county	North America	USA	Wisconsin	genome	29782	?	?	2020-4-12	17.83	18.58
USA/WI-UW-107/2020	EPI_ISL_425164	-	SRX8114917	SRR11544835	SAMN14596883	PRJNA614504	2020-3-31	North America	USA	Wisconsin	Dane county	North America	USA	Wisconsin	genome	29782	?	?	2020-4-12	24.2	-
USA/WI-UW-108/2020	EPI_ISL_425165	-	SRX8114918	SRR11544834	SAMN14596884	PRJNA614504	2020-3-30	North America	USA	Wisconsin	Dane county	North America	USA	Wisconsin	genome	29782	?	?	2020-4-12	25.06	26.02
USA/WI-UW-109/2020	EPI_ISL_425166	-	SRX8114919	SRR11544833	SAMN14596885	PRJNA614504	2020-3-30	North America	USA	Wisconsin	Dane county	North America	USA	Wisconsin	genome	29782	?	?	2020-4-12	31.01	32.18
USA/WI-UW-110/2020	EPI_ISL_425167	-	SRX8114920	SRR11544832	SAMN14596886	PRJNA614504	2020-3-31	North America	USA	Wisconsin	Dane county	North America	USA	Wisconsin	genome	29782	?	?	2020-4-12	22.2	-
USA/WI-UW-111/2020	EPI_ISL_425168	-	SRX8114921	SRR11544831	SAMN14596887	PRJNA614504	2020-3-31	North America	USA	Wisconsin	Dane county	North America	USA	Wisconsin	genome	29782	?	?	2020-4-12	25.61	26.29
USA/WI-UW-112/2020	EPI_ISL_425169	-	SRX8114922	SRR11544830	SAMN14596888	PRJNA614504	2020-3-30	North America	USA	Wisconsin	Dane county	North America	USA	Wisconsin	genome	29782	?	?	2020-4-12	18.74	19.89
USA/WI-UW-114/2020	EPI_ISL_425171	-	SRX8114924	SRR11544828	SAMN14596890	PRJNA614504	2020-3-30	North America	USA	Wisconsin	Dane county	North America	USA	Wisconsin	genome	29782	?	?	2020-4-12	22.62	23.5
USA/WI-UW-115/2020	EPI_ISL_425172	-	SRX8114926	SRR11544826	SAMN14596891	PRJNA614504	2020-3-31	North America	USA	Wisconsin	Dane county	North America	USA	Wisconsin	genome	29782	?	?	2020-4-12	26.2	-
USA/WI-UW-116/2020	EPI_ISL_425173	-	SRX8114927	SRR11544825	SAMN14596892	PRJNA614504	2020-3-30	North America	USA	Wisconsin	Dane county	North America	USA	Wisconsin	genome	29782	?	?	2020-4-12	31.81	33.31
USA/WI-UW-117/2020	EPI_ISL_425174	-	SRX8114928	SRR11544824	SAMN14596893	PRJNA614504	2020-3-30	North America	USA	Wisconsin	Dane county	North America	USA	Wisconsin	genome	29782	?	?	2020-4-12	28.2	-

USA/WI-UW-119/2020	EPI_ISL_425176	-	SRX8114930	SRR11544822	SAMN14596895	PRJNA614504	2020-4-10	North America	USA	Wisconsin	Dane county	North America	USA	Wisconsin	genome	29782	?	?	2020-4-12	31.01	32.71
USA/WI-UW-120/2020	EPI_ISL_427427	-	SRX8149929	SRR11582218	SAMN14654585	PRJNA614504	2020-4-13	North America	USA	Wisconsin	Dane county	North America	USA	Wisconsin	genome	29782	?	?	2020-4-18	14.76	14.82
USA/WI-UW-122/2020	EPI_ISL_427429	-	SRX8149905	SRR11582242	SAMN14654587	PRJNA614504	2020-4-7	North America	USA	Wisconsin	Dane county	North America	USA	Wisconsin	genome	29782	?	?	2020-4-18	28.1	-
USA/WI-UW-124/2020	EPI_ISL_427431	-	SRX8149923	SRR11582224	SAMN14654589	PRJNA614504	2020-4-7	North America	USA	Wisconsin	Dane county	North America	USA	Wisconsin	genome	29782	?	?	2020-4-18	17.5	-
USA/WI-UW-127/2020	EPI_ISL_427434	-	SRX8149926	SRR11582221	SAMN14654592	PRJNA614504	2020-4-7	North America	USA	Wisconsin	Dane county	North America	USA	Wisconsin	genome	29782	?	?	2020-4-18	19.1	-
USA/WI-UW-128/2020	EPI_ISL_427435	-	SRX8149927	SRR11582220	SAMN14654593	PRJNA614504	2020-4-7	North America	USA	Wisconsin	Dane county	North America	USA	Wisconsin	genome	29782	?	?	2020-4-18	21	-
USA/WI-UW-129/2020	EPI_ISL_427436	-	SRX8149928	SRR11582219	SAMN14654594	PRJNA614504	2020-4-6	North America	USA	Wisconsin	Dane county	North America	USA	Wisconsin	genome	29782	?	?	2020-4-18	30.7	-
USA/WI-UW-135/2020	EPI_ISL_427442	-	SRX8149900	SRR11582247	SAMN14654600	PRJNA614504	2020-4-9	North America	USA	Wisconsin	Dane county	North America	USA	Wisconsin	genome	29782	?	?	2020-4-18	22.5	-
USA/WI-UW-140/2020	EPI_ISL_427447	-	SRX8149906	SRR11582241	SAMN14654605	PRJNA614504	2020-4-9	North America	USA	Wisconsin	Dane county	North America	USA	Wisconsin	genome	29782	?	?	2020-4-18	21.43	-
USA/WI-UW-144/2020	EPI_ISL_427451	-	SRX8149910	SRR11582237	SAMN14654609	PRJNA614504	2020-4-6	North America	USA	Wisconsin	Dane county	North America	USA	Wisconsin	genome	29782	?	?	2020-4-18	23.92	24.31
USA/WI-UW-145/2020	EPI_ISL_427452	-	SRX8149911	SRR11582236	SAMN14654610	PRJNA614504	2020-4-10	North America	USA	Wisconsin	Dane county	North America	USA	Wisconsin	genome	29782	?	?	2020-4-18	27.68	28.81
USA/WI-UW-146/2020	EPI_ISL_427453	-	SRX8149912	SRR11582235	SAMN14654611	PRJNA614504	2020-4-6	North America	USA	Wisconsin	Dane county	North America	USA	Wisconsin	genome	29782	?	?	2020-4-18	26.1	-
USA/WI-UW-147/2020	EPI_ISL_427454	-	SRX8149913	SRR11582234	SAMN14654612	PRJNA614504	2020-4-10	North America	USA	Wisconsin	Dane county	North America	USA	Wisconsin	genome	29782	?	?	2020-4-18	34.6	-
USA/WI-UW-148/2020	EPI_ISL_427455	-	SRX8149914	SRR11582233	SAMN14654613	PRJNA614504	2020-4-7	North America	USA	Wisconsin	Dane county	North America	USA	Wisconsin	genome	29782	?	?	2020-4-18	20.7	-
USA/WI-UW-150/2020	EPI_ISL_427457	-	SRX8149917	SRR11582230	SAMN14654615	PRJNA614504	2020-4-13	North America	USA	Wisconsin	Dane county	North America	USA	Wisconsin	genome	29782	?	?	2020-4-18	29.7	-
USA/WI-UW-151/2020	EPI_ISL_427458	-	SRX8149918	SRR11582229	SAMN14654616	PRJNA614504	2020-4-8	North America	USA	Wisconsin	Dane county	North America	USA	Wisconsin	genome	29782	?	?	2020-4-18	23.56	24.33
USA/WI-UW-152/2020	EPI_ISL_427459	-	SRX8149919	SRR11582228	SAMN14654617	PRJNA614504	2020-4-6	North America	USA	Wisconsin	Dane county	North America	USA	Wisconsin	genome	29782	?	?	2020-4-18	35.7	-
USA/WI-UW-153/2020	EPI_ISL_427460	-	SRX8149920	SRR11582227	SAMN14654618	PRJNA614504	2020-4-9	North America	USA	Wisconsin	Dane county	North America	USA	Wisconsin	genome	29782	?	?	2020-4-18	15.1	-
USA/WI-UW-155/2020	EPI_ISL_427462	-	SRX8149922	SRR11582225	SAMN14654620	PRJNA614504	2020-4-10	North America	USA	Wisconsin	Dane county	North America	USA	Wisconsin	genome	29782	?	?	2020-4-18	33.3	-
USA/WI-UW-156/2020	EPI_ISL_428252	-	SRX8151738	SRR11584248	SAMN14656612	PRJNA614504	2020-3-25	North America	USA	Wisconsin	Milwaukee county	North America	USA	Wisconsin	genome	29782	?	?	2020-4-20	19.15	17.42
USA/WI-UW-157/2020	EPI_ISL_428253	-	SRX8151739	SRR11584247	SAMN14656613	PRJNA614504	2020-3-15	North America	USA	Wisconsin	Milwaukee county	North America	USA	Wisconsin	genome	29782	?	?	2020-4-20	20.2	20.46
USA/WI-UW-158/2020	EPI_ISL_428254	-	SRX8151750	SRR11584236	SAMN14656614	PRJNA614504	2020-3-15	North America	USA	Wisconsin	Milwaukee county	North America	USA	Wisconsin	genome	29782	?	?	2020-4-20	23.93	24.33
USA/WI-UW-159/2020	EPI_ISL_428255	-	SRX8151761	SRR11584225	SAMN14656615	PRJNA614504	2020-3-15	North America	USA	Wisconsin	Milwaukee county	North America	USA	Wisconsin	genome	29782	?	?	2020-4-20	20.57	21.06
USA/WI-UW-160/2020	EPI_ISL_428256	-	SRX8151772	SRR11584214	SAMN14656616	PRJNA614504	2020-3-15	North America	USA	Wisconsin	Milwaukee county	North America	USA	Wisconsin	genome	29782	?	?	2020-4-20	18.6	19.08
USA/WI-UW-161/2020	EPI_ISL_428257	-	SRX8151783	SRR11584203	SAMN14656617	PRJNA614504	2020-3-29	North America	USA	Wisconsin	Milwaukee county	North America	USA	Wisconsin	genome	29782	?	?	2020-4-20	23.88	24.27

USA/WI-UW-162/2020	EPI_ISL_428258	-	SRX8151794	SRR11584192	SAMN14656618	PRJNA614504	2020-3-29	North America	USA	Wisconsin	Milwaukee county	North America	USA	Wisconsin	genome	29782	?	?	2020-4-20	22.07	23.25
USA/WI-UW-165/2020	EPI_ISL_428261	-	SRX8151827	SRR11584159	SAMN14656621	PRJNA614504	2020-3-17	North America	USA	Wisconsin	Milwaukee county	North America	USA	Wisconsin	genome	29782	?	?	2020-4-20	23.24	22.81
USA/WI-UW-166/2020	EPI_ISL_428262	-	SRX8151740	SRR11584246	SAMN14656622	PRJNA614504	2020-3-17	North America	USA	Wisconsin	Milwaukee county	North America	USA	Wisconsin	genome	29782	?	?	2020-4-20	26.01	25.41
USA/WI-UW-168/2020	EPI_ISL_428264	-	SRX8151742	SRR11584244	SAMN14656624	PRJNA614504	2020-3-19	North America	USA	Wisconsin	Milwaukee county	North America	USA	Wisconsin	genome	29782	?	?	2020-4-20	26.49	26.74
USA/WI-UW-170/2020	EPI_ISL_428266	-	SRX8151744	SRR11584242	SAMN14656626	PRJNA614504	2020-3-19	North America	USA	Wisconsin	Milwaukee county	North America	USA	Wisconsin	genome	29782	?	?	2020-4-20	21.13	21.33
USA/WI-UW-171/2020	EPI_ISL_428267	-	SRX8151745	SRR11584241	SAMN14656627	PRJNA614504	2020-3-19	North America	USA	Wisconsin	Milwaukee county	North America	USA	Wisconsin	genome	29782	?	?	2020-4-20	22.38	22.8
USA/WI-UW-172/2020	EPI_ISL_428268	-	SRX8151746	SRR11584240	SAMN14656628	PRJNA614504	2020-3-19	North America	USA	Wisconsin	Milwaukee county	North America	USA	Wisconsin	genome	29782	?	?	2020-4-20	21.67	21.8
USA/WI-UW-173/2020	EPI_ISL_428269	-	SRX8151747	SRR11584239	SAMN14656629	PRJNA614504	2020-3-20	North America	USA	Wisconsin	Milwaukee county	North America	USA	Wisconsin	genome	29782	?	?	2020-4-20	23.85	23.92
USA/WI-UW-176/2020	EPI_ISL_428272	-	SRX8151751	SRR11584235	SAMN14656632	PRJNA614504	2020-3-20	North America	USA	Wisconsin	Milwaukee county	North America	USA	Wisconsin	genome	29782	?	?	2020-4-20	21.51	21.64
USA/WI-UW-177/2020	EPI_ISL_428273	-	SRX8151752	SRR11584234	SAMN14656633	PRJNA614504	2020-3-20	North America	USA	Wisconsin	Milwaukee county	North America	USA	Wisconsin	genome	29782	?	?	2020-4-20	28.17	28.5
USA/WI-UW-178/2020	EPI_ISL_428274	-	SRX8151753	SRR11584233	SAMN14656634	PRJNA614504	2020-3-20	North America	USA	Wisconsin	Milwaukee county	North America	USA	Wisconsin	genome	29782	?	?	2020-4-20	24.44	26.65
USA/WI-UW-179/2020	EPI_ISL_428275	-	SRX8151754	SRR11584232	SAMN14656635	PRJNA614504	2020-3-21	North America	USA	Wisconsin	Milwaukee county	North America	USA	Wisconsin	genome	29782	?	?	2020-4-20	19.89	18.58
USA/WI-UW-181/2020	EPI_ISL_428277	-	SRX8151756	SRR11584230	SAMN14656637	PRJNA614504	2020-3-22	North America	USA	Wisconsin	Milwaukee county	North America	USA	Wisconsin	genome	29782	?	?	2020-4-20	28.41	29.61
USA/WI-UW-182/2020	EPI_ISL_428278	-	SRX8151757	SRR11584229	SAMN14656638	PRJNA614504	2020-3-22	North America	USA	Wisconsin	Milwaukee county	North America	USA	Wisconsin	genome	29782	?	?	2020-4-20	24.75	25.09
USA/WI-UW-183/2020	EPI_ISL_428279	-	SRX8151758	SRR11584228	SAMN14656639	PRJNA614504	2020-3-22	North America	USA	Wisconsin	Milwaukee county	North America	USA	Wisconsin	genome	29782	?	?	2020-4-20	25.76	25.88
USA/WI-UW-184/2020	EPI_ISL_428280	-	SRX8151759	SRR11584227	SAMN14656640	PRJNA614504	2020-3-22	North America	USA	Wisconsin	Milwaukee county	North America	USA	Wisconsin	genome	29782	?	?	2020-4-20	21.9	22.26
USA/WI-UW-185/2020	EPI_ISL_428281	-	SRX8151760	SRR11584226	SAMN14656641	PRJNA614504	2020-3-22	North America	USA	Wisconsin	Milwaukee county	North America	USA	Wisconsin	genome	29782	?	?	2020-4-20	19.97	20.7
USA/WI-UW-186/2020	EPI_ISL_428282	-	SRX8151762	SRR11584224	SAMN14656642	PRJNA614504	2020-3-23	North America	USA	Wisconsin	Milwaukee county	North America	USA	Wisconsin	genome	29782	?	?	2020-4-20	16.31	15.63
USA/WI-UW-187/2020	EPI_ISL_428283	-	SRX8151763	SRR11584223	SAMN14656643	PRJNA614504	2020-3-23	North America	USA	Wisconsin	Milwaukee county	North America	USA	Wisconsin	genome	29782	?	?	2020-4-20	22.5	21.62
USA/WI-UW-188/2020	EPI_ISL_428284	-	SRX8151764	SRR11584222	SAMN14656644	PRJNA614504	2020-3-23	North America	USA	Wisconsin	Milwaukee county	North America	USA	Wisconsin	genome	29782	?	?	2020-4-20	30.05	29.06
USA/WI-UW-189/2020	EPI_ISL_428285	-	SRX8151765	SRR11584221	SAMN14656645	PRJNA614504	2020-3-23	North America	USA	Wisconsin	Milwaukee county	North America	USA	Wisconsin	genome	29782	?	?	2020-4-20	25.05	24.1
USA/WI-UW-190/2020	EPI_ISL_428286	-	SRX8151766	SRR11584220	SAMN14656646	PRJNA614504	2020-3-23	North America	USA	Wisconsin	Milwaukee county	North America	USA	Wisconsin	genome	29782	?	?	2020-4-20	15.76	15.34
USA/WI-UW-191/2020	EPI_ISL_428287	-	SRX8151767	SRR11584219	SAMN14656647	PRJNA614504	2020-3-23	North America	USA	Wisconsin	Milwaukee county	North America	USA	Wisconsin	genome	29782	?	?	2020-4-20	15.52	15.23
USA/WI-UW-192/2020	EPI_ISL_428288	-	SRX8151768	SRR11584218	SAMN14656648	PRJNA614504	2020-3-23	North America	USA	Wisconsin	Milwaukee county	North America	USA	Wisconsin	genome	29782	?	?	2020-4-20	28.48	28.3
USA/WI-UW-195/2020	EPI_ISL_428291	-	SRX8151771	SRR11584215	SAMN14656651	PRJNA614504	2020-3-24	North America	USA	Wisconsin	Milwaukee county	North America	USA	Wisconsin	genome	29782	?	?	2020-4-20	19.21	18.41

USA/WI-UW-196/2020	EPI_ISL_428292	-	SRX8151773	SRR11584213	SAMN14656652	PRJNA614504	2020-3-21	North America	USA	Wisconsin	Milwaukee county	North America	USA	Wisconsin	genome	29782	?	?	2020-4-20	28.66	29.46
USA/WI-UW-197/2020	EPI_ISL_428293	-	SRX8151774	SRR11584212	SAMN14656653	PRJNA614504	2020-3-24	North America	USA	Wisconsin	Milwaukee county	North America	USA	Wisconsin	genome	29782	?	?	2020-4-20	27.82	27.23
USA/WI-UW-200/2020	EPI_ISL_428296	-	SRX8151777	SRR11584209	SAMN14656656	PRJNA614504	2020-3-24	North America	USA	Wisconsin	Milwaukee county	North America	USA	Wisconsin	genome	29782	?	?	2020-4-20	17.64	17.78
USA/WI-UW-205/2020	EPI_ISL_428301	-	SRX8151782	SRR11584204	SAMN14656661	PRJNA614504	2020-3-25	North America	USA	Wisconsin	Milwaukee county	North America	USA	Wisconsin	genome	29782	?	?	2020-4-20	27.03	27.23
USA/WI-UW-207/2020	EPI_ISL_428303	-	SRX8151785	SRR11584201	SAMN14656663	PRJNA614504	2020-3-23	North America	USA	Wisconsin	Milwaukee county	North America	USA	Wisconsin	genome	29782	?	?	2020-4-20	24.78	25.02
USA/WI-UW-208/2020	EPI_ISL_428304	-	SRX8151786	SRR11584200	SAMN14656664	PRJNA614504	2020-3-25	North America	USA	Wisconsin	Milwaukee county	North America	USA	Wisconsin	genome	29782	?	?	2020-4-20	25.72	24.87
USA/WI-UW-209/2020	EPI_ISL_428305	-	SRX8151787	SRR11584199	SAMN14656665	PRJNA614504	2020-3-25	North America	USA	Wisconsin	Milwaukee county	North America	USA	Wisconsin	genome	29782	?	?	2020-4-20	17.48	16.62
USA/WI-UW-210/2020	EPI_ISL_428306	-	SRX8151788	SRR11584198	SAMN14656666	PRJNA614504	2020-3-24	North America	USA	Wisconsin	Milwaukee county	North America	USA	Wisconsin	genome	29782	?	?	2020-4-20	24.67	24.17
USA/WI-UW-211/2020	EPI_ISL_428307	-	SRX8151789	SRR11584197	SAMN14656667	PRJNA614504	2020-3-25	North America	USA	Wisconsin	Milwaukee county	North America	USA	Wisconsin	genome	29782	?	?	2020-4-20	26.72	27.21
USA/WI-UW-212/2020	EPI_ISL_428308	-	SRX8151790	SRR11584196	SAMN14656668	PRJNA614504	2020-3-25	North America	USA	Wisconsin	Milwaukee county	North America	USA	Wisconsin	genome	29782	?	?	2020-4-20	26.05	26.57
USA/WI-UW-213/2020	EPI_ISL_428309	-	SRX8151791	SRR11584195	SAMN14656669	PRJNA614504	2020-3-25	North America	USA	Wisconsin	Milwaukee county	North America	USA	Wisconsin	genome	29782	?	?	2020-4-20	25.77	26.74
USA/WI-UW-215/2020	EPI_ISL_428311	-	SRX8151793	SRR11584193	SAMN14656671	PRJNA614504	2020-3-25	North America	USA	Wisconsin	Milwaukee county	North America	USA	Wisconsin	genome	29782	?	?	2020-4-20	29.01	29.94
USA/WI-UW-216/2020	EPI_ISL_428312	-	SRX8151795	SRR11584191	SAMN14656672	PRJNA614504	2020-3-25	North America	USA	Wisconsin	Milwaukee county	North America	USA	Wisconsin	genome	29782	?	?	2020-4-20	28.39	29.36
USA/WI-UW-217/2020	EPI_ISL_428313	-	SRX8151796	SRR11584190	SAMN14656673	PRJNA614504	2020-3-25	North America	USA	Wisconsin	Milwaukee county	North America	USA	Wisconsin	genome	29782	?	?	2020-4-20	29.14	29.6
USA/WI-UW-218/2020	EPI_ISL_428314	-	SRX8151797	SRR11584189	SAMN14656674	PRJNA614504	2020-3-25	North America	USA	Wisconsin	Milwaukee county	North America	USA	Wisconsin	genome	29782	?	?	2020-4-20	26.03	27.14
USA/WI-UW-219/2020	EPI_ISL_428315	-	SRX8151798	SRR11584188	SAMN14656675	PRJNA614504	2020-3-25	North America	USA	Wisconsin	Milwaukee county	North America	USA	Wisconsin	genome	29782	?	?	2020-4-20	21.97	22.39
USA/WI-UW-221/2020	EPI_ISL_428317	-	SRX8151800	SRR11584186	SAMN14656677	PRJNA614504	2020-3-25	North America	USA	Wisconsin	Milwaukee county	North America	USA	Wisconsin	genome	29782	?	?	2020-4-20	16.03	15.91
USA/WI-UW-222/2020	EPI_ISL_428318	-	SRX8151801	SRR11584185	SAMN14656678	PRJNA614504	2020-3-25	North America	USA	Wisconsin	Milwaukee county	North America	USA	Wisconsin	genome	29782	?	?	2020-4-20	24.65	25.06
USA/WI-UW-223/2020	EPI_ISL_428319	-	SRX8151802	SRR11584184	SAMN14656679	PRJNA614504	2020-3-26	North America	USA	Wisconsin	Milwaukee county	North America	USA	Wisconsin	genome	29782	?	?	2020-4-20	26.67	28.25
USA/WI-UW-225/2020	EPI_ISL_428321	-	SRX8151804	SRR11584182	SAMN14656681	PRJNA614504	2020-3-26	North America	USA	Wisconsin	Milwaukee county	North America	USA	Wisconsin	genome	29782	?	?	2020-4-20	18.55	18.91
USA/WI-UW-226/2020	EPI_ISL_428322	-	SRX8151806	SRR11584180	SAMN14656682	PRJNA614504	2020-3-26	North America	USA	Wisconsin	Milwaukee county	North America	USA	Wisconsin	genome	29782	?	?	2020-4-20	18.81	19.22
USA/WI-UW-227/2020	EPI_ISL_428323	-	SRX8151807	SRR11584179	SAMN14656683	PRJNA614504	2020-3-26	North America	USA	Wisconsin	Milwaukee county	North America	USA	Wisconsin	genome	29782	?	?	2020-4-20	22.54	23.52
USA/WI-UW-228/2020	EPI_ISL_428324	-	SRX8151808	SRR11584178	SAMN14656684	PRJNA614504	2020-3-26	North America	USA	Wisconsin	Milwaukee county	North America	USA	Wisconsin	genome	29782	?	?	2020-4-20	17.61	19.04
USA/WI-UW-229/2020	EPI_ISL_428325	-	SRX8151809	SRR11584177	SAMN14656685	PRJNA614504	2020-3-26	North America	USA	Wisconsin	Milwaukee county	North America	USA	Wisconsin	genome	29782	?	?	2020-4-20	17.43	18.08
USA/WI-UW-230/2020	EPI_ISL_428326	-	SRX8151810	SRR11584176	SAMN14656686	PRJNA614504	2020-3-26	North America	USA	Wisconsin	Milwaukee county	North America	USA	Wisconsin	genome	29782	?	?	2020-4-20	24.4	25.99

USA/WI-UW-231/2020	EPI_ISL_428327	-	SRX8151811	SRR11584175	SAMN14656687	PRJNA614504	2020-3-26	North America	USA	Wisconsin	Milwaukee county	North America	USA	Wisconsin	genome	29782	?	?	2020-4-20	19.42	19.94
USA/WI-UW-232/2020	EPI_ISL_428328	-	SRX8151812	SRR11584174	SAMN14656688	PRJNA614504	2020-3-27	North America	USA	Wisconsin	Milwaukee county	North America	USA	Wisconsin	genome	29782	?	?	2020-4-20	19.54	18.33
USA/WI-UW-233/2020	EPI_ISL_428329	-	SRX8151813	SRR11584173	SAMN14656689	PRJNA614504	2020-3-27	North America	USA	Wisconsin	Milwaukee county	North America	USA	Wisconsin	genome	29782	?	?	2020-4-20	26.33	26.12
USA/WI-UW-234/2020	EPI_ISL_428330	-	SRX8151814	SRR11584172	SAMN14656690	PRJNA614504	2020-3-26	North America	USA	Wisconsin	Milwaukee county	North America	USA	Wisconsin	genome	29782	?	?	2020-4-20	19.88	18.62
USA/WI-UW-235/2020	EPI_ISL_428331	-	SRX8151815	SRR11584171	SAMN14656691	PRJNA614504	2020-3-28	North America	USA	Wisconsin	Milwaukee county	North America	USA	Wisconsin	genome	29782	?	?	2020-4-20	26.87	17.15
USA/WI-UW-236/2020	EPI_ISL_428332	-	SRX8151817	SRR11584169	SAMN14656692	PRJNA614504	2020-3-27	North America	USA	Wisconsin	Milwaukee county	North America	USA	Wisconsin	genome	29782	?	?	2020-4-20	23.37	23.27
USA/WI-UW-237/2020	EPI_ISL_428333	-	SRX8151818	SRR11584168	SAMN14656693	PRJNA614504	2020-3-27	North America	USA	Wisconsin	Milwaukee county	North America	USA	Wisconsin	genome	29782	?	?	2020-4-20	18.05	17.98
USA/WI-UW-238/2020	EPI_ISL_428334	-	SRX8151819	SRR11584167	SAMN14656694	PRJNA614504	2020-3-27	North America	USA	Wisconsin	Milwaukee county	North America	USA	Wisconsin	genome	29782	?	?	2020-4-20	26.38	25.52
USA/WI-UW-240/2020	EPI_ISL_428336	-	SRX8151821	SRR11584165	SAMN14656696	PRJNA614504	2020-3-27	North America	USA	Wisconsin	Milwaukee county	North America	USA	Wisconsin	genome	29782	?	?	2020-4-20	24.49	24.26
USA/WI-UW-241/2020	EPI_ISL_428337	-	SRX8151822	SRR11584164	SAMN14656697	PRJNA614504	2020-3-27	North America	USA	Wisconsin	Milwaukee county	North America	USA	Wisconsin	genome	29782	?	?	2020-4-20	17.33	16.59
USA/WI-UW-242/2020	EPI_ISL_428338	-	SRX8151823	SRR11584163	SAMN14656698	PRJNA614504	2020-3-28	North America	USA	Wisconsin	Milwaukee county	North America	USA	Wisconsin	genome	29782	?	?	2020-4-20	17.93	17.52
USA/WI-UW-245/2020	EPI_ISL_428341	-	SRX8151826	SRR11584160	SAMN14656701	PRJNA614504	2020-3-28	North America	USA	Wisconsin	Milwaukee county	North America	USA	Wisconsin	genome	29782	?	?	2020-4-20	28.3	28.08
USA/WI-UW-246/2020	EPI_ISL_428342	-	SRX8151828	SRR11584158	SAMN14656702	PRJNA614504	2020-3-28	North America	USA	Wisconsin	Milwaukee county	North America	USA	Wisconsin	genome	29782	?	?	2020-4-20	22.16	21.97
USA/WI-UW-248/2020	EPI_ISL_428344	-	SRX8151830	SRR11584156	SAMN14656704	PRJNA614504	2020-3-28	North America	USA	Wisconsin	Milwaukee county	North America	USA	Wisconsin	genome	29782	?	?	2020-4-20	25.24	24.96
USA/WI-UW-259/2020	EPI_ISL_428935	-	SRX8155700	SRR11588239	SAMN14669377	PRJNA614504	2020-4-17	North America	USA	Wisconsin	Dane county	North America	USA	Wisconsin	genome	29611	?	?	2020-4-22	23.8	-
USA/WI-UW-260/2020	EPI_ISL_428936	-	SRX8155701	SRR11588238	SAMN14669378	PRJNA614504	2020-4-18	North America	USA	Wisconsin	Dane county	North America	USA	Wisconsin	genome	29782	?	?	2020-4-22	32.8	-
USA/WI-UW-270/2020	EPI_ISL_436564	-	SRX8281160	SRR11721857	SAMN14844834	PRJNA614504	2020-3-12	North America	USA	Wisconsin	Milwaukee county	North America	USA	Wisconsin	genome	29611	?	?	2020-5-6	25.42	26.85
USA/WI-UW-272/2020	EPI_ISL_436566	-	SRX8281172	SRR11721845	SAMN14844836	PRJNA614504	2020-3-27	North America	USA	Wisconsin	Milwaukee county	North America	USA	Wisconsin	genome	29782	?	?	2020-5-6	19.54	18.33
USA/WI-UW-273/2020	EPI_ISL_436567	-	SRX8281183	SRR11721834	SAMN14844837	PRJNA614504	2020-3-31	North America	USA	Wisconsin	Milwaukee county	North America	USA	Wisconsin	genome	29782	?	?	2020-5-6	20.29	20.47
USA/WI-UW-274/2020	EPI_ISL_436568	-	SRX8281194	SRR11721823	SAMN14844838	PRJNA614504	2020-3-31	North America	USA	Wisconsin	Milwaukee county	North America	USA	Wisconsin	genome	29782	?	?	2020-5-6	25.25	25.5
USA/WI-UW-275/2020	EPI_ISL_436569	-	SRX8281128	SRR11721889	SAMN14844839	PRJNA614504	2020-4-1	North America	USA	Wisconsin	Milwaukee county	North America	USA	Wisconsin	genome	29782	?	?	2020-5-6	18.54	18.14
USA/WI-UW-276/2020	EPI_ISL_436570	-	SRX8281139	SRR11721878	SAMN14844840	PRJNA614504	2020-4-1	North America	USA	Wisconsin	Milwaukee county	North America	USA	Wisconsin	genome	29782	?	?	2020-5-6	19.27	19.02
USA/WI-UW-277/2020	EPI_ISL_436571	-	SRX8281150	SRR11721867	SAMN14844841	PRJNA614504	2020-4-2	North America	USA	Wisconsin	Milwaukee county	North America	USA	Wisconsin	genome	29782	?	?	2020-5-6	20.28	20.06
USA/WI-UW-278/2020	EPI_ISL_436572	-	SRX8281158	SRR11721859	SAMN14844842	PRJNA614504	2020-4-3	North America	USA	Wisconsin	Milwaukee county	North America	USA	Wisconsin	genome	29782	?	?	2020-5-6	16.08	16.12
USA/WI-UW-279/2020	EPI_ISL_436573	-	SRX8281159	SRR11721858	SAMN14844843	PRJNA614504	2020-4-3	North America	USA	Wisconsin	Milwaukee county	North America	USA	Wisconsin	genome	29782	?	?	2020-5-6	15.6	15.35

USA/WI-UW-280/2020	EPI_ISL_436574	-	SRX8281162	SRR11721855	SAMN14844844	PRJNA614504	2020-4-3	North America	USA	Wisconsin	Milwaukee county	North America	USA	Wisconsin	genome	29782	?	?	2020-5-6	17.49	16.11
USA/WI-UW-285/2020	EPI_ISL_436579	-	SRX8281167	SRR11721850	SAMN14844849	PRJNA614504	2020-4-6	North America	USA	Wisconsin	Milwaukee county	North America	USA	Wisconsin	genome	29782	?	?	2020-5-6	27.55	27.17
USA/WI-UW-288/2020	EPI_ISL_436582	-	SRX8281170	SRR11721847	SAMN14844852	PRJNA614504	2020-4-8	North America	USA	Wisconsin	Milwaukee county	North America	USA	Wisconsin	genome	29782	?	?	2020-5-6	20.86	20
USA/WI-UW-289/2020	EPI_ISL_436583	-	SRX8281171	SRR11721846	SAMN14844853	PRJNA614504	2020-4-8	North America	USA	Wisconsin	Milwaukee county	North America	USA	Wisconsin	genome	29782	?	?	2020-5-6	19.69	19.05
USA/WI-UW-290/2020	EPI_ISL_436584	-	SRX8281173	SRR11721844	SAMN14844854	PRJNA614504	2020-4-8	North America	USA	Wisconsin	Milwaukee county	North America	USA	Wisconsin	genome	29782	?	?	2020-5-6	16.04	15.4
USA/WI-UW-291/2020	EPI_ISL_436585	-	SRX8281174	SRR11721843	SAMN14844855	PRJNA614504	2020-4-8	North America	USA	Wisconsin	Milwaukee county	North America	USA	Wisconsin	genome	29782	?	?	2020-5-6	21.7	21.27
USA/WI-UW-293/2020	EPI_ISL_436587	-	SRX8281176	SRR11721841	SAMN14844857	PRJNA614504	2020-4-9	North America	USA	Wisconsin	Milwaukee county	North America	USA	Wisconsin	genome	29782	?	?	2020-5-6	21.29	20.93
USA/WI-UW-296/2020	EPI_ISL_436590	-	SRX8281179	SRR11721838	SAMN14844860	PRJNA614504	2020-4-9	North America	USA	Wisconsin	Milwaukee county	North America	USA	Wisconsin	genome	29782	?	?	2020-5-6	16.13	15.52
USA/WI-UW-299/2020	EPI_ISL_436593	-	SRX8281182	SRR11721835	SAMN14844863	PRJNA614504	2020-4-13	North America	USA	Wisconsin	Milwaukee county	North America	USA	Wisconsin	genome	29782	?	?	2020-5-6	18.84	18.37
USA/WI-UW-301/2020	EPI_ISL_436595	-	SRX8281185	SRR11721832	SAMN14844865	PRJNA614504	2020-4-13	North America	USA	Wisconsin	Milwaukee county	North America	USA	Wisconsin	genome	29782	?	?	2020-5-6	25.41	25.87
USA/WI-UW-302/2020	EPI_ISL_436596	-	SRX8281186	SRR11721831	SAMN14844866	PRJNA614504	2020-4-13	North America	USA	Wisconsin	Milwaukee county	North America	USA	Wisconsin	genome	29782	?	?	2020-5-6	17.6	17.08
USA/WI-UW-303/2020	EPI_ISL_436597	-	SRX8281187	SRR11721830	SAMN14844867	PRJNA614504	2020-4-13	North America	USA	Wisconsin	Milwaukee county	North America	USA	Wisconsin	genome	29782	?	?	2020-5-6	19.48	18.83
USA/WI-UW-304/2020	EPI_ISL_436598	-	SRX8281188	SRR11721829	SAMN14844868	PRJNA614504	2020-4-13	North America	USA	Wisconsin	Milwaukee county	North America	USA	Wisconsin	genome	29782	?	?	2020-5-6	18.58	18.69
USA/WI-UW-305/2020	EPI_ISL_436599	-	SRX8281189	SRR11721828	SAMN14844869	PRJNA614504	2020-4-14	North America	USA	Wisconsin	Milwaukee county	North America	USA	Wisconsin	genome	29782	?	?	2020-5-6	22.12	21.58
USA/WI-UW-306/2020	EPI_ISL_436600	-	SRX8281190	SRR11721827	SAMN14844870	PRJNA614504	2020-4-14	North America	USA	Wisconsin	Milwaukee county	North America	USA	Wisconsin	genome	29782	?	?	2020-5-6	30.42	31.22
USA/WI-UW-308/2020	EPI_ISL_436602	-	SRX8281192	SRR11721825	SAMN14844872	PRJNA614504	2020-4-16	North America	USA	Wisconsin	Milwaukee county	North America	USA	Wisconsin	genome	29782	?	?	2020-5-6	24.51	25.54
USA/WI-UW-309/2020	EPI_ISL_436603	-	SRX8281193	SRR11721824	SAMN14844873	PRJNA614504	2020-4-16	North America	USA	Wisconsin	Milwaukee county	North America	USA	Wisconsin	genome	29782	?	?	2020-5-6	19.7	22.2
USA/WI-UW-310/2020	EPI_ISL_436604	-	SRX8281195	SRR11721822	SAMN14844874	PRJNA614504	2020-4-15	North America	USA	Wisconsin	Milwaukee county	North America	USA	Wisconsin	genome	29782	?	?	2020-5-6	20.64	19.45
USA/WI-UW-313/2020	EPI_ISL_436607	-	SRX8281121	SRR11721896	SAMN14844877	PRJNA614504	2020-4-16	North America	USA	Wisconsin	Milwaukee county	North America	USA	Wisconsin	genome	29782	?	?	2020-5-6	21.34	21.33
USA/WI-UW-316/2020	EPI_ISL_436610	-	SRX8281124	SRR11721893	SAMN14844880	PRJNA614504	2020-4-18	North America	USA	Wisconsin	Milwaukee county	North America	USA	Wisconsin	genome	29782	?	?	2020-5-6	24.17	23.31
USA/WI-UW-319/2020	EPI_ISL_436613	-	SRX8281127	SRR11721890	SAMN14844883	PRJNA614504	2020-4-21	North America	USA	Wisconsin	Milwaukee county	North America	USA	Wisconsin	genome	29782	?	?	2020-5-6	25.41	27
USA/WI-UW-320/2020	EPI_ISL_436614	-	SRX8281129	SRR11721888	SAMN14844884	PRJNA614504	2020-4-21	North America	USA	Wisconsin	Milwaukee county	North America	USA	Wisconsin	genome	29782	?	?	2020-5-6	33.72	34.07
USA/WI-UW-321/2020	EPI_ISL_436615	-	SRX8281130	SRR11721887	SAMN14844885	PRJNA614504	2020-4-23	North America	USA	Wisconsin	Milwaukee county	North America	USA	Wisconsin	genome	29782	?	?	2020-5-6	24.17	23.75
USA/WI-UW-322/2020	EPI_ISL_436616	-	SRX8281131	SRR11721886	SAMN14844886	PRJNA614504	2020-4-23	North America	USA	Wisconsin	Milwaukee county	North America	USA	Wisconsin	genome	29782	?	?	2020-5-6	27.3	29.9
USA/WI-UW-323/2020	EPI_ISL_436617	-	SRX8281132	SRR11721885	SAMN14844887	PRJNA614504	2020-4-23	North America	USA	Wisconsin	Milwaukee county	North America	USA	Wisconsin	genome	29782	?	?	2020-5-6	26.2	28.6

USA/WI-UW-324/2020	EPI_ISL_436618	-	SRX8281133	SRR11721884	SAMN14844888	PRJNA614504	2020-4-24	North America	USA	Wisconsin	Milwaukee county	North America	USA	Wisconsin	genome	29782	?	?	2020-5-6	22.5	22.82
USA/WI-UW-325/2020	EPI_ISL_436619	-	SRX8281134	SRR11721883	SAMN14844889	PRJNA614504	2020-4-24	North America	USA	Wisconsin	Milwaukee county	North America	USA	Wisconsin	genome	29782	?	?	2020-5-6	25.06	25.75
USA/WI-UW-328/2020	EPI_ISL_436622	-	SRX8281137	SRR11721880	SAMN14844892	PRJNA614504	2020-4-26	North America	USA	Wisconsin	Milwaukee county	North America	USA	Wisconsin	genome	29782	?	?	2020-5-6	23.92	24.55
USA/WI-UW-329/2020	EPI_ISL_436623	-	SRX8281138	SRR11721879	SAMN14844893	PRJNA614504	2020-4-25	North America	USA	Wisconsin	Milwaukee county	North America	USA	Wisconsin	genome	29782	?	?	2020-5-6	19.32	18.26
USA/WI-UW-330/2020	EPI_ISL_436624	-	SRX8281140	SRR11721877	SAMN14844894	PRJNA614504	2020-4-26	North America	USA	Wisconsin	Milwaukee county	North America	USA	Wisconsin	genome	29611	?	?	2020-5-6	27.34	27.27
USA/WI-UW-331/2020	EPI_ISL_436625	-	SRX8281141	SRR11721876	SAMN14844895	PRJNA614504	2020-4-26	North America	USA	Wisconsin	Milwaukee county	North America	USA	Wisconsin	genome	29782	?	?	2020-5-6	29.14	29.12
USA/WI-UW-332/2020	EPI_ISL_436626	-	SRX8281142	SRR11721875	SAMN14844896	PRJNA614504	2020-4-26	North America	USA	Wisconsin	Milwaukee county	North America	USA	Wisconsin	genome	29782	?	?	2020-5-6	15.15	15.39
USA/WI-UW-333/2020	EPI_ISL_436627	-	SRX8281143	SRR11721874	SAMN14844897	PRJNA614504	2020-3-24	North America	USA	Wisconsin	Milwaukee county	North America	USA	Wisconsin	genome	29782	?	?	2020-5-6	27.45	26.54
USA/WI-UW-334/2020	EPI_ISL_436628	-	SRX8281144	SRR11721873	SAMN14844898	PRJNA614504	2020-3-24	North America	USA	Wisconsin	Milwaukee county	North America	USA	Wisconsin	genome	29782	?	?	2020-5-6	23.24	22.79
USA/WI-UW-335/2020	EPI_ISL_436629	-	SRX8281145	SRR11721872	SAMN14844899	PRJNA614504	2020-3-24	North America	USA	Wisconsin	Milwaukee county	North America	USA	Wisconsin	genome	29782	?	?	2020-5-6	30.14	30.69
USA/WI-UW-336/2020	EPI_ISL_436630	-	SRX8281146	SRR11721871	SAMN14844900	PRJNA614504	2020-3-25	North America	USA	Wisconsin	Milwaukee county	North America	USA	Wisconsin	genome	29782	?	?	2020-5-6	19.15	17.42
USA/WI-UW-337/2020	EPI_ISL_436631	-	SRX8281147	SRR11721870	SAMN14844901	PRJNA614504	2020-3-26	North America	USA	Wisconsin	Milwaukee county	North America	USA	Wisconsin	genome	29782	?	?	2020-5-6	26.46	26.5
USA/WI-UW-338/2020	EPI_ISL_436632	-	SRX8281148	SRR11721869	SAMN14844902	PRJNA614504	2020-3-26	North America	USA	Wisconsin	Milwaukee county	North America	USA	Wisconsin	genome	29782	?	?	2020-5-6	27.72	27.6
USA/WI-UW-339/2020	EPI_ISL_436633	-	SRX8281149	SRR11721868	SAMN14844903	PRJNA614504	2020-3-31	North America	USA	Wisconsin	Milwaukee county	North America	USA	Wisconsin	genome	29611	?	?	2020-5-6	33.1	32.57
USA/WI-UW-340/2020	EPI_ISL_436634	-	SRX8281151	SRR11721866	SAMN14844904	PRJNA614504	2020-4-1	North America	USA	Wisconsin	Milwaukee county	North America	USA	Wisconsin	genome	29782	?	?	2020-5-6	35.15	33.47
USA/WI-UW-341/2020	EPI_ISL_436635	-	SRX8281152	SRR11721865	SAMN14844905	PRJNA614504	2020-4-2	North America	USA	Wisconsin	Milwaukee county	North America	USA	Wisconsin	genome	29782	?	?	2020-5-6	23.18	22.53
USA/WI-UW-342/2020	EPI_ISL_436636	-	SRX8281153	SRR11721864	SAMN14844906	PRJNA614504	2020-4-4	North America	USA	Wisconsin	Milwaukee county	North America	USA	Wisconsin	genome	29782	?	?	2020-5-6	39.56	33.93
USA/WI-UW-343/2020	EPI_ISL_436637	-	SRX8281154	SRR11721863	SAMN14844907	PRJNA614504	2020-4-5	North America	USA	Wisconsin	Milwaukee county	North America	USA	Wisconsin	genome	29782	?	?	2020-5-6	27.1	27.73
USA/WI-UW-344/2020	EPI_ISL_436638	-	SRX8281155	SRR11721862	SAMN14844908	PRJNA614504	2020-4-6	North America	USA	Wisconsin	Milwaukee county	North America	USA	Wisconsin	genome	29782	?	?	2020-5-6	38.04	37.08
USA/WI-UW-345/2020	EPI_ISL_436639	-	SRX8281156	SRR11721861	SAMN14844909	PRJNA614504	2020-4-9	North America	USA	Wisconsin	Milwaukee county	North America	USA	Wisconsin	genome	29611	?	?	2020-5-6	32	31.81
USA/WI-UW-346/2020	EPI_ISL_436640	-	SRX8281157	SRR11721860	SAMN14844910	PRJNA614504	2020-4-21	North America	USA	Wisconsin	Milwaukee county	North America	USA	Wisconsin	genome	29782	?	?	2020-5-6	27.37	27.39
USA/WI-UW-347/2020	EPI_ISL_450701	MT506886	SRX8379446	SRR11828929	SAMN14995484	PRJNA614504	2020-4-18	North America	USA	Wisconsin	Dane county	North America	USA	Wisconsin	genome	29782	?	?	2020-5-8	14	-
USA/WI-UW-351/2020	EPI_ISL_450705	MT506890	SRX8379463	SRR11828912	SAMN14995488	PRJNA614504	2020-4-14	North America	USA	Wisconsin	Dane county	North America	USA	Wisconsin	genome	29782	?	?	2020-5-8	25.51	26.64
USA/WI-UW-356/2020	EPI_ISL_450710	MT506895	SRX8379447	SRR11828928	SAMN14995493	PRJNA614504	2020-4-14	North America	USA	Wisconsin	Dane county	North America	USA	Wisconsin	genome	29782	?	?	2020-5-8	25.55	26.57
USA/WI-UW-359/2020	EPI_ISL_450713	MT506898	SRX8379450	SRR11828925	SAMN14995496	PRJNA614504	2020-4-18	North America	USA	Wisconsin	Dane county	North America	USA	Wisconsin	genome	29782	?	?	2020-5-8	17.2	-

USA/WI-UW-362/2020	EPI_ISL_450716	MT506901	SRX8379453	SRR11828922	SAMN14995499	PRJNA614504	2020-4-17	North America	USA	Wisconsin	Dane county	North America	USA	Wisconsin	genome	29782	?	?	2020-5-8	35.7	-

Supplementary Table 3. GISAID accession numbers and associated information for all consensus sequences included in this manuscript.

name	pool	sequence	length	%gc	tm (use 65)
nCoV-2019_1_LEFT	nCoV-2019_1	ACCAACCAACTTTCG ATCTCTTGT	24	41.67	60.69
nCoV-2019_1_RIGHT	nCoV-2019_1	CATCTTTAAGATGTTG ACGTGCCTC	25	44	60.45
nCoV-2019_2_LEFT	nCoV-2019_2	CTGTTTTACAGGTTC GCGACGT	22	50	61.67
nCoV-2019_2_RIGHT	nCoV-2019_2	TAAGGATCAGTGCCA AGCTCGT	22	50	61.74
nCoV-2019_3_LEFT	nCoV-2019_1	CGGTAATAAAGGAGC TGGTGGC	22	54.55	61.32
nCoV-2019_3_RIGHT	nCoV-2019_1	AAGGTGTCTGCAATT CATAGCTCT	24	41.67	60.32
nCoV-2019_4_LEFT	nCoV-2019_2	GGTGTATACTGCTGCC GTGAAC	22	54.55	61.56
nCoV-2019_4_RIGHT	nCoV-2019_2	CACAAGTAGTGGCAC CTTCTTTAGT	25	44	60.97
nCoV-2019_5_LEFT	nCoV-2019_1	TGGTGAAACTTCATG GCAGACG	22	50	61.39
nCoV-2019_5_RIGHT	nCoV-2019_1	ATTGATGTTGACTTTC TCTTTTTGGAGT	28	32.14	60.17
nCoV-2019_6_LEFT	nCoV-2019_2	GGTGTTGTTGGAGAA GGTTCCG	22	54.55	61.64
nCoV-2019_6_RIGHT	nCoV-2019_2	TAGCGGCCTTCTGTA AAACACG	22	50	61.18
nCoV-2019_7_LEFT	nCoV-2019_1	ATCAGAGGCTGCTCG TGTTGTA	22	50	61.73
nCoV-2019_7_LEFT_ alt0	nCoV-2019_1	CATTTGCATCAGAGG CTGCTCG	22	54.55	62.44
nCoV-2019_7_RIGHT	nCoV-2019_1	TGCACAGGTGACAAT TTGTCCA	22	45.45	60.95
nCoV-2019_7_RIGHT _alt5	nCoV-2019_1	AGGTGACAATTTGTC CACCGAC	22	50	61.07
nCoV-2019_8_LEFT	nCoV-2019_2	AGAGTTTCTTAGAGA CGGTTGGGA	24	45.83	61
nCoV-2019_8_RIGHT	nCoV-2019_2	GCTTCAACAGCTTCA CTAGTAGGT	24	45.83	60.56
nCoV-2019_9_LEFT	nCoV-2019_1	TCCCACAGAAGTGTT AACAGAGGA	24	45.83	61.18
nCoV-2019_9_LEFT_ alt4	nCoV-2019_1	TTCCCACAGAAGTGT TAACAGAGG	24	45.83	60.44
nCoV-2019_9_RIGHT	nCoV-2019_1	ATGACAGCATCTGCC ACAACAC	22	50	61.71
nCoV-2019_9_RIGHT _alt2	nCoV-2019_1	GACAGCATCTGCCAC AACACAG	22	54.55	62.26
nCoV-2019_10_LEFT	nCoV-2019_2	TGAGAAGTGCTCTGC CTATACAGT	24	45.83	61.12
nCoV-2019_10_RIGH T	nCoV-2019_2	TCATCTAACCAATCTT CTTCTTGCTCT	27	37.04	60.31
nCoV-2019_11_LEFT	nCoV-2019_1	GGAATTTGGTGCCAC TTCTGCT	22	50	61.66
nCoV-2019_11_RIGH T	nCoV-2019_1	TCATCAGATTCAACTT GCATGGCA	24	41.67	61.35
nCoV-2019_12_LEFT	nCoV-2019_2	AAACATGGAGGAGG TGTTGCAG	22	50	61.08

nCoV-2019_12_RIGHT	nCoV-2019_2	TTCACTCTTCATTTCCAAAAAGCTTGA	27	33.33	60.36
nCoV-2019_13_LEFT	nCoV-2019_1	TCGCACAAATGTCTACTTAGCTGT	24	41.67	60.56
nCoV-2019_13_RIGHT	nCoV-2019_1	ACCACAGCAGTTAAAACACCCT	22	45.45	60.36
nCoV-2019_14_LEFT	nCoV-2019_2	CATCCAGATTCTGCCACTCTTGT	23	47.83	60.62
nCoV-2019_14_LEFT_alt4	nCoV-2019_2	TGGCAATCTTCATCCAGATTCTGC	24	45.83	61.47
nCoV-2019_14_RIGHT	nCoV-2019_2	AGTTTCCACACAGACAGGCATT	22	45.45	60.42
nCoV-2019_14_RIGHT_alt2	nCoV-2019_2	TGCGTGTTTCTTCTGCATGTGC	22	50	62.76
nCoV-2019_15_LEFT	nCoV-2019_1	ACAGTGCTTAAAAAGTGTAAAAGTGCC	27	37.04	61.32
nCoV-2019_15_LEFT_alt1	nCoV-2019_1	AGTGCTTAAAAAGTGTAAAAGTGCCT	26	34.62	60.13
nCoV-2019_15_RIGHT	nCoV-2019_1	AACAGAAACTGTAGCTGGCACT	22	45.45	60.16
nCoV-2019_15_RIGHT_alt3	nCoV-2019_1	ACTGTAGCTGGCACTTTGAGAGA	23	47.83	61.57
nCoV-2019_16_LEFT	nCoV-2019_2	AATTTGGAAGAAGCTGCTCGGT	22	45.45	60.82
nCoV-2019_16_RIGHT	nCoV-2019_2	CACAACTTGCGTGTGGAGGTTA	22	50	61.32
nCoV-2019_17_LEFT	nCoV-2019_1	CTTCTTTCTTTGAGAGAAGTGAGGACT	27	40.74	60.69
nCoV-2019_17_RIGHT	nCoV-2019_1	TTTGTTGGAGTGTTAACAATGCAGT	25	36	60.11
nCoV-2019_18_LEFT	nCoV-2019_2	TGGAAATACCCACAAGTTAATGGTTTAAC	29	34.48	60.69
nCoV-2019_18_LEFT_alt2	nCoV-2019_2	ACTTCTATTAAATGGGCAGATAACAACTGT	30	33.33	61.38
nCoV-2019_18_RIGHT	nCoV-2019_2	AGCTTGTTTACCACACGTACAAGG	24	45.83	61.51
nCoV-2019_18_RIGHT_alt1	nCoV-2019_2	GCTTGTTTACCACACGTACAAGG	23	47.83	60.3
nCoV-2019_19_LEFT	nCoV-2019_1	GCTGTTATGTACATGGGCACACT	23	47.83	61.18
nCoV-2019_19_RIGHT	nCoV-2019_1	TGTCCAACTTAGGGTCAATTTCTGT	25	40	60.4
nCoV-2019_20_LEFT	nCoV-2019_2	ACAAAGAAAACAGTTACACAACAACCA	27	33.33	60.68
nCoV-2019_20_RIGHT	nCoV-2019_2	ACGTGGCTTTATTAGTTGCATTGTT	25	36	60.28
nCoV-2019_21_LEFT	nCoV-2019_1	TGGCTATTGATTATAAACACTACACACCC	29	37.93	61.49
nCoV-2019_21_LEFT_alt2	nCoV-2019_1	GGCTATTGATTATAAACACTACACACCCT	29	37.93	61.29
nCoV-2019_21_RIGHT	nCoV-2019_1	TAGATCTGTGTGGCCAACCTCT	22	50	60.83
nCoV-2019_21_RIGHT_alt0	nCoV-2019_1	GATCTGTGTGGCCAACCTCTTC	22	54.55	61.2
nCoV-2019_22_LEFT	nCoV-2019_2	ACTACCGAAGTTGTAGGAGACATTATACT	29	37.93	61.25

nCoV-2019_22_RIGHT	nCoV-2019_2	ACAGTATTCTTTGCTATAGTAGTCGGC	27	40.74	60.73
nCoV-2019_23_LEFT	nCoV-2019_1	ACAACTACTAACATAGTTACACGGTGT	27	37.04	60.26
nCoV-2019_23_RIGHT	nCoV-2019_1	ACCAGTACAGTAGGTTGCAATAGTG	25	44	60.57
nCoV-2019_24_LEFT	nCoV-2019_2	AGGCATGCCTTCTTACTGTACTG	23	47.83	60.37
nCoV-2019_24_RIGHT	nCoV-2019_2	ACATTCTAACCATAGCTGAAATCGGG	26	42.31	61.19
nCoV-2019_25_LEFT	nCoV-2019_1	GCAATTGTTTTTCAGCTATTTTGCAGT	27	33.33	60.73
nCoV-2019_25_RIGHT	nCoV-2019_1	ACTGTAGTGACAAGTCTCTCGCA	23	47.83	61.3
nCoV-2019_26_LEFT	nCoV-2019_2	TTGTGATACATTCTGTGCTGGTAGT	25	40	60.28
nCoV-2019_26_RIGHT	nCoV-2019_2	TCCGCACTATCACCAACATCAG	22	50	60.42
nCoV-2019_27_LEFT	nCoV-2019_1	ACTACAGTCAGCTTATGTGTCAACC	25	44	60.8
nCoV-2019_27_RIGHT	nCoV-2019_1	AATACAAGCACCAAGGTCACGG	22	50	61.13
nCoV-2019_28_LEFT	nCoV-2019_2	ACATAGAAGTTACTGGCGATAGTTGT	26	38.46	60.13
nCoV-2019_28_RIGHT	nCoV-2019_2	TGTTTAGACATGACATGAACAGGTGT	26	38.46	60.91
nCoV-2019_29_LEFT	nCoV-2019_1	ACTTGTGTTCCTTTTTGTTGCTGC	24	41.67	61.39
nCoV-2019_29_RIGHT	nCoV-2019_1	AGTGTACTCTATAAGTTTTGATGGTGTGT	29	34.48	60.69
nCoV-2019_30_LEFT	nCoV-2019_2	GCACAACTAATGGTGACTTTTTGCA	25	40	61.19
nCoV-2019_30_RIGHT	nCoV-2019_2	ACCACTAGTAGATACACAAACACCAG	26	42.31	60.3
nCoV-2019_31_LEFT	nCoV-2019_1	TTCTGAGTACTGTAGGCACGGC	22	54.55	62.03
nCoV-2019_31_RIGHT	nCoV-2019_1	ACAGAATAAACACCAGGTAAGAATGAGT	28	35.71	60.69
nCoV-2019_32_LEFT	nCoV-2019_2	TGGTGAATACAGTCATGTAGTTGCC	25	44	61.09
nCoV-2019_32_RIGHT	nCoV-2019_2	AGCACATCACTACGCAACTTTAGA	24	41.67	60.56
nCoV-2019_33_LEFT	nCoV-2019_1	ACTTTTGAAGAAGCTGCGCTGT	22	45.45	61.58
nCoV-2019_33_RIGHT	nCoV-2019_1	TGGACAGTAAACTACGTCATCAAGC	25	44	61.08
nCoV-2019_34_LEFT	nCoV-2019_2	TCCCATCTGGTAAAGTTGAGGGT	23	47.83	61.02
nCoV-2019_34_RIGHT	nCoV-2019_2	AGTGAAATTGGGCCTCATAGCA	22	45.45	60.03
nCoV-2019_35_LEFT	nCoV-2019_1	TGTTCGCATTCAACCAGGACAG	22	50	61.39
nCoV-2019_35_RIGHT	nCoV-2019_1	ACTTCATAGCCACAAGGTTAAAGTCA	26	38.46	60.69
nCoV-2019_36_LEFT	nCoV-2019_2	TTAGCTTGGTTGTACGCTGCTG	22	50	61.44

nCoV-2019_36_RIGHT	nCoV-2019_2	GAACAAAGACCATTGAGTACTCTGGA	26	42.31	60.74
nCoV-2019_37_LEFT	nCoV-2019_1	ACACACCACTGGTTGTTACTCAC	23	47.83	60.93
nCoV-2019_37_RIGHT	nCoV-2019_1	GTCCACACTCTCCTAGCACCAT	22	54.55	61.48
nCoV-2019_38_LEFT	nCoV-2019_2	ACTGTGTTATGTATGCATCAGCTGT	25	40	60.86
nCoV-2019_38_RIGHT	nCoV-2019_2	CACCAAGAGTCAGTCTAAAGTAGCG	25	48	61.13
nCoV-2019_39_LEFT	nCoV-2019_1	AGTATTGCCCTATTTTCTTCATAACTGGT	29	34.48	61
nCoV-2019_39_RIGHT	nCoV-2019_1	TGTAACTGGACACATTGAGCCC	22	50	60.55
nCoV-2019_40_LEFT	nCoV-2019_2	TGCACATCAGTAGTCTTACTCTCAGT	26	42.31	61.25
nCoV-2019_40_RIGHT	nCoV-2019_2	CATGGCTGCATCACGGTCAAAT	22	50	62.09
nCoV-2019_41_LEFT	nCoV-2019_1	GTTCCCTTCCATCATATGCAGCT	23	47.83	60.75
nCoV-2019_41_RIGHT	nCoV-2019_1	TGGTATGACAACCATTAGTTTGGCT	25	40	60.75
nCoV-2019_42_LEFT	nCoV-2019_2	TGCAAGAGATGGTTGTGTTCCC	22	50	61.08
nCoV-2019_42_RIGHT	nCoV-2019_2	CCTACCTCCCTTTGTTGTGTTGT	23	47.83	60.69
nCoV-2019_43_LEFT	nCoV-2019_1	TACGACAGATGTCTTGTGCTGC	22	50	60.93
nCoV-2019_43_RIGHT	nCoV-2019_1	AGCAGCATCTACAGCAAAAGCA	22	45.45	61.14
nCoV-2019_44_LEFT	nCoV-2019_2	TGCCACAGTACGTCTACAAGCT	22	50	61.66
nCoV-2019_44_LEFT_alt3	nCoV-2019_2	CCACAGTACGTCTACAAGCTGG	22	54.55	60.67
nCoV-2019_44_RIGHT	nCoV-2019_2	AACCTTTCCACATACCGCAGAC	22	50	60.87
nCoV-2019_44_RIGHT_alt0	nCoV-2019_2	CGCAGACGGTACAGACTGTGTT	22	54.55	62.77
nCoV-2019_45_LEFT	nCoV-2019_1	TACCTACAACTTGTGCTAATGACCC	25	44	60.57
nCoV-2019_45_LEFT_alt2	nCoV-2019_1	AGTATGTACAAATACCTACAACTTGTGCT	29	34.48	60.94
nCoV-2019_45_RIGHT	nCoV-2019_1	AAATTGTTTCTTCATGTTGGTAGTTAGAGA	30	30	60.01
nCoV-2019_45_RIGHT_alt7	nCoV-2019_1	TTCATGTTGGTAGTTAGAGAAAGTGTGTC	29	37.93	61.53
nCoV-2019_46_LEFT	nCoV-2019_2	TGTCGCTTCCAAGAAAAGGACG	22	50	61.38
nCoV-2019_46_LEFT_alt1	nCoV-2019_2	CGCTTCCAAGAAAAGGACGAAGA	23	47.83	61.35
nCoV-2019_46_RIGHT	nCoV-2019_2	CACGTTCACCTAAGTTGGCGTA	22	50	60.86
nCoV-2019_46_RIGHT_alt2	nCoV-2019_2	CACGTTCACCTAAGTTGGCGTAT	23	47.83	61.17
nCoV-2019_47_LEFT	nCoV-2019_1	AGGACTGGTATGATTTTGTAGAAAACCC	28	39.29	61.42

nCoV-2019_47_RIGHT	nCoV-2019_1	AATAACGGTCAAAGAGTTTTAACCTCTC	28	35.71	60.06
nCoV-2019_48_LEFT	nCoV-2019_2	TGTTGACACTGACTTAACAAAGCCT	25	40	61.09
nCoV-2019_48_RIGHT	nCoV-2019_2	TAGATTACCAGAAGCAGCGTGC	22	50	60.74
nCoV-2019_49_LEFT	nCoV-2019_1	AGGAATTACTTGTGTATGCTGCTGA	25	40	60.57
nCoV-2019_49_RIGHT	nCoV-2019_1	TGACGATGACTTGGTTAGCATTAATACA	28	35.71	61.05
nCoV-2019_50_LEFT	nCoV-2019_2	GTTGATAAGTACTTTGATTGTTACGATGGT	30	33.33	60.59
nCoV-2019_50_RIGHT	nCoV-2019_2	TAACATGTTGTGCCAACCACCA	22	45.45	60.95
nCoV-2019_51_LEFT	nCoV-2019_1	TCAATAGCCGCCACTAGAGGAG	22	54.55	61.34
nCoV-2019_51_RIGHT	nCoV-2019_1	AGTGCATTAACATTGGCCGTGA	22	45.45	61.14
nCoV-2019_52_LEFT	nCoV-2019_2	CATCAGGAGATGCCACAACTGC	22	54.55	61.83
nCoV-2019_52_RIGHT	nCoV-2019_2	GTTGAGAGCAAAATTCATGAGGTCC	25	44	60.62
nCoV-2019_53_LEFT	nCoV-2019_1	AGCAAAATGTTGGACTGAGACTGA	24	41.67	60.69
nCoV-2019_53_RIGHT	nCoV-2019_1	AGCCTCATAAAACTCAGGTTCCC	23	47.83	60.31
nCoV-2019_54_LEFT	nCoV-2019_2	TGAGTTAACAGGACACATGTTAGACA	26	38.46	60.18
nCoV-2019_54_RIGHT	nCoV-2019_2	AACCAAAAACTTGTCCATTAGCACA	25	36	60.11
nCoV-2019_55_LEFT	nCoV-2019_1	ACTCAACTTTACTTAGGAGGTATGAGCT	28	39.29	61.43
nCoV-2019_55_RIGHT	nCoV-2019_1	GGTGTACTCTCCTATTTGTACTTTACTGT	29	37.93	60.54
nCoV-2019_56_LEFT	nCoV-2019_2	ACCTAGACCACCACTTAACCGA	22	50	60.49
nCoV-2019_56_RIGHT	nCoV-2019_2	ACACTATGCGAGCAGAAGGGTA	22	50	61.21
nCoV-2019_57_LEFT	nCoV-2019_1	ATTCTACACTCCAGGGACCACC	22	54.55	61.16
nCoV-2019_57_RIGHT	nCoV-2019_1	GTAATTGAGCAGGGTCGCCAAT	22	50	61.26
nCoV-2019_58_LEFT	nCoV-2019_2	TGATTTGAGTGTTGTCAATGCCAGA	25	40	61.44
nCoV-2019_58_RIGHT	nCoV-2019_2	CTTTTCTCCAAGCAGGGTTACGT	23	47.83	61.06
nCoV-2019_59_LEFT	nCoV-2019_1	TCACGCATGATGTTTCATCTGCA	23	43.48	61.42
nCoV-2019_59_RIGHT	nCoV-2019_1	AAGAGTCCTGTTACATTTTCAGCTTG	26	38.46	60.02
nCoV-2019_60_LEFT	nCoV-2019_2	TGATAGAGACCTTTATGACAAGTTGCA	27	37.04	60.53
nCoV-2019_60_RIGHT	nCoV-2019_2	GGTACCAACAGCTTCTCTAGTAGC	24	50	60.44
nCoV-2019_61_LEFT	nCoV-2019_1	TGTTTATCACCCGCGAAGAAGC	22	50	61.5

nCoV-2019_61_RIGHT	nCoV-2019_1	ATCACATAGACAACAGGTGCGC	22	50	61.25
nCoV-2019_62_LEFT	nCoV-2019_2	GGCACATGGCTTTGAGTTGACA	22	50	61.91
nCoV-2019_62_RIGHT	nCoV-2019_2	GTTGAACCTTTCTACAAGCCGC	22	50	60.35
nCoV-2019_63_LEFT	nCoV-2019_1	TGTTAAGCGTGTTGACTGGACT	22	45.45	60.16
nCoV-2019_63_RIGHT	nCoV-2019_1	ACAAACTGCCACCATCACAACC	22	50	61.85
nCoV-2019_64_LEFT	nCoV-2019_2	TCGATAGATATCCTGCTAATTCCATTGT	28	35.71	60.11
nCoV-2019_64_RIGHT	nCoV-2019_2	AGTCTTGTAAAAGTGTTCCAGAGGT	25	40	60.1
nCoV-2019_65_LEFT	nCoV-2019_1	GCTGGCTTTAGCTTGTGGGTTT	22	50	61.92
nCoV-2019_65_RIGHT	nCoV-2019_1	TGTCAGTCATAGAACAAACACCAATAGT	28	35.71	60.9
nCoV-2019_66_LEFT	nCoV-2019_2	GGGTGTGGACATTGCTGCTAAT	22	50	61.21
nCoV-2019_66_RIGHT	nCoV-2019_2	TCAATTTCCATTTGACTCCTGGGT	24	41.67	60.45
nCoV-2019_67_LEFT	nCoV-2019_1	GTTGTCCAACAATTACCTGAAACTTACT	28	35.71	60.43
nCoV-2019_67_RIGHT	nCoV-2019_1	CAACCTTAGAAACTACAGATAAATCTTGGG	30	36.67	60.4
nCoV-2019_68_LEFT	nCoV-2019_2	ACAGGTTCATCTAAGTGTGTGTGT	24	41.67	60.14
nCoV-2019_68_RIGHT	nCoV-2019_2	CTCCTTTATCAGAACCAGCACCA	23	47.83	60.31
nCoV-2019_69_LEFT	nCoV-2019_1	TGTCGCAAAATATACTCAACTGTGTCA	27	37.04	61.43
nCoV-2019_69_RIGHT	nCoV-2019_1	TCTTTATAGCCACGGAACCTCCA	23	47.83	61.14
nCoV-2019_70_LEFT	nCoV-2019_2	ACAAAAGAAAATGACTCTAAAGAGGGTTT	29	31.03	60.13
nCoV-2019_70_RIGHT	nCoV-2019_2	TGACCTTCTTTTAAAGACATAACAGCAG	28	35.71	60.27
nCoV-2019_71_LEFT	nCoV-2019_1	ACAAATCCAATTCAGTTGTCTTCCTATTC	29	34.48	60.54
nCoV-2019_71_RIGHT	nCoV-2019_1	TGGAAAAGAAAGGTAAGAACAAGTCCT	27	37.04	60.8
nCoV-2019_72_LEFT	nCoV-2019_2	ACACGTGGTGTTTATTACCCTGAC	24	45.83	61.04
nCoV-2019_72_RIGHT	nCoV-2019_2	ACTCTGAACTCACTTTCCATCCAAC	25	44	60.97
nCoV-2019_73_LEFT	nCoV-2019_1	CAATTTTGTAATGATCCATTTTTGGGTGT	29	31.03	60.29
nCoV-2019_73_RIGHT	nCoV-2019_1	CACCAGCTGTCCAACCTGAAGA	22	54.55	62.45
nCoV-2019_74_LEFT	nCoV-2019_2	ACATCACTAGGTTTCAAACTTTACTTGC	28	35.71	60.68
nCoV-2019_74_RIGHT	nCoV-2019_2	GCAACACAGTTGCTGATTCTCTTC	24	45.83	60.85
nCoV-2019_75_LEFT	nCoV-2019_1	AGAGTCCAACCAACAGAATCTATTGT	26	38.46	60.24

nCoV-2019_75_RIGHT	nCoV-2019_1	ACCACCAACCTTAGAATCAAGATTGT	26	38.46	60.69
nCoV-2019_76_LEFT	nCoV-2019_2	AGGGCAAACTGGAAAGATTGCT	22	45.45	60.76
nCoV-2019_76_LEFT_alt3	nCoV-2019_2	GGGCAAACTGGAAAGATTGCTGA	23	47.83	61.87
nCoV-2019_76_RIGHT	nCoV-2019_2	ACACCTGTGCCTGTTAAACCAT	22	45.45	60.42
nCoV-2019_76_RIGHT_alt0	nCoV-2019_2	ACCTGTGCCTGTTAAACCATTGA	23	43.48	60.69
nCoV-2019_77_LEFT	nCoV-2019_1	CCAGCAACTGTTTGTGGACCTA	22	50	60.75
nCoV-2019_77_RIGHT	nCoV-2019_1	CAGCCCCTATTAAACAGCCTGC	22	54.55	61.59
nCoV-2019_78_LEFT	nCoV-2019_2	CAACTTACTCCTACTTGGCGTGT	23	47.83	60.55
nCoV-2019_78_RIGHT	nCoV-2019_2	TGTGTACAAAAACTGCCATATTGCA	25	36	60.22
nCoV-2019_79_LEFT	nCoV-2019_1	GTGGTGATTCAACTGAATGCAGC	23	47.83	60.92
nCoV-2019_79_RIGHT	nCoV-2019_1	CATTTCATCTGTGAGCAAAGGTGG	24	45.83	60.62
nCoV-2019_80_LEFT	nCoV-2019_2	TTGCCTTGGTGATATTGCTGCT	22	45.45	60.89
nCoV-2019_80_RIGHT	nCoV-2019_2	TGGAGCTAAGTTGTTTAACAAGCG	24	41.67	60.02
nCoV-2019_81_LEFT	nCoV-2019_1	GCACTTGGAAAACTTCAAGATGTGG	25	44	61.24
nCoV-2019_81_RIGHT	nCoV-2019_1	GTGAAGTTCTTTTCTTGTGCAGGG	24	45.83	60.73
nCoV-2019_82_LEFT	nCoV-2019_2	GGGCTATCATCTTATGTCCTTCCCT	25	48	61.52
nCoV-2019_82_RIGHT	nCoV-2019_2	TGCCAGAGATGTCACCTAAATCAA	24	41.67	60.02
nCoV-2019_83_LEFT	nCoV-2019_1	TCCTTTGCAACCTGAATTAGACTCA	25	40	60.46
nCoV-2019_83_RIGHT	nCoV-2019_1	TTTGACTCCTTTGAGCACTGGC	22	50	61.33
nCoV-2019_84_LEFT	nCoV-2019_2	TGCTGTAGTTGTCTCAAGGGCT	22	50	61.61
nCoV-2019_84_RIGHT	nCoV-2019_2	AGGTGTGAGTAAACTGTTACAAACAAC	27	37.04	60.36
nCoV-2019_85_LEFT	nCoV-2019_1	ACTAGCACTCTCCAAGGGTGTT	22	50	61.03
nCoV-2019_85_RIGHT	nCoV-2019_1	ACACAGTCTTTTACTCCAGATTCCC	25	44	60.51
nCoV-2019_86_LEFT	nCoV-2019_2	TCAGGTGATGGCACAACAAGTC	22	50	61.07
nCoV-2019_86_RIGHT	nCoV-2019_2	ACGAAAGCAAGAAAAAGAAGTACGC	25	40	61.01
nCoV-2019_87_LEFT	nCoV-2019_1	CGACTACTAGCGTGCCTTTGTA	22	50	60.16
nCoV-2019_87_RIGHT	nCoV-2019_1	ACTAGGTTCCATTGTTCAAGGAGC	24	45.83	60.81
nCoV-2019_88_LEFT	nCoV-2019_2	CCATGGCAGATTCCAACGGTAC	22	54.55	61.58

nCoV-2019_88_RIGHT	nCoV-2019_2	TGGTCAGAATAGTGCCATGGAGT	23	47.83	61.4
nCoV-2019_89_LEFT	nCoV-2019_1	GTACGCGTTCCATGTGGTCATT	22	50	61.5
nCoV-2019_89_LEFT_alt2	nCoV-2019_1	CGCGTTCCATGTGGTCATTCAA	22	50	62.01
nCoV-2019_89_RIGHT	nCoV-2019_1	ACCTGAAAGTCAACGAGATGAAACA	25	40	60.91
nCoV-2019_89_RIGHT_alt4	nCoV-2019_1	ACGAGATGAAACATCTGTTGTCACT	25	40	60.74
nCoV-2019_90_LEFT	nCoV-2019_2	ACACAGACCATTCCAGTAGCAGT	23	47.83	61.58
nCoV-2019_90_RIGHT	nCoV-2019_2	TGAAATGGTGAATTGCCCTCGT	22	45.45	60.82
nCoV-2019_91_LEFT	nCoV-2019_1	TCACTACCAAGAGTGTGTTAGAGGT	25	44	60.93
nCoV-2019_91_RIGHT	nCoV-2019_1	TTCAAGTGAGAACCAAAAGATAATAAGCA	29	31.03	60.03
nCoV-2019_92_LEFT	nCoV-2019_2	TTTGTGCTTTTTAGCCTTTCTGCT	24	37.5	60.14
nCoV-2019_92_RIGHT	nCoV-2019_2	AGGTTCCTGGCAATTAATTGTAAAAGG	27	37.04	60.53
nCoV-2019_93_LEFT	nCoV-2019_1	TGAGGCTGGTTCTAAATCACCCA	23	47.83	61.59
nCoV-2019_93_RIGHT	nCoV-2019_1	AGGTCTTCCTTGCCATGTTGAG	22	50	60.55
nCoV-2019_94_LEFT	nCoV-2019_2	GGCCCCAAGGTTTACCCAATAA	22	50	60.56
nCoV-2019_94_RIGHT	nCoV-2019_2	TTTGGCAATGTTGTTCCTTGAGG	23	43.48	60.18
nCoV-2019_95_LEFT	nCoV-2019_1	TGAGGGAGCCTTGAATACACCA	22	50	61.1
nCoV-2019_95_RIGHT	nCoV-2019_1	CAGTACGTTTTTGCCGAGGCTT	22	50	61.95
nCoV-2019_96_LEFT	nCoV-2019_2	GCCAACAACAACAAGGCCAAAC	22	50	61.82
nCoV-2019_96_RIGHT	nCoV-2019_2	TAGGCTCTGTTGGTGGGAATGT	22	50	61.36
nCoV-2019_97_LEFT	nCoV-2019_1	TGGATGACAAAGATCCAAATTTCAAAGA	28	32.14	60.22
nCoV-2019_97_RIGHT	nCoV-2019_1	ACACACTGATTAAAGATTGCTATGTGAG	28	35.71	60.17
nCoV-2019_98_LEFT	nCoV-2019_2	AACAATTGCAACAATCCATGAGCA	24	37.5	60.5
nCoV-2019_98_RIGHT	nCoV-2019_2	TTCTCCTAAGAAGCTATTAAAATCACATGG	30	33.33	60.01

Supplementary Table 4. We used the ARTIC Network v3 primers to amplify cDNA. The primer names, primer pools, sequences, % GC content, and ideal primer melting temp are included. These primers can also be found on the 'artic-network' GitHub page – https://github.com/artic-network/primer-schemes/tree/master/nCoV-2019/V3.
