# Supplementary material for: Revealing fine-scale spatiotemporal differences in SARS-CoV-2 introduction and spread
Source: Nat Commun. 2020 Nov 3;11:5558. doi: 10.1038/s41467-020-19346-z (PMC7609670; doi:10.1038/s41467-020-19346-z)
Supplement: Supplementary file 2 — Reporting Summary [file 41467_2020_19346_MOESM2_ESM.pdf]

nature research

Corresponding author(s): Thomas Friedrich

Last updated by author(s): Sep 21, 2020

Reporting Summary

Nature Research wishes to improve the reproducibility of the work that we publish. This form provides structure for consistency and transparency in reporting. For further information on Nature Research policies, see our Editorial Policies and the Editorial Policy Checklist.

Statistics

For all statistical analyses, confirm that the following items are present in the figure legend, table legend, main text, or Methods section.

n/a	Confirmed	
☐	☒	The exact sample size (*n*) for each experimental group/condition, given as a discrete number and unit of measurement
☒	☐	A statement on whether measurements were taken from distinct samples or whether the same sample was measured repeatedly
☒	☐	The statistical test(s) used AND whether they are one- or two-sided<br>*Only common tests should be described solely by name; describe more complex techniques in the Methods section.*
☒	☐	A description of all covariates tested
☐	☒	A description of any assumptions or corrections, such as tests of normality and adjustment for multiple comparisons
☐	☒	A full description of the statistical parameters including central tendency (e.g. means) or other basic estimates (e.g. regression coefficient) AND variation (e.g. standard deviation) or associated estimates of uncertainty (e.g. confidence intervals)
☒	☐	For null hypothesis testing, the test statistic (e.g. *F*, *t*, *r*) with confidence intervals, effect sizes, degrees of freedom and *P* value noted<br>*Give P values as exact values whenever suitable.*
☐	☒	For Bayesian analysis, information on the choice of priors and Markov chain Monte Carlo settings
☒	☐	For hierarchical and complex designs, identification of the appropriate level for tests and full reporting of outcomes
☒	☐	Estimates of effect sizes (e.g. Cohen's *d*, Pearson's *r*), indicating how they were calculated

Our web collection on statistics for biologists contains articles on many of the points above.

Software and code

Policy information about availability of computer code

Data collection	We generated viral deep sequence data from original nasopharyngeal swabs were sequenced on the Oxford Nanopore GridION Platform and base-called in real time using Guppy 3.2.6.
Data analysis	Raw sequence data were analyzed to generate consensus sequences using a modified version of the ARTIC bioinformatic pipeline (available here: https://github.com/gagekmoreno/SARS-CoV-2-in-Southern-Wisconsin, filename = artic_rt.zip using reference genome Genbank MN908947.3). This pipeline uses Minimap2 v2.17 and Medaka 1.03 (https://github.com/nanoporetech/medaka) to generate consensus sequences. VCF files denoting consensus SNV differences compared to the reference were cleaned using custom Python scripts and visualized using Matplotlib 3.3.2 (https://matplotlib.org). These Python scripts in Jupyter Notebook format as well as the raw and cleaned VCF files can be found at https://github.com/gagekmoreno/SARS-CoV-2-in-Southern-Wisconsin/tree/master/consensus_SNVs. Phylogenetic trees were built using Nextstrain tools and clade nomenclature (https://github.com/nextstrain/ncov). All scripts and output files for the Wisconsin-only builds can be found at https://github.com/gagekmoreno/SARS-CoV-2-in-Southern-Wisconsin/tree/master/builds. The global sub-sampled trees were generated using MAFFT v7.464 (https://mafft.cbrc.jp/alignment/software/), FastTree v2.1.10 (PMID: 20224823), and IQ-TREE v1.5.5 (http://www.iqtree.org), and are available at http://github.com/roblanf/sarscov2phylo/. All scripts and output files for the sub-sampled global build can be found at https://github.com/gagekmoreno/SARS-CoV-2-in-Southern-Wisconsin/tree/master/builds/Global_Sub_Build. Results were visualized using Matplotlib 3.3.2(https://matplotlib.org), Seaborn v0.10.0 (https://github.com/mwaskom/seaborn), and Baltic v0.1.0 (https://github.com/evogytis/baltic). Bayesian phylogenetic inference and dynamic modelling were performed with BEAST2 software (v2.6.2) (PMID: 30958812) and the PhyDyn package (v1.3.6) (PMID: 30422979). Parameter traces were visualized in Tracer (v1.7.1) (http://tree.bio.ed.ac.uk/software/tracer/). Trajectory files from duplicate runs were merged with an in-house R script available at the GitHub repository https://github.com/gagekmoreno/SARS-CoV-2-in-Southern-Wisconsin.

For manuscripts utilizing custom algorithms or software that are central to the research but not yet described in published literature, software must be made available to editors and reviewers. We strongly encourage code deposition in a community repository (e.g. GitHub). See the Nature Research guidelines for submitting code & software for further information.

Data

Policy information about availability of data

All manuscripts must include a data availability statement. This statement should provide the following information, where applicable:

- Accession codes, unique identifiers, or web links for publicly available datasets
- A list of figures that have associated raw data
- A description of any restrictions on data availability

Sequencing data after mapping to SARS-CoV-2 reference genome (Genbank: MN908947.3) have been deposited in the Sequence Read Archive (SRA) under bioproject PRJNA614504. All sequences have also been uploaded to Global Initiative on Sharing Avian Influenza Data (GISAID). Associated GISAID accession numbers can be found in supplemental information. Derived data, analysis pipelines, and figures have been made available for easy replication of these results at a publicly-accessible GitHub repository: https://github.com/gagekmoreno/SARS-CoV-2-in-Southern-Wisconsin.

We obtained a county-level map of Wisconsin from the State Cartographer's Office (https://www.sco.wisc.edu/maps/wisconsin-outline/). We obtained Wisconsin county-level COVID-19 cumulative case data from the Wisconsin Department of Health Services COVID-19 dashboard (https://data.dhsgis.wi.gov/datasets/covid-19-historical-data-table/, https://cityofmadison.maps.arcgis.com/apps/opsdashboard/index.html#/e22f5ba4f1f94e0bb0b9529dc82db6a3, and https://county.milwaukee.gov/EN/COVID-19). All Dane and Milwaukee county demographic data came from the Wisconsin Department of Health Services Data & Statistics (https://www.dhs.wisconsin.gov/stats) or the U.S. Census Bureau QuickFacts table (https://www.census.gov/quickfacts/fact/table/).

All sequences have been uploaded to GISAID and GenBank. Associated accession numbers can be found in supplementary information 1 file.

Field-specific reporting

Please select the one below that is the best fit for your research. If you are not sure, read the appropriate sections before making your selection.

☒ Life sciences ☐ Behavioural & social sciences ☐ Ecological, evolutionary & environmental sciences

For a reference copy of the document with all sections, see nature.com/documents/nr-reporting-summary-flat.pdf

Life sciences study design

All studies must disclose on these points even when the disclosure is negative.

Sample size	This study was not designed prospectively. We included all SARS-CoV-2 sequences originating from samples collected in Dane County from 14 of March through 18 of April (n=122) and all sequences originating from samples collected from Milwaukee County from 15 of March through 25 of April (n=125). We did not predetermine sample sizes, rather we established a sampled collection period and included all sequences which were available and sequenced in this time period. Sample sizes were determined as the absolute number of consensus sequences available throughout the determined time period.
Data exclusions	No data were excluded here. We included all high-quality consensus sequences generated from the established sampling period (14 March through 25 April) originating from the counties of interest (Dane County and Milwaukee County).
Replication	All raw sequence data are available on GISAID and GenBank (supplemental information 1) and all code used to manipulate these data are available at https://github.com/gagekmoreno/SARS-CoV-2-in-Southern-Wisconsin. Bootstrap analysis was conducted on phylogenetic tree approximation. 1000 bootstraps were achieved successfully. Each introduction analysis was supported by 100 bootstrap replicates. Each BEAST analysis was run in duplicate for at least 3 million states in BEAST2 and was further confirmed by a sensitivity analysis.
Randomization	Experimental randomization was not applicable to this study. Sequences were grouped by collection date and geolocation.
Blinding	Binding was not applicable to this study. Sequence metadata were uploaded to public repositories in as real-time as possible to facilitate open data sharing in the setting of global pandemic.

Reporting for specific materials, systems and methods

We require information from authors about some types of materials, experimental systems and methods used in many studies. Here, indicate whether each material, system or method listed is relevant to your study. If you are not sure if a list item applies to your research, read the appropriate section before selecting a response.

Materials & experimental systems

n/a	Involved in the study
[x]	[ ] Antibodies
[x]	[ ] Eukaryotic cell lines
[x]	[ ] Palaeontology and archaeology
[x]	[ ] Animals and other organisms
[ ]	[x] Human research participants
[x]	[ ] Clinical data
[x]	[ ] Dual use research of concern

Methods

n/a	Involved in the study
[x]	[ ] ChIP-seq
[x]	[ ] Flow cytometry
[x]	[ ] MRI-based neuroimaging

Human research participants

Policy information about studies involving human research participants

Population characteristics	We were blinded to any of the patient information other than SARS-CoV-2 diagnosis status and county of residence. If a patient tested positive for SARS-CoV-2, the residual virus sample was included in the analysis for this manuscript. As noted in the manuscript, this study was not designed prospectively but rather takes advantage of our surveillance sequencing efforts in the defined time period.
Recruitment	There was no recruitment for this manuscript as this is not considered human research. As stated above, if a patient received a confirmed SARS-CoV-2 diagnosis, their residual swab sample was used in this analysis.
Ethics oversight	Western Institutional Review Board's (WIRB's) IRB Affairs Department reviewed the study under the Common Rule and applicable guidance. The WIRB believes the study is exempt under 45 CFR § 46.104(d)(4), because the research involves the use of identifiable private information/biospecimens; and information, which may include information about biospecimens, is recorded by the investigator in such a manner that the identity of the human subjects cannot readily by ascertained directly or through identifiers linked to the subjects, the investigator does not contact the subjects, and the investigator will not re-identify subjects.

Note that full information on the approval of the study protocol must also be provided in the manuscript.
