# Supplementary figures and images for: Revealing fine-scale spatiotemporal differences in SARS-CoV-2 introduction and spread
Source: Nat Commun. 2020 Nov 3;11:5558. doi: 10.1038/s41467-020-19346-z (PMC7609670; doi:10.1038/s41467-020-19346-z)

**A**

Dane County  
Milwaukee County  
Wisconsin

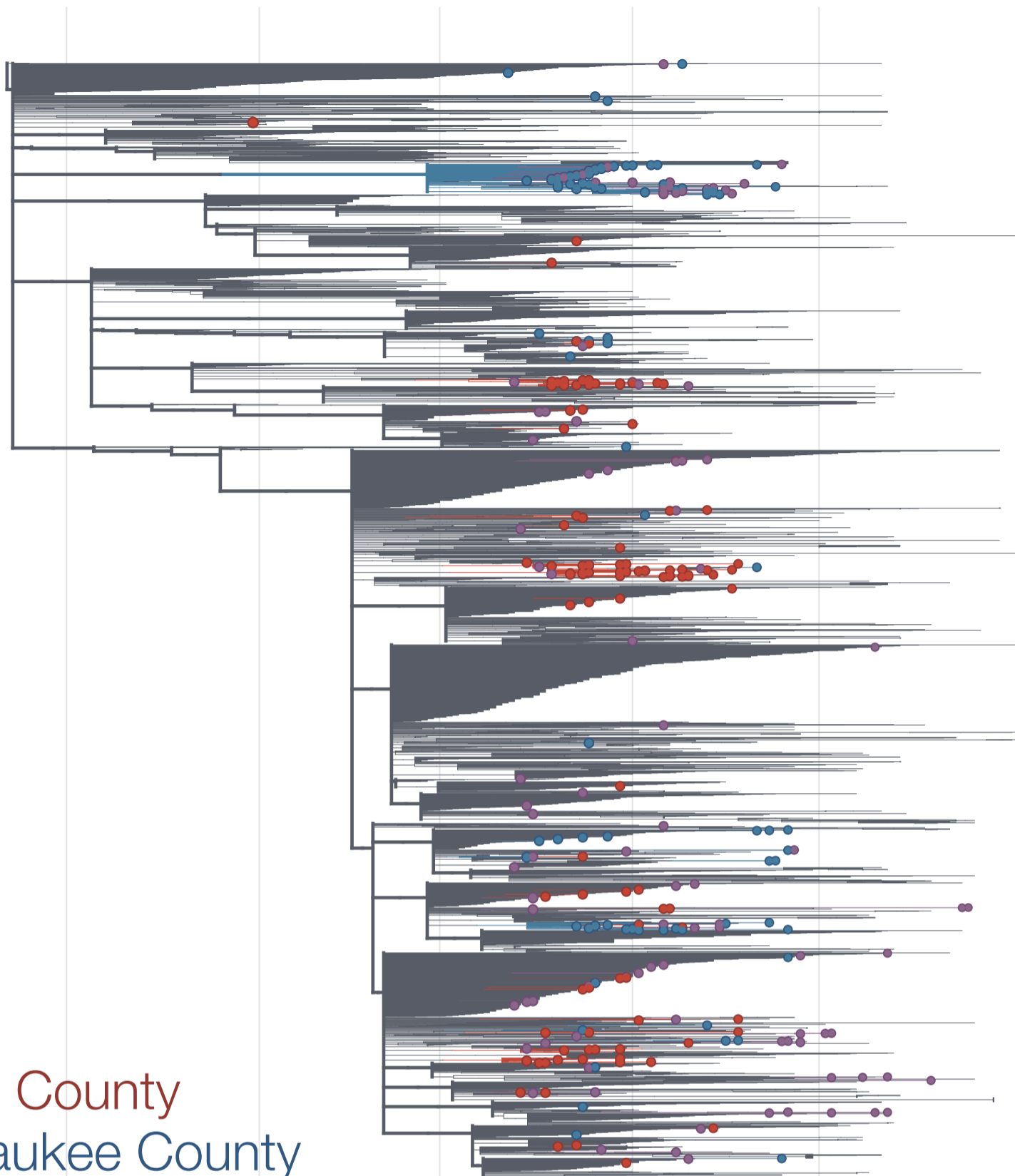**B**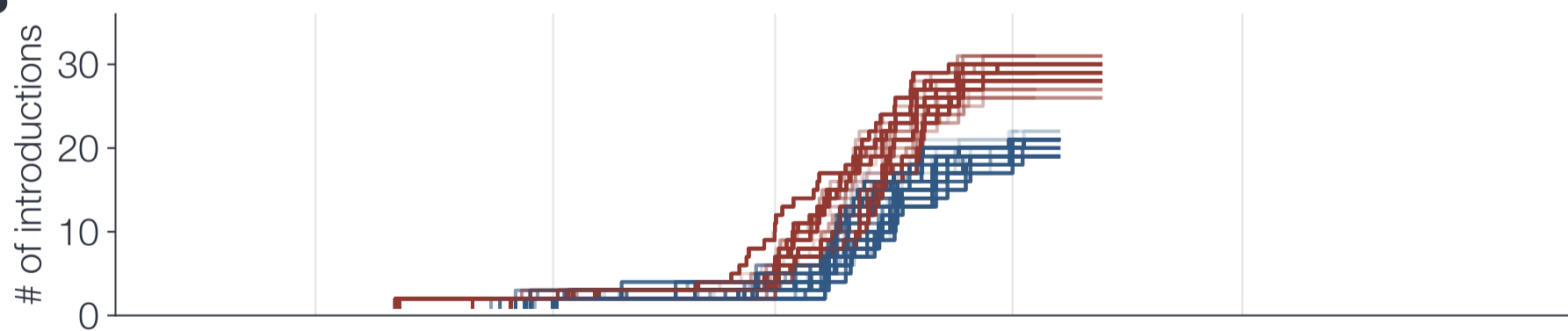**C**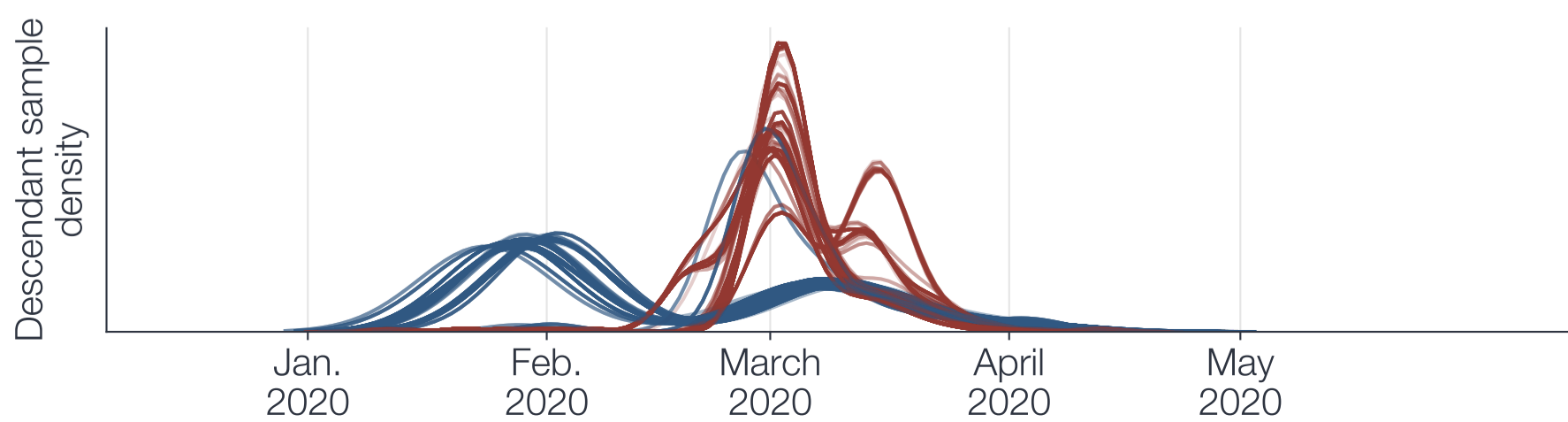

Supplement: Supplementary file 3 — Source Data [file 41467_2020_19346_MOESM3_ESM.zip › Source Data/introductions/figures/tree_timeseries.pdf]

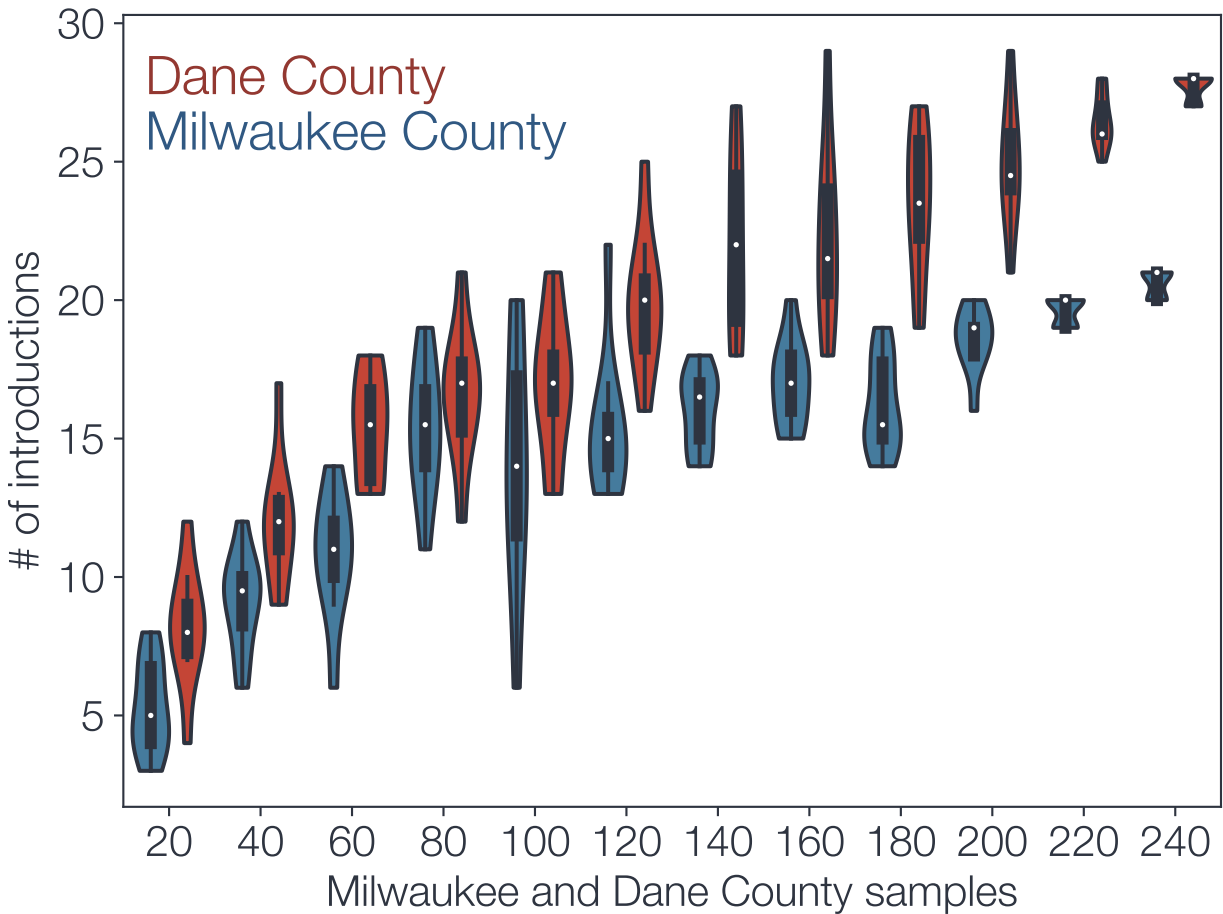

Supplement: Supplementary file 3 — Source Data [file 41467_2020_19346_MOESM3_ESM.zip › Source Data/introductions/figures/rarefaction.pdf]
